# Total and engineered biosynthesis of cinatrins: inhibitors of phospholipase A_2_ and squalene synthase

**DOI:** 10.1039/d6sc00660d

**Published:** 2026-05-08

**Authors:** Henrike Heinemann, Nick Gerlach, Eric Kuhnert, Jennifer Gerke, Bart Verwaaijen, Jörn Kalinowski, Marc Stadler, Russell J. Cox

**Affiliations:** a Institute for Organic Chemistry and BMWZ, Leibniz Universität Hannover Schneiderberg 38 30167 Hannover Germany russell.cox@oci.uni-hannover.de; b CeBiTec, Universität Bielefeld, Universitätsstraße 27 D-33615 Bielefeld Germany; c Helmholtz Zentrum für Infektionsforschung Inhoffenstraße 7 38124 Braunschweig Germany

## Abstract

An alkyl citrate biosynthetic gene cluster (*ctr*) was identified from 47 members of the fungal family of Hypoxylaceae by genome mining. The *Hypomontagnella monticulosa* MUCL 54604 *ctr* cluster was deployed for the total biosynthesis of the known specialised metabolites CJ-13,982 and cinatrins C_1_ and C_3_. The required 2- and 4- oxygenations were catalysed by an α-ketoglutarate-dependent non-heme iron dioxygenase that is homologous to known oxygenases including MfR1 and MfR2 from the squalestatin biosynthetic pathway. Combinatorial biosynthesis, and *in vitro* experiments including the oxygenases MfR1 and MfR2 from the squalestatin S1 biosynthetic gene cluster, were used for the synthesis of new natural products with bioactivity against phospholipase A_2_ and squalene synthase.

## Abstract 2

An alkyl citrate biosynthetic gene cluster (*ctr*) was identified from 47 members of the fungal family of Hypoxylaceae by genome mining.

## Graphical abstract

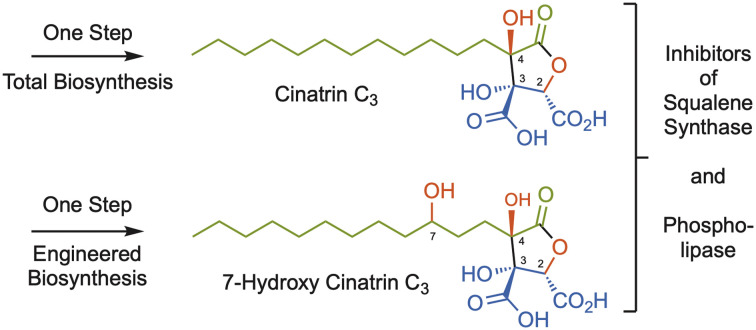

## Introduction

**Scheme 1:**
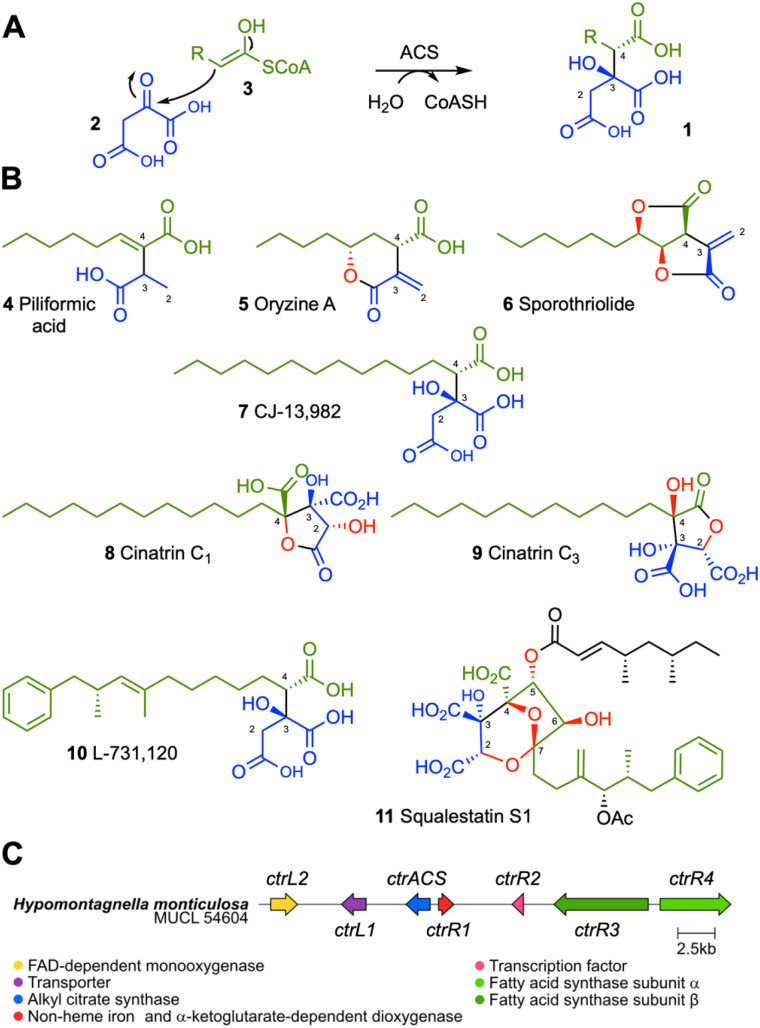
Alkyl citrates: (A), alkyl citrate formation catalyzed by ACS; (B), fungal alkyl citrates. Structural parts derived from oxaloacetate 2 are shown in blue, the fatty acid or polyketide chains are shown in green, red atoms derive from O_2_; (C), the *ctr* BGC.

Alkyl citrates 1 are a class of specialized metabolites derived from condensation of oxaloacetate 2 with fatty acyl or polyketide CoA thiolesters 3, catalyzed by alkyl citrate synthase (ACS) enzymes (Scheme 1A).^1^ Alkyl citrates are common metabolites of fungi and examples of the class include piliformic acid 4,^2^ and oryzine A 5.^3^ Many alkyl citrates including sporothriolide 6,^4,5^ CJ-13,982 7,^6^ cinatrins C_1_8 and C_3_9,^7,8^ possess interesting bioactivities (Scheme 1B). For example, CJ-13,982 7 is a µM inhibitor of human squalene synthase (SQS) with potential use as an anti-cholesterol compound, while the cinatrins are a family of inhibitors of phospholipase A_2_ (PLA_2_) with potential use as anti-inflammatory agents. Cinatrins were first identified in the fungal strain *Circinotrichum falcatisporum* RF-641 in 1992, but their biosynthetic origin has remained elusive.^7,8^ An interesting alkyl citrate metabolite is L-731,120 10 (ref. 9 and 10) that is a precursor of squalestatin S1 11. L-731,120 10 possesses 0.26 µM inhibitory activity *vs.* rat SQS.^11^ However, 11 is a very potent pM inhibitor of rat SQS due to its distinctive 4,8-dioxabicyclo-[3.2.1]octane core that arises due to selective oxygenation of 10 at carbons C-2, and C-4 to C-7, followed by cyclisation.

The useful bioactivity of alkyl citrates such as cinatrins has resulted in many reported total chemical synthesis campaigns for this class of compounds.^12–16^ However, chemical synthesis of cinatrins and related compounds is challenging due to the requirement for the selective construction of contiguous stereocenters. Moreover, generation of the highly oxidized triacid core usually requires sophisticated protecting group strategies, adding steps and reducing yields.^17^ For example the most recent chemical synthesis of 8 involves 15 linear chemical steps and *ca* 30 different reagents and solvents.^16^ Total chemical synthesis is also highly carbon-intensive.^18,19^ As the requirement for all sectors of the economy to decarbonize becomes increasingly urgent,^20^ it would be highly beneficial to find short, efficient and effective synthetic routes to new bioactive compounds such as alkyl citrates. Here we focus on the use of a biosynthetic system for the one-step syntheses of cinatrins and related compounds that are alkyl citrates with known bioactivities *vs.* PLA_2_ and SQS.

## Results and discussion

We previously identified a highly conserved cryptic alkyl citrate biosynthetic gene cluster (BGC) on the genomes of 13 fungi from the Hypoxylaceae family.^21^ The BGC was predicted to encode two fatty acid synthase (FAS) subunits, an ACS, a 2-oxoglutarate-dependent non-heme iron (NHI) dioxygenase and an FAD-dependent monooxygenase (FMO) as putative structural genes. However, no product could be linked to the BGC. The genome sequences of 34 additional Hypoxylaceae family members were obtained using combinations of Illumina with Oxford Nanopore or PacBio sequencing technologies. This afforded high-quality genome sequences for each additional organism (N50 values between 1.2 Mbp and 9.2 Mbp and a contig count ranging from 9 to 122).

The *Hypomontagnella monticulosa* ACS BGC identified in our previous study was used in BLAST searches *vs.* the translated nucleotide database of the targeted organism to find homologous BGCs in the new genomes.^22^ A homologous BGC encoding the FASα (*ctrR4*) and FASβ (*ctrR3*) components, the ACS (*ctrACS*) and the NHI (*ctrR1*) and FMO (*ctrL2*) oxygenases and putative transporter (*ctrL1*) and transcription factor (*ctrR2*) was detected in all 47 Hypoxylaceae genomes (SI Fig. S1) and designated as the *ctr* BGC (Scheme 1C). Clinker analysis^23^ revealed that the putative *ctr* biosynthetic genes are highly conserved within syntenic genomic regions (SI Fig. S1). BLASTp analysis of the NHI enzyme CtrR1 showed significant homology between it and the MfR1 (45%) and MfR2 (38%) NHI oxygenases from the squalestatin S1 11 biosynthetic pathway (SI Fig. S2).^10^ None of the new Hypoxylaceae genomes contained the sporothriolide BGC cluster previously only found in the *Hypomontagnella* species.^4^

**Fig. 1:**
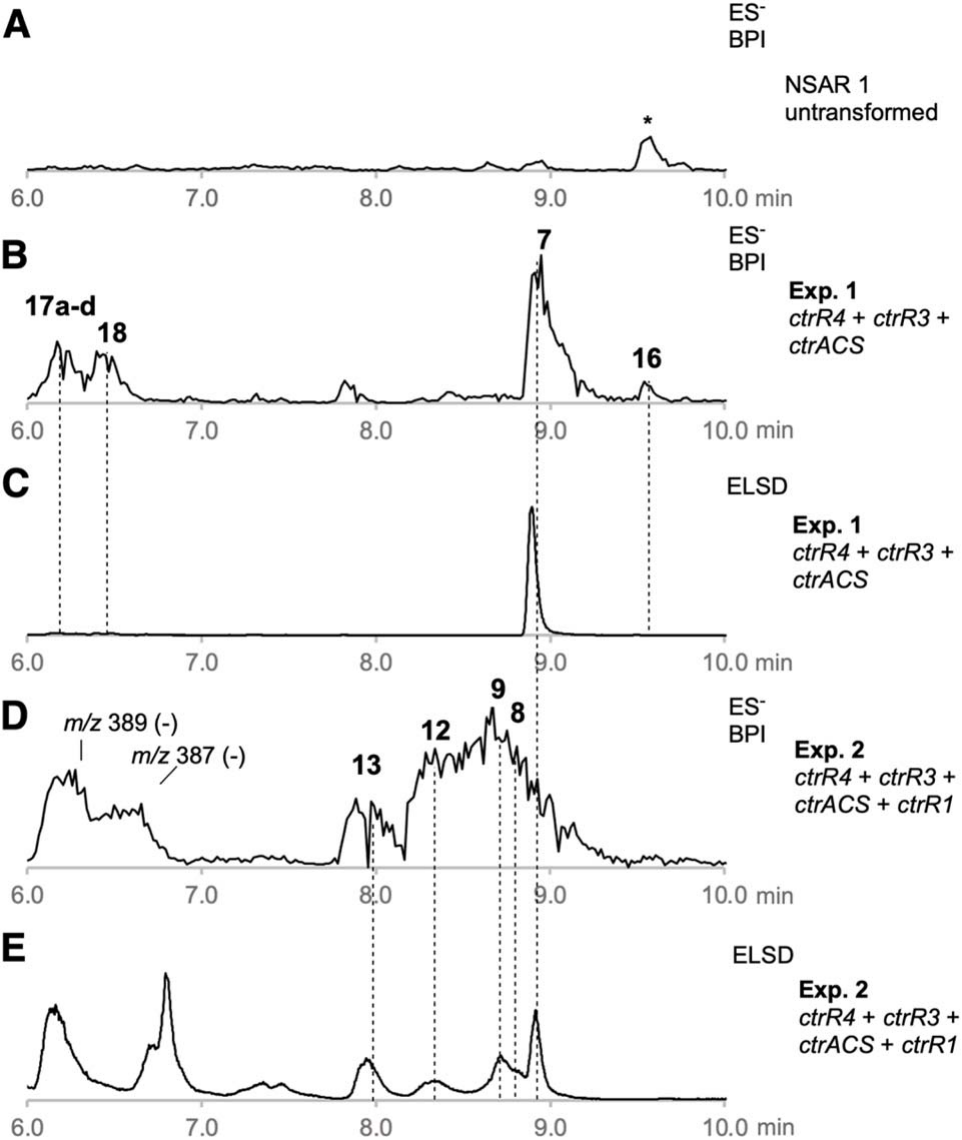
LCMS analysis of organic extracts from experiments 1 and 2: (A), untransformed *A. oryzae* (ES^−^, BPI); (B), *A. oryzae* + *ctrR3* + *ctrR4* + *ctrACS* (ES^−^, BPI); (C), *A. oryzae* + *ctrR3* + *ctrR4* + *ctrACS* (ELSD); (D), *A. oryzae* + *ctrR3* + *ctrR4* + *ctrACS* + *ctrR1* (ES^−^, BPI); (E), *A. oryzae* + *ctrR3* + *ctrR4* + *ctrACS* + *ctrR1* (ELSD); * = unrelated compound.

We reasoned that strategic expression of genes from the *ctr* BGC and elsewhere could be used for the rational synthesis of both known and new alkyl citrates. In an initial experiment *ctrR4*, *ctrR3*, and *ctrACS* were each cloned downstream of strong fungal promoters in the Lazarus vector system.^24^ These vectors were stably integrated into the genome of *A. oryzae* NSAR1,^25^ and after selection and genetic validation transformants were grown in inducing media. After 7 days of growth the fungal cultures were extracted with EtOAc and the extracts interrogated by LCMS (Table 1, Exp1). These transformants produced CJ-13,982 7 as the major metabolite (12.5 mg L^−1^), that was not produced by untransformed *A. oryzae* (Fig. 1A–C).

**Table 1:** Synthesis of alkyl citrates *in vivo* and *in vitro*

Exp	*in vivo*	*in vitro*	7	*ctrR3*	*ctrR4*	*ctrACS*	*ctrR1*	*ctrL2*	*mfR1*	*mfR2*	Characterized products	Data in Fig.
				FASA	FASB	ACS	NHI	FMO	NHI	NHI		
1	✓	—	—	✓	✓	✓	—	—	—	—	7	S5
2a	✓	—	—	✓	✓	✓	✓	—	—	—	7, 8, 9, 12	S32
2b	✓	—	—	✓	✓	✓	✓	✓	—	—	7, 8, 9, 12	S33
3	—	✓	✓	—	—	—	✓	—	—	—	8, 9, 12	S46
4	—	✓	✓	—	—	—	—	—	✓	—	12	S48
5	—	✓	✓	—	—	—	—	—	—	✓	14	S49–51
6	—	✓	✓	—	—	—	—	—	✓	✓	Suggestions in SI	S58
7	✓	—	—	✓	✓	✓	—	—	✓	—	7, 8, 9, 12	S59
8	✓	—	—	✓	✓	✓	—	—	—	✓	7, 14	S60
9	✓	—	—	✓	✓	✓	—	—	✓	✓	7, 12, 15	S61
10	✓	—	—	✓	✓	✓	✓	—	✓	✓	7, 8, 9, 12, 15	S62

**Scheme 2:**
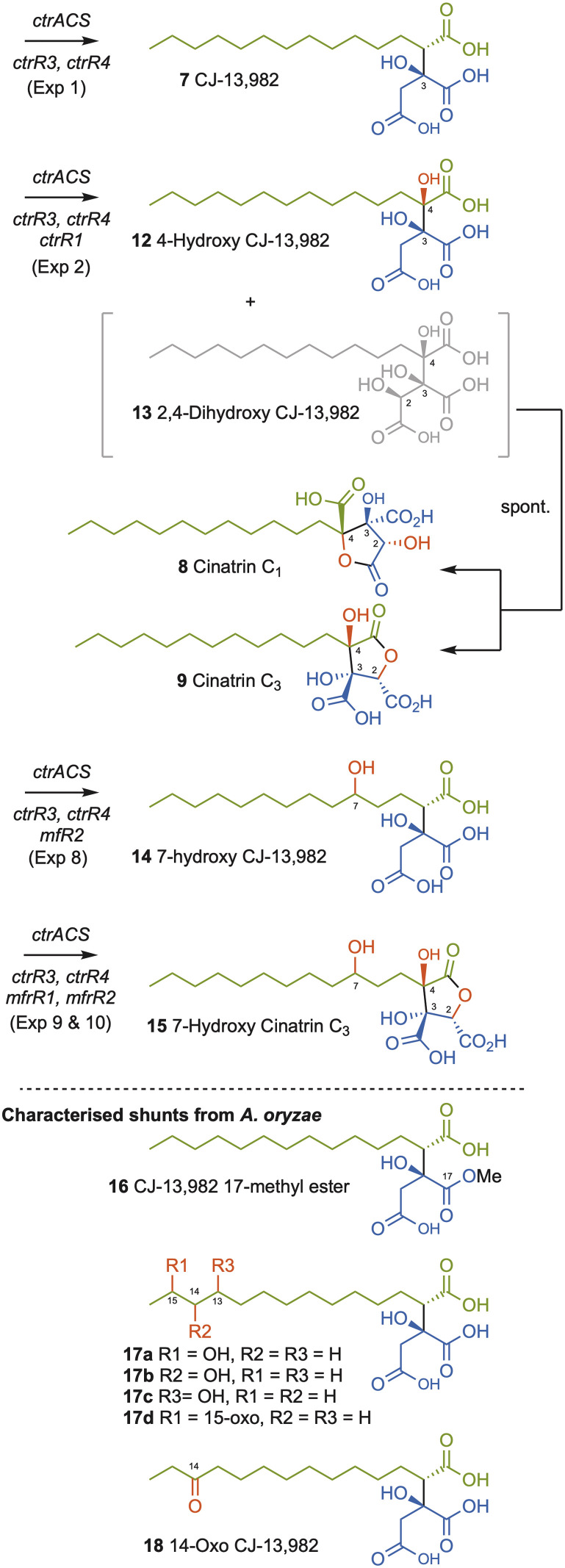
Synthesis of alkyl citrates by heterologous expression of *ctr* and *mf* genes in *A. oryzae* NSAR1 or reaction *in vitro*. Oxygenation positions shown in red.

Compound 7 was purified and identified by full NMR. Analysis of optical rotation, and comparison to literature data identified 7 as (2*S*, 3*S*)-CJ-13,982 (Scheme 2, SI Fig. S6–S12 and Tables S12–S13). A number of minor *A. oryzae* shunts were also purified and characterised. These were the 17-methyl ester of 7 (16, 0.5 mg L^−1^, SI Fig. S13–S18 and Table S14), an inseparable mixture of 13-, 14-, 15-hydroxy and 15-oxo-7 (17a–d, 3 mg L^−1^, SI Fig. S19–S25 and Tables S15–S19), and 14-oxo-7 (18, 0.9 mg L^−1^, SI Fig. S26–31 and Table S20).

We then coexpressed *ctrR1* encoding the putative NHI dioxygenase (Table 1, Exp2a, SI Fig. S32) and, in another experiment, both *ctrR1* and *ctrL2* encoding the putative FAD-dependent monooxygenase (Exp2b, SI Fig. S33). Transformants of Exp2a and Exp2b had identical chemotypes. CJ-13,982 7 was again produced (5.9 mg L^−1^ [from Exp2b]), but in addition 4-hydroxy CJ-13,982 12 (1 mg L^−1^ [from Exp2b], Fig. 1D, E, SI Fig. S34–S39 and Table S21) was purified and its structure elucidated. We also attempted purification of compound 13, but it isomerised to a mixture of cinatrin C_1_8 (<1 mg L^−1^, Scheme 2, SI Fig. S40–S45 and Tables S22–S23) and cinatrin C_3_9 (identified by comparison to literature data, 1.3 mg L^−1^ [from Exp2b], Scheme 2, SI Fig. S40–S45 and Tables S22–S23).^8,12,13^ Cinatrin C_1_8 and C_3_9 are known to be lactonised products of 13.^12^ The absolute stereochemistry for both cinatrins C_1_8 and C_3_9 is known to be 2*S*, 3*S*, 4*R*.^13^ The stereochemical relationships among these compounds suggest that 13 must therefore possess 2*S* stereochemistry.^26^

We further probed the potential function of the FMO encoded by *ctrL2* in different experimental setups *in vivo* and *in vitro*. However, we did not observe any new compounds in the presence of CtrL2, and thus the function of the FMO remains elusive.

To assess the possibility of using the NHI oxygenase CtrR1 *in vitro*, reactions with recombinant enzyme were conducted (Table 1, Exp3). CtrR1 was expressed in *E. coli* as a hexahistidine-tagged protein and purified by Ni-NTA chromatography (SI section 3 for details). Incubation of purified CtrR1 with CJ-13,982 7, FeSO_4_, α-ketoglutarate, and ascorbate at 28 °C for 2 h led to the synthesis of 12 and 13 with 8 and 9 detected as minor components. The addition of more enzyme over the course of the reaction resulted in the complete consumption of intermediate 4-hydroxy CJ-13,982 12 after 2 hours, forming 2,4-dihydroxy CJ-13,982 13 (SI Fig. S47).

Squalestatin S1 11 is an alkyl citrate derived from the condensation of a hexaketide with oxaloacetate that produces the key intermediate 10. Two NHI oxygenases MfR1 and MfR2 that are homologous to CtrR1 are known to be involved in the sequential oxygenation of 10 that ultimately leads to the 4,8-dioxabicyclo-[3.2.1]octane core of 11 that is a pM inhibitor of rat squalene synthase compared to the µM activity of 7.^6,11^ Since 7 is structurally analogous to 10 we were interested to test whether MfR1 and MfR2 could be used to convert 7 to a potentially more potent squalene synthase inhibitor. In previous studies, soluble MfR1 and MfR2 could not be produced in *E. coli*.^10^ For this reason, both enzymes were here expressed and purified as thioredoxin-fusion proteins^27^ (TrxA-MfR1 and TrxA-MfR2) that improves their solubility and activity *in vitro*. Incubation of purified TrxA-MfR1 with CJ-13,982 7, FeSO_4_, α-ketoglutarate, and ascorbate at 28 °C for 2 h resulted in the formation of 12 and 13 as observed for CtrR1 (Table 1, Exp4). However, *in vitro* reaction using TrxA-MfR2, FeSO_4_, α-ketoglutarate, and ascorbate at 28 °C (Exp5) resulted in synthesis of several new compounds that are consistent with the suggested oxidative modifications in the biosynthesis of 11. From a large scale *in vitro* reaction of 5 mg of substrate 7 with TrxA-Mfr2, compound 14 (0.6 mg) was obtained and fully characterized (Scheme 2, Table 1 Exp5, SI Fig. S24 and S52–S57).

Combined use of TrxA-MfR1 and TrxA-MfR2 with 7 as the substrate was also attempted (Table 1, Exp6). Several oxygenated products were observed by LCMS that are consistent with the oxidative pathway towards the 4,8-dioxabicyclo-[3.2.1]octane motif (see SI section 17 for details), but 14 remained the only isolated and fully characterised compound.

In order to produce further new compounds in a single process, heterologous expression in *A. oryzae* was again used. Four different gene combinations were created. Coexpression of *mfR1* with *ctrR4*, *ctrR3* and *ctrACS* (Table 1, Exp7) generated 7 as the major product together with small amounts of 4-hydroxy CJ-13,982 12 and 2,4-dihydroxy CJ-13,982 13 and the usual shunt compounds 16–18. Coexpression of *mfR2* with *ctrR4*, *ctrR3*, and *ctrACS* (Table 1, Exp8) resulted in production of 7 and 7-hydroxy CJ-13,982 14. In extracts of transformants containing *ctrR4*, *ctrR3*, *ctrACS* and both, *mfR1* and *mfR2* we identified 7 and 4-hydroxy CJ-13,982 12 (Table 1, Exp9), and a minor compound 15. To improve yields further, we additionally co-expressed *ctrR1* that appears to be a more effective 2,4-hydroxylase than MfR1 (Table 1, Exp10). This resulted in increased production of compound 15 (5.7 mg L^−1^) and allowed its identification as 7-hydroxy cinatrin C_3_ (Scheme 2, SI Fig. S63–S68 and Table S25).

Finally, the new compounds were tested as inhibitors of PLA_2_ and SQS. An *in vitro* SQS assay was established based on the consumption of NADPH.^28^*A. oryzae* SQS was expressed in *E. coli* and purified. The assay involved incubation of farnesyl diphosphate (FPP) and SQS with NADPH, and the consumption of NADPH was observed *via* fluorescence with an absorption wavelength of 360 nm and an emission wavelength of 460 nm in the presence and absence of inhibitors. Phospholipase A_2_ (PLA_2_) from porcine pancreas (Sigma-Aldrich) was assayed by measuring the rate of hydrolysis of 4-nitro-3-(octanoyloxy)benzoic acid (NOBA) and detecting the formation of 4-nitro-3-hydroxybenzoic acid (NHBA) spectrophotometrically at 425 nm in the presence and absence of inhibitors. Measured IC_50_ values of the compounds 6, 7, 8/9, 11, 14, and 15 are shown in Table 2 and raw data is included in the SI.

**Table 2:** IC_50_ values *vs.* SQS and PLA_2_ experimentally observed for alkyl citrates described in this study. n.t. = not tested

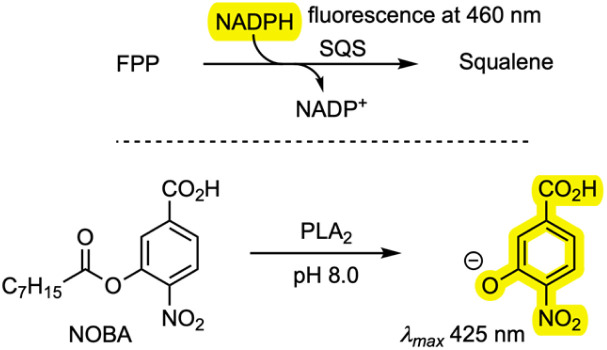			
Compound	IC_50_/µM *vs.*		PLA_2_/SQS Selectivity
	SQS	PLA_2_	
Sporothriolide 6	n.t.	> 1000.	—
CJ-13,982 7	33 ± 12^c^	293 ± 78	8.9
Cinatrin C_1_8 and C_3_9 mixture	18 ± 3.0	145 ± 10^b^	8.1
Squalestatin S1 11	0.092^a^	n.t.	—
7-Hydroxy-CJ-13982 14	24 ± 6	> 1000	> 41.6
7-Hydroxy-cinatrin C_3_15	57 ± 12	> 1000	> 17.5

a Lit. value 95.5 ± 13.6 nM *vs. T. elongatus* SQS.^29^

b Lit. value 70 µM *vs.* rat platelet PLA_2_.^7^

c Lit. value 1.1 µM *vs.* human SQS.^6^

## Conclusion

The biosynthesis of cinatrins has previously been suggested to follow the same pattern as the squalestatins.^16^ Here we showed that the *ctr* BGC, widely present in *Hypoxylon* species, encodes a pathway that does indeed follow the squalestatin model. Myristoyl CoA (C14 : 0) is likely to be the product of the FAS components (CtrR3 & CtrR4) and is condensed with oxaloacetate 2 by CtrACS to create CJ-13,982 7. CtrR1 then sequentially hydroxylates C-4 and then C-2 to create 13 that is the precursor to cinatrins C_1_8 and C_3_9.

CJ-13,982 7, cinatrins C_1_8 and C_3_9 and intermediate 12 are produced by total biosynthesis in *A. oryzae* in titers that allow isolation, full characterisation, and bio-testing after a single fermentation. This compares with 15-steps by total chemical synthesis.^16^ Furthermore, implementation of oxygenase steps from the squalestatin pathway allow the synthesis of new hybrid metabolites 14 and 15 for the first time. Thus, expression of the biosynthetic genes in *A. oryzae* provides a highly effective platform for the total biosynthesis of new and known alkyl citrates in a single step. None of the products of the *ctr* pathway have ever been reported as metabolites of the Hypoxylaceae, either from wild-collected material or from laboratory fermentations. Total biosynthesis is thus also an effective way to activate a silent and previously cryptic BGC.

The synthesis of 12 from 7 can also be achieved in a single step *in vitro* using purified CtrR1. Additionally, *in vitro* conversion of 7 by Mfr2 resulted in synthesis of new hybrid metabolite 14, again in yields sufficient for purification and characterisation. However, the *in vitro* synthesis is less convenient than total biosynthesis as it requires laborious purification of the biosynthetic proteins themselves and access to 7.

The total biosynthesis platform also offers a convenient way of screening oxygenases related to CtrR1. For example, we showed that MfR2 acts as a 7-hydroxylase, and MfR1 acts as a 2,4-dihydroxylase in this system.^30^ MfR2 appears to catalyse further oxygenations of the fatty-acid-derived chain, but a fully-developed 4,8-dioxabicyclo-[3.2.1]octane core could not be isolated. Never-the-less, new hybrid metabolites 14 and 15 were synthesised, characterised, and shown to inhibit SQS. Interestingly, the 7-hydroxylation improves selectivity for SQS *vs.* PLA_2_ (Table 2), but does not increase potency *vs.* SQS itself. Further oxidative modifications are clearly required to evolve the potency of 14 and 15 towards the nM potency of 11*vs.* SQS. It is hypothesised that biosynthetic gene clusters may evolve by gain or loss of biosynthetic genes.^31^ Our results support this idea because introduction of 7-hydroxylation by MfR2 alters the spectrum of enzyme inhibition of the pathway product and sets the scene for the eventual development of much more potent inhibition as observed for 11 itself.

In conclusion, our results show that total biosynthesis can be used as an effective platform for the synthesis of known and new specialised metabolites with bioactive properties. Total biosynthesis dramatically out-competes total chemical synthesis in terms of step-count. While total biosynthesis currently lacks the flexibility of total chemical synthesis, the ability to rationally combine biosynthetic genes from different pathways for the synthesis of new bioactive metabolites such as 14 and 15 illustrates that total biosynthesis offers a practical and effective route for the synthesis of known and new bioactive compounds. Since BGCs encoding very many alkyl citrates and related oxygenases^32^ are known it should now be possible to synthesise a wide range of related metabolites without the need for total chemical synthesis.

## Author contributions

H. H. conceptualization, investigation, supervision, writing – original draft; N. G. investigation, writing – review & editing; E. K. and J. G., informatics and visualization; B. W. and J. K., genome sequence, assembly and annotation; E. K., M. S., J. K., & R. J. C. funding acquisition, resources. R. J. C., supervision, writing – review & editing.

## Conflicts of interest

There are no conflicts to declare.

## Supplementary Material

SC-017-D6SC00660D-s001

## Data Availability

The data supporting this article have been included as part of the supplementary information (SI). Supplementary information: including NMR spectra, LCMS data and further experimental details. See DOI: https://doi.org/10.1039/d6sc00660d.
