# Supplementary material for: Total and engineered biosynthesis of cinatrins: inhibitors of phospholipase A2 and squalene synthase
Source: Chem Sci. 2026 May 8;17(25):12416–21. doi: 10.1039/d6sc00660d (PMC13178491; doi:10.1039/d6sc00660d)
Supplement: SC-017-D6SC00660D-s001 [file SC-017-D6SC00660D-s001.pdf]

Supplementary Information (SI) for Chemical Science.
This journal is © The Royal Society of Chemistry 2026

Total and Engineered Biosynthesis of Cinatrins: Inhibitors of Phospholipase A$_2$ and Squalene Synthase

Henrike Heinemann,[a] Nick Gerlach,[a] Eric Kuhnert,[a] Jennifer Gerke,[a] Bart Verwaaijen,[b] Jörn Kalinowski,[b] Marc Stadler[c] and Russell J. Cox[a*]

Electronic Supplementary Information

1. Sequencing and Informatics 3
1.1 Genome Sequencing and Annotation 3
1.2 Synteny Analysis of the *ctr* Biosynthetic Gene Cluster 3
1.3 Sequence Analysis of CtrR1 5
2. Microbiology and Vector Construction 6
2.1 General Procedures 6
2.2 Growth and Maintenance of Strains 6
2.3 Fungal gDNA and cDNA Preparation 6
2.4 Cloning Procedures 7
2.5 Transformation Protocols 9
2.6 Oligonucleotides 9
3. *In Vitro* Methods 13
3.1 Protein Production and Purification 13
3.2 Expressed Gene Sequences 14
3.3 In Vitro Preparation of Alkyl Citrates 16
3.4 Buffers 17
4. General Chemical Methods 18
4.1 Extraction of Secondary Metabolites 18
4.2 Protein Mass Spectrometry 18
4.3 High Resolution Mass Spectrometry 18
4.4 Liquid Chromatography Mass Spectrometry 18
4.5 Nuclear Magnetic Resonance 19
5. EXPERIMENT 1 (*ctrR4* + *ctrR3* + *ctrACS*) *in vivo* 20
5.1 Chacterisation of CJ-13,982, 7 21
5.2 Chacterisation of CJ-13,982 17-methyl ester, 16 26
5.3 Chacterisation of Inseparable Mixture of 13,14,15 hydroxy-CJ-13,982, 17a-c and 15-oxo-CJ-13,982 17d 31
5.4 Chacterisation of 14-oxo-CJ-13,982, 18 37
6. EXPERIMENT 2 (*ctrR4* + *ctrR3* + *ctrACS* + *ctrR1*) *in vivo* 42
6.1 Chacterisation of 4-Hydroxy CJ-13,982, 12 44
6.2 Characterization of Cinatrin C$_1$ (8) and Cinatrin C$_3$ (9) 49
7. EXPERIMENT 3 (7 + CtrR1) *in vitro* 55
8. EXPERIMENT 4 (7 + Mfr1) *in vitro* 57
9. EXPERIMENT 5 (7 + MfR2) *in vitro* 58
9.1 Characterization of 7-Hydroxy-CJ-13,982, 14 61
10. EXPERIMENT 6 (7 + MfR1 + Mfr2) *in vitro* 66
11. EXPERIMENT 7 (*ctrR4* + *ctrR3* + *ctrACS* + *mfr1*) *in vivo* 67
12. EXPERIMENT 8 (*ctrR4* + *ctrR3* + *ctrACS* + *mfr2*) *in vivo* 68
13. EXPERIMENT 9 (*ctrR4* + *ctrR3* + *ctrACS* + *mfr1* + *mfr2*) *in vivo* 69
14. EXPERIMENT 10 (*ctrR4* + *ctrR3* + *ctrACS* + *ctrR1* + *mfr2*) *in vivo* 70
14.1 Characterization of 7-Hydroxy-Cinatrin C$_3$, 15 71
15. Chemical Synthesis 76
15.1 Tris(tetrabutylammonium)hydrogenphosphate 19 76
15.2 Farnesylpyrophosphate 20 77
16. Bioactivity Assays 79

16.1 Squalene Synthase Assay ... 79
16.2 Phospholipase A_2 Assay ... 80
17. Comparison and Interpretation of CtrR1 and MfR1/2 Activity *in vitro* ... 81
17.1 Proposed Oxidative Pathway for *in vitro* Reactions of CJ-13,982 with Trx-MfR1 and Trx-MfR2 ... 81
17.2 Revision of Oxidative Steps during the Biosynthesis of Squalestatin S1 ... 82
18. Literature ... 85

1. Sequencing and Informatics

1.1 Genome Sequencing and Annotation

Sequencing of the *H. monticulosa* genome, its assembly and annotation, were described in Wibberg et al 2023.[1] Predictions of genes and their functions were made based on the funnannote pipeline.[2]

Table S1 Sequencing results for *H. monticulosa*

Strain	Genome size [bp]	Sequencing Method	Scaffolds/ contigs	N50 value	Annotated genes
H. monticulosa MUCL 54604	42769229	Illumina & Oxford Nanopore	28	3.44×10^{6}	12254

Genome sequence data for *H. monticulosa* MUCL 54604 is available at genbank: **GCA_902806465.1**
The annotated *ctr* BGC is uploaded to genbank: **PX666452**

1.2 Synteny Analysis of the *ctr* Biosynthetic Gene Cluster

The search for the *ctr* BGC was performed via a manual search strategy using the Blast algorithm: the ACS from *H. monticulosa* that was already identified in our previous study,[3] was used against the translated nucleotide database of the targeted organism with the software package Geneious v. 2022.1.1 using standard parameters (Max E-value 0.05, BLOSUM62 matrix, gap cost 11 1).[3, 4] The genomes contained at least three CS homologues with two of them being associated with primary metabolism. A fourth ACS was present in strains that are known producers of sporothriolide and part of the *spo* cluster.[5] Identified *ctr* BGCs were manually curated and the synteny analysis was conducted with clinker.[6]

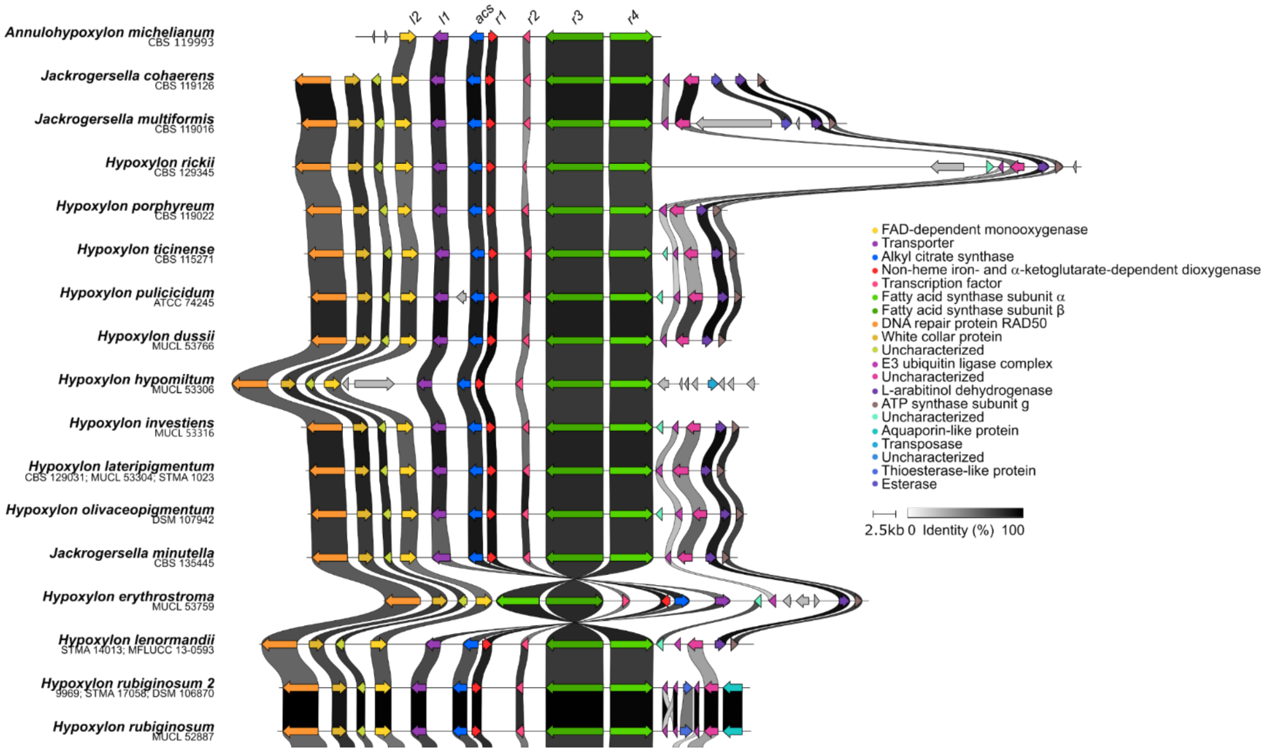

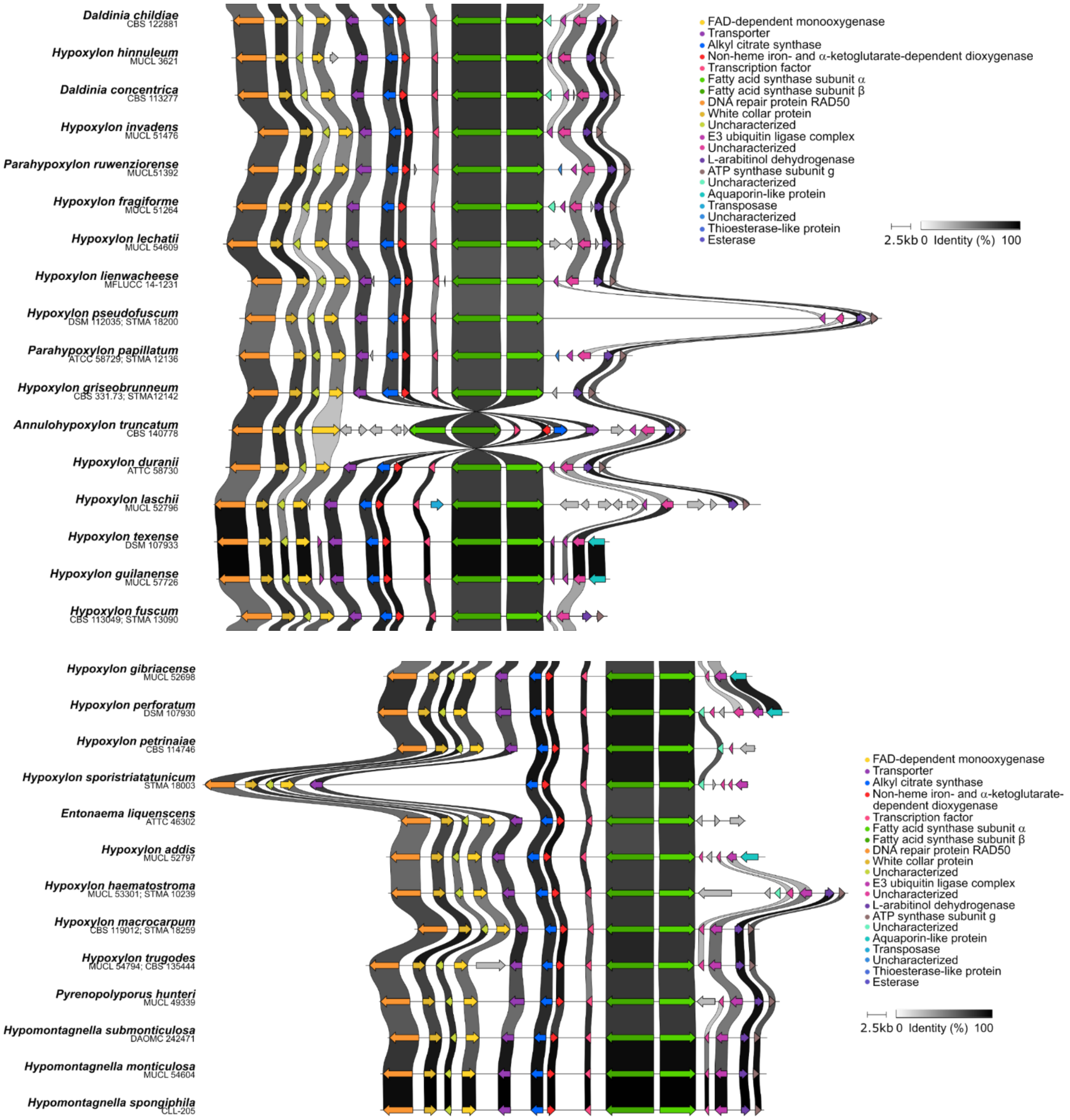

Figure S1 Synteny analysis of the identified *ctr* alkyl citrate biosynthetic gene cluster with homologous BGCs from other *Hypoxylon* species.

1.3 Sequence Analysis of CtrR1

The protein databank was searched for sequences similar to CtrR1. The best match was NvfI, an endoperoxide-forming enzyme from the novofumigatonin biosynthetic pathway in *Aspergillus novofumigatus*.[7,8] CtrR1, MfR1 and MfR2 were aligned with this sequence and residues known to be involved in catalysis were identified.

```
CtrR1  1    MAAADV-ATTTTGLIGMWGGTRDGATDGFIDWASGATNVHDPKQVKIEAEDMRTMNPVPSYMKDGYGIAKHKTSITPEQF  79
Mfr1   1    MATA-I-LPSTSGVIGLWDGTTDGK-EGFMDYANGDTNVKQPKEYEIQVHDIRKLDPQPTLLKNGYELVDIPTVVTDEQF  77
Mfr2   1    MATATTTLHSTTGTVYVADGTTDGK-VGYYNHTDDSTNVIR-KPIPIEVEDARTLSKSPTTKAEGYQLVNFHTKIPEEHF  78
NvfI   1    MVGSRTWCESEMLFVQPDAGTKEELYYRVTPKPGQTQANFNWTPHKVRFHDARPQRDSFDLNTHGFTFVEDAISPQLIER  80

CtrR1  80   L-AGNDPEGKKFLEDVYYKEVEELVKQITGS-SIVVPYVFRIRQNSMKAGEFMAAKLS-----HSALPVAHVDRDAITGE  152
Mfr1   78   IeSGKSDEGNAYIKDVYFAECKRIIEEVSGGvDLIIPVSFRMREQKGEK-ESTTKKLGNIESrYAPRPVAHLDRDTPTAI  156
Mfr2   79   L-NSKLPENKELIEEVYFDECRRLVQEVTGA-AEAYPYVYRVRNQEQNAKESNKSNFHTDF-----VPIVHVDRDDVTAP  151
NvfI   81   I----RADDTAAVEGDYFASVAALVKRVTGA-DHVVCFSPYTRKENSEK---------GIFG--QPARTVHCDHTPAAAI  144

CtrR1  153  DGVREVLG-EQADELIKQGKRYAQVNIWRSIDQPIKKWPLCFINHAGVEGWNYDSHLARVYPTNDPRIAIRGEKKHDSVV  231
Mfr1   157  TVLEETVGKEKAQELLSKHKRWAQVNVWRPIGNPATMWPLCFLNHDRIPTWNYDTHVGHVWSLNDPRVSDRGQKTYDCVV  236
Mfr2   152  QRLRASLGAEKADMLLSKYKSYGSINVWRPVKNMVQKWPLMLVDHKSIEDWDYSTHMFTLHSSNDERVATRGAKEHETIL  231
NvfI   145  ELTHKLCG-EDAVRLLQS--RFRAFSVWRPLVEPVLDWPLAVVDGRTIAPDDLHPVHWLRYEKKDTEPPFQ--------L  213

CtrR1  232  KYDPNYKYHYVSNLDIDEVLVFSSFDSD--VSKVVPHGAFWDDASPDDAPTRRSFEVRSWVFFD-  293
Mfr1   237  KHDDRYDYHYVSDLRPEECLVFCSFDSI--PKYAMPHSAFWDNNVPADAPNRRSIEVRSLVFF--  297
Mfr2   232  THDKRYRYIYASDMTPEEAWLFFAFHSD--PALGIPHGAFWDDSTKEEALTRCSIEVRIWVFFD-  293
NvfI   214  SFSETQKWYYLSRQRSDEVSIVKNYDSEvvPSPRSAHCAFKHPFVPKDAPPRESIDVRCLVFGGr  278
```

Figure S2 Protein sequence alignment of NHI oxygenases CtrR1, Mfr1, Mfr2 and NvfI using COBALT multiple sequence alignment tool.[9] Fe-binding sites are marked in yellow and α-KG-binding residues in green based on the position in the NvfI sequence. Endoperoxise NvfI from *Aspergillus novofumigatus* was included in the alignment as the closest CtrR1 homolog contained in the Swiss-Prot database and a well characterized non-heme iron - and α-ketoglutarate (α-KG)-dependent dioxygenase.

2. Microbiology and Vector Construction

2.1 General Procedures

Molecular biology kits were used according to manufacturer's protocols. Analytical PCR was carried out using OneTaq® DNA Polymerase (New England Biolabs), PCR for cloning was performed with Q5® High-Fidelity DNA Polymerase (New England Biolabs). Restriction endonucleases were purchased from New England Biolabs.

2.2 Growth and Maintenance of Strains

Escherichia coli OneShot™ Top10, *Escherichia coli* OneShot™ ccdB Survival™ 2 $T1^R$ were purchased from Thermo Fisher Scientific. *Saccharomyces cerevisiae* CEN.PK2 was purchased from Euroscarf. *Aspergillus oryzae* NSAR1 was provided by the Lazarus group.[10] *Hypomontagnella monticulosa* MUCL 54604 was obtained from the Stadler group in Braunschweig.[11]

2.2.1 ***Escherichia coli*** strains were cultivated on liquid or solid LB medium/ agar with appropriate antibiotics. Cultures were incubated at 37 °C for 12 to 18 h shaking at 200 rpm. For long term storage glycerol stocks (25 % - 50 % glycerol) were stored in cryo storage vials at -80 °C.

2.2.2 ***Saccharomyces cerevisiae*** strain CEN.PK2 was cultured on YPAD agar at 30 °C for 3-5 days. For liquid cultures a single colony was inoculated into YPAD medium and cultivated at 30 °C and 130 rpm for 18 h.

2.2.3 ***Aspergillus oryzae*** NSAR1 was grown on DPY agar at 28 °C for 5-7 days. Spores were suspended in H_2O; the suspension was mixed with glycerol to a final concentration of 50 % (*v/v*) and stored in cryo storage vials at -80 °C. Secondary metabolite production was achieved in liquid cultures. 500 mL baffled flasks containing 100 mL DPY medium were inoculated with 2 mL of a mycelia and spore suspension in H_2O from a 7 day old plate. The cultures were incubated at 28 °C and 110 rpm for 7 days.

2.2.4 ***Hypomontagnella monticulosa*** was grown on DPY-plates at 28 °C for 10-14 days. Stocks and genomic DNA was prepared from fresh mycelium, which was produced as follows: 100 mL DPY medium was inoculated with 1 mL of a spore suspension collected from a DPY plate and incubated at 28 °C for 5-7 days with shaking at 110 rpm. For storage, the cell suspension was mixed with glycerol to a final concentration of 50 % and stored in cryo storage vials at -80 °C.

2.3 Fungal gDNA and cDNA Preparation

A pea-sized portion of mycelia was dried with paper towels and incubated in 500 µL lysis buffer at 65 °C for 30 min. Next, it was incubated on ice for 5 min. 200 µL of 8 M KOAc were added, the mixture was mixed thoroughly and centrifuged for 15 min at 13.000 rpm. 300 µL of the liquid phase was added to 300 µL isopropanol in a new tube and the tube was inverted 8-10 times. After another centrifugation step for 3 min at 13,000 rpm the supernatant was removed. The pellet was washed with 1 mL 70 % high quality ethanol. After another centrifugation step for 3 min at 13,000 rpm the supernatant was removed and the pellet dried at 65 °C for ca. 10 min with an open lid. The genomic DNA was resuspended in 100 µL water and stored at 4 °C.

RNA extraction was performed with sterile consumables in a sterile clean bench. Fungal mycelia were collected by vacuum filtration. The cells were homogenized by mechanical disruption

using beads. RNA extraction from the homogenized mycelia was performed with Trizol™ (Thermo Fisher Scientific) following the manuals' instruction. RNA was optionally treated with DNase I (Zymo Research) according to the manufacturer's recommendations. Purified RNA was converted to cDNA immediately with RevertAid Premium Transcriptase kit (Thermo Fisher Scientific) and the Manufacturers' instructions were followed. For determination of gDNA contamination in the RNA, PCR amplifications of known intron-containing sequences were performed.

2.4 Cloning Procedures

2.4.1 Yeast Homologous Recombination. Transformation of *S. cerevisiae* CEN.PK2 was performed using a LiAc/SS-carrier DNA/PEG method.[12] 10 mL YPAD seed culture inoculated with a single colony was grown overnight for up to 18 h at 30 °C and 130 rpm. This seed culture was used to inoculate 40 mL YPAD medium (total volume was 50 mL after inoculation which was cultivated for 4-5 hours at the same conditions. Cells were harvested by centrifugation (3000 × g, 5 min, 4 °C) and the pellet was washed with 25 mL H_2O and resuspended in 1 mL H_2O in a 1.5 mL tube. The suspension was centrifuged again for 30 s at 11000 × g. The pellet was resuspended in 1 mL H_2O, and the mixture was divided into 100 µL aliquots. After another centrifugation step for 30 s at 11000 × g the supernatant was discarded, and the pellet resuspended in 360 µL transformation mixture. The transformation mixture contained 240 µL PEG 3350 (50 % [*w/v*]), 36 µL LiOAc (1 M), 50 µL carrier DNA (2 mg/mL salmon testis DNA) and 34 µL DNA mix. The DNA mix comprised pTYGS-*argB*/*adeA*/pE-YA (up to 2000 ng) linearized and fragmented with *Asc*I and PCR-derived genes of interest (in equimolar concentrations) that contained a 30 bp 3´ and 5´ overlap with the cut sites of the plasmid. Small DNA-'patches' were amplified from the intact pTYGS plasmid and used for recombination with integration sites which were not equipped with a gene of interest, so that the sequence at these sites was restored. The resulting mixture was incubated at 42 °C for 50 min, then centrifuged at 6000 × g for 1 min before the cell pellet was resuspended in 1 mL H_2O. 200 µL were streaked on SM-URA agar plates and incubated at 30 °C until colonies appeared.

2.4.2 Digest and Ligation. Restriction enzyme digests of plasmids or PCR products were performed as indicated in the manufacturer's guidelines of the enzymes (New England Biolabs or Thermo Scientific). The reaction mixtures were incubated at the recommended temperature for up to 12 h. Heat inactivation of the restriction enzymes was done by heating the samples at 65 °C or 80 °C for 20 min. Purification of the digested DNA or plasmid was done using the NucleoSpin® Gel and PCR Clean-up kit (Macherey-Nagel) as described previously. Self-ligation of digested plasmids was prevented by dephosphorylation using shrimp alkaline phosphatase (SAP). SAP was added after the enzymatic digest, and the mixture was incubated for 1 h at 37 °C. Ligation of the plasmids and inserts (molar ratio of 1 : 5) was done with T4 DNA ligase (New England Biolabs) following the manufacturer's instructions. After overnight incubation of the ligation reaction at 4 °C heat inactivation was performed for 10 min at 65 °C and plasmids were transformed to *E. coli* Top10.

2.4.3 Gateway® Technology. For *in vitro* recombination of fungal vectors the Gateway LR Clonase enzyme mix II kit (Invitrogen) was used according to the manufacturer´s instructions.

Table S2 Fungal vectors constructed by yeast homologous recombination.

Vector description	ONT for construction	Template
pE-YA·*ctrR3*	HH54 + HH55 (*ctrR3_1)*[a]<br>HH56 + HH57 (*ctrR3_2* [a]	*Hypomontagnella monticulosa* gDNA
pE-YA·*ctrL1*	HH168 + HH169 (*ctrL1)*[b]	*Hypomontagnella monticulosa* gDNA
pTYGS·*argB·ctrR4*	HH58 + HH59 (*ctrR4_1)*[b]<br>HH60 + HH61 (*ctrR4_2)*[b]	*Hypomontagnella monticulosa* gDNA
pTYGS·*ade·ctrACS·ctrR1·ctrL2*	HH62 + HH63 (*ctrACS)*[b]<br>HH64 + HH65 (*ctrR1)*[b]<br>HH66 + HH67 (*ctrL2)*[b]	*Hypomontagnella monticulosa* gDNA
pTYGS·*ade·ctrACS·ctrR1*	HH62 + HH63 (*ctrACS)*[b]<br>HH64 + HH65 (*ctrR1)*[b]	*Hypomontagnella monticulosa* gDNA
pTYGS·*ade·ctrACS*	HH62 + HH63 (*ctrACS)*[b]	*Hypomontagnella monticulosa* gDNA

[a] Annealing temperature was set to 55 °C in PCRs with OneTaq® and 57 °C for PCRs with Q5®.

[b] Annealing temperature was set to 58 °C in PCRs with OneTaq® and 60 °C for PCRs with Q5®.

Table S3 Fungal vectors constructed by Gateway® cloning.

Vector description	Entry vector	Destination vector
pTYGS·*argB·ctrR3·ctrR4*	pE-YA·*ctrR3*	pTYGS·*argB·ctrR4*

Table S4 Fungal expression vectors obtained.

Vector name	Vector description	Supplier/ Reference
KELI52A	pTYGS·*sC·mfr1*	ref 13
KELI52B	pTYGS·*sC·mfr2*	ref 13
KELI52C	pTYGS·*sC·mfr1·mfr2*	ref 13

Table S5 *E. coli* expression plasmids constructed in the project.

Vector name	Vector ID	ONT for construction	Template
pET28a(+)-CtrR1	pHHII04	HH128 + HH129	synthetic intron-free DNA
pETM22-TrxA-CtrR1	pHHII05	HH10 + HH02	synthetic intron-free DNA
pET28a(+)-Mfr1	pHHII08	HH170 + HH171	KEL152A
pETM22-TrxA-Mfr1	pHHII09	HH172 + HH171	KEL152A
pET28a(+)-Mfr2	pHHII10	HH173 + HH174	KEL152B
pETM22-TrxA-Mfr2	pHHII11	HH175 + HH174	KEL152B
pET28a(+)-SQSAo	pHHII12	pET28a_F + pET28a R	synthetic intron-free DNA

2.5 Transformation Protocols

2.5.1 ***Escherichia coli.*** Frozen chemically-competent *E. coli* cells were kept in the -80 °C freezer. Cells were thawed on ice and 1 µL of purified plasmid DNA or 10 µL of ligation mixture were added. The mixture was kept on ice for 30 min and subsequently the heat shock was performed for 45 seconds at 42 °C in the thermoblock. The cells were kept on ice for another 2 min before 100 µL of SOC medium was added. Afterwards, the cells were incubated at 37 °C for 45 min at 200 rpm. The cell suspension was spread on LB-agar plates treated with the correct antibiotic. Transformants were picked after overnight incubation at 37 °C.

2.5.2 ***Aspergillus oryzae*** **NSAR1.**[14] 50 mL GN-medium in a 250 mL baffled flask was inoculated with *A. oryzae* NSAR1 spores and mycelium from a DPY-plate and incubated at 28 °C and 110 rpm overnight for approx. 18 h. The mycelium was harvested by filtration with a miracloth filter, washed with approximately 50 mL 0.8 M NaCl and incubated in 10 mL sterilized protoplasting solution for 3-4 hours. The protoplasts were released from the hyphae by gentle pipetting with a wide bore pipette. The hyphae were removed by filtration through a miracloth filter. The filtrate containing the protoplasts was centrifuged at 3000 × g for 5 min at 4 °C and the visible pellet of protoplasts was resuspended in transformation solution 1 (100 µL per transformation). 500 - 4000 ng of prepared DNA (depending on the number of vectors used) was added to 100 µL protoplast suspension and incubated on ice for 2 min. Next, 1 mL of transformation solution 2 was added to the mixture and incubated at room temperature for 20 min. 5 mL of the respective selective soft-agar was added to the mixture and added to prepared plates with the respective selective agar. Plates were incubated at 28 °C for 4-6 days until colonies appeared. Colonies were picked and transferred to a fresh plate of selective agar. This was repeated two times before transformants were moved to DPY-plates.

2.6 Oligonucleotides

Table S6 Oligonucleotides used in this study.

ONT name	Sequence
T7 seq F	TAATACGACTCACTATAGGG
T7 seq R	CTAGTTATTGCTCAGCGGT
Peno plugF	CTTCTTAAATATCGTTGTAACTGTTCCTGA
Peno plugR	CGAAGTATATTGGGAGACTATAGCTACTAG
Padh plugF	ATTCACCACTATTATTCCCACCCTATAATA
Padh plugR	GAGACGAAACAGACTTTTTCATCGCTAAAA
PgdpA plugF	CTTTTCTTTTCTCTTTCTTTTCCCATCTTC
PgpdA plugR	TGACCTCCTAAAACCCCAGTG
M13 rev -29	CAGGAAACAGCTATGACC
M13 uni -21	TGTAAAACGACGGCCAGT

Table S7 Oligonucleotides designed in this study.

ONT name	Sequence
HH54	GCCAACTTTGTACAAAAAAGCAGGCTCCGCATGTTGGACATAGGAGACGG
HH55	GGAGTCCTTCTTGAACCATACTTCAAAGTCCTCATCC
HH56	GGATGAGGACTTTGAAGTATGGTTCAAGAAGGACTCC
HH57	TGCCAACTTTGTACAAGAAAGCTGGGTCGGTCACGACATCCCGATATTGC
HH58	TTCTTTCAACACAAGATCCCAAAGTCAAAGATGGCGATAGCACTTCAACG
HH59	CAGACATAAGTCCTATTATGAGTCGGCCCATCTCC
HH60	GGAGATGGGCCGACTCATAATAGGACTTATGTCTG
HH61	TTTCATTCTATGCGTTATGAACATGTTCCCCTAGAATCGCGCCGATGCGT
HH62	TTTCTTTCAACACAAGATCCCAAAGTCAAAATGGAACAATACCTCGAAGTC
HH63	TTCATTCTATGCGTTATGAACATGTTCCCTCTAGTTCGCTAGCGGCTTAAC
HH64	TAACAGCTACCCCGCTTGAGCAGACATCACATGGCTGCTGCTGATGTCGC
HH65	ACGACAATGTCCATATCATCAATCATGACCTTAGTCAAAGAAGACCCACGAGC
HH66	TCGACTGACCAATTCCGCAGCTCGTCAAAGATGGAATACGTCGACGTCTTG
HH67	AGGTTGGCTGGTAGACGTCATATAATCATATTAAGCCGAGAGCTTCCAAATC
HH108	CGGCAGCCATATGATGGAACAATACCTCGAAGTC

HH109	TGGTGGTGCTCGAGCTAGTTCGCTAGCGGCTTAAC
HH128	GGCAGCCATATGATGGCTGCTGCTGATGTC
HH129	CTCGAGTGCGGCCGCTTAGTCAAAGAAGACCCA
HH130	AGGGGCCCATGGATATGGCTGCTGCTGATGTC
HH170	GGCAGCCATATGATGGCGACAGCTATCCTTC
HH171	TCGAGTGCGGCCGCCTAAAAGAACACCAAGGAG
HH172	AGGGGCCCATGGATATGGCGACAGCTATCCTTC
HH173	GGCAGCCATATGATGGCGACTGCAACAACAAC
HH174	TCGAGTGCGGCCGCCTAGTCAAAGAAAACCCAG
HH175	AGGGGCCCATGGATATGGCGACTGCAACAACAAC
pET28a_F	CGGCAGCCATATGACCGAAACACAGAAAGCCTG
pET28a_R	CTCGAGTGCGGCCGCTTAGGCTTCTTGACGTGC

2.7 Media, Agar and Solutions

Table S8 Media and agar used in this study.

**Medium**	**Composition**
CZD/S1 agar	3.50 % (*w/v*) Czapek Dox broth (Duchefa Biochemie)<br>18.22 % (*w/v*) D-sorbitol (= 1 M) (Roth)<br>0.10 % (*w/v*) ammonium sulfate (Roth)<br>0.15 % (*w/v*) L-methionine (Roth)<br>1.50 % (*w/v*) agar (Duchefa Biochemie)
CZD/S1 soft agar	3.50 % (*w/v*) Czapek Dox broth (Duchefa Biochemie)<br>18.22 % (*w/v*) D-sorbitol (= 1 M) (Roth)<br>0.10 % (*w/v*) ammonium sulfate (Roth)<br>0.15 % (*w/v*) L-methionine (Roth)<br>0.80 % (*w/v*) agar (Duchefa Biochemie)
CZD/S1 agar without methionine	3.50 % (*w/v*) Czapek Dox broth (Duchefa Biochemie)<br>18.22 % (*w/v*) D-sorbitol (= 1 M) (Roth)<br>0.10 % (*w/v*) ammonium sulfate (Roth)<br>1.50 % (*w/v*) agar (Duchefa Biochemie)
CZD/S1 soft agar without methionine	3,50 % (*w/v*) Czapek Dox broth (Duchefa Biochemie)<br>18.22 % (*w/v*) D-sorbitol (= 1 M) (Roth)<br>0.10 % (*w/v*) ammonium sulfate (Roth)<br>0.80 % (*w/v*) agar (Duchefa Biochemie)
DPY agar	2.00 % (*w/v*) dextrin from potato starch (Sigma Aldrich)<br>1.00 % (*w/v*) polypeptone (Roth)<br>0.50 % (*w/v*) yeast extract (Duchefa Biochemie)<br>0.50 % (*w/v*) monopotassium phosphate (Roth)<br>0.05 % (*w/v*) magnesium sulfate hexahydrate (Sigma Aldrich)<br>2.50 % (*w/v*) agar (Duchefa Biochemie)
DPY medium	2.00 % (*w/v*) dextrin from potato starch (Sigma Aldrich)<br>1.00 % (*w/v*) polypeptone (Roth)<br>0.50 % (*w/v*) yeast extract (Duchefa Biochemie)<br>0.50 % (*w/v*) monopotassium phosphate (Roth)<br>0.05 % (*w/v*) magnesium sulfate hexahydrate (Sigma Aldrich)
GN medium	2.00 % (*w/v*) D(+)-glucose monohydrate (Roth)<br>1.00 % (*w/v*) nutrient broth Nr. 2 from Oxoid (Fisher Scientific)
LB agar	0.5 % (*w/v*) yeast extract (Duchefa Biochemie)<br>1 % (*w/v*) tryptone (Duchefa Biochemie)<br>0.5 % (*w/v*) sodium chloride (Roth or VWR)<br>1.5 % (*w/v*) agar (Duchefa Biochemie)
LB medium	0.5 % (*w/v*) yeast extract (Duchefa Biochemie)<br>1 % (*w/v*) tryptone (Duchefa Biochemie)<br>0.5 % (*w/v*) sodium chloride (Roth or VWR)
PDB medium	2,40 % (*w/v*) potato dextrose broth (Formedium)
SM-URA agar	0.17 % (*w/v*) yeast nitrogen base (Sigma Aldrich)<br>0.50 % (*w/v*) ammonium sulfate (Roth)<br>2.00 % (*w/v*) D(+)-glucose monohydrate (Roth)<br>0.077 % (*w/v*) complete supplement mixture minus uracil (Sigma Aldrich)<br>2.50 % (*w/v*) agar (Duchefa Biochemie)
SOC medium	93.75 mL SOB Medium<br>1.25 mL magnesium chloride hexahydrate (Roth) (2 M)<br>5 mL D(+)-glucose monohydrate (Roth) (20 %)<br>For 100 mL SOC
YPAD	1.00 % (*w/v*) yeast extract (Duchefa Biochemie)<br>2.00 % (*w/v*) tryptone (Duchefa Biochemie)<br>2.00 % (*w/v*) D(+)-glucose monohydrate (Roth)<br>0.03 % (*w/v*) adenine (Roth)

YPAD Agar	1.00 % (*w/v*) yeast extract (Duchefa Biochemie)<br>2.00 % (*w/v*) tryptone (Duchefa Biochemie)<br>2.00 % (*w/v*) D(+)-glucose monohydrate (Roth)<br>0.03 % (*w/v*) adenine (Roth)<br>1.50 % (*w/v*) agar (Duchefa Biochemie)

Table S9 Solutions and buffers used in this study.

Application	Buffer or solution	Composition
A.oryzae transformation	Solution 1	0.8 M sodium chloride<br>10 mM calcium chloride<br>50 mM Tris-HCl, pH 7.5
	Solution 2	60 % (*w/v*) PEG 3350<br>0.8 M sodium chloride<br>10 mM calcium chloride<br>50 mM Tris-HCl, pH 7.5
	Protoplasting solution	10 mg/ mL lysing enzymes from *Trichoderma harzi-anum* (Sigma Aldrich) in 0.8 M NaCl<br>or<br>VinoTaste® Pro (Lamothe-Abiet) in 0.8 M NaCl
Fungal genomic DNA extraction	Lysis buffer	Lysis Buffer (100 mL)<br>5 mL 1M Tris (pH 7.2)<br>10 mL 0.5 M EDTA<br>3 g SDS<br>1 mL mercaptoethanol
	KOAc solution	8 M potassium acetate
Agarose gel electrophoresis	50x TAE buffer	2 M Tris acetate pH 8.3<br>0.05 M EDTA

3. *In Vitro* Methods

3.1 Protein Production and Purification

3.1.1 *E. coli* Culture Conditions. Seed cultures with 2-3 colonies of *E. coli* BL21 containing the required plasmid were grown overnight at 30 °C in 5-10 mL LB medium containing the appropriate antibiotics. 1 L LB medium containing the appropriate antibiotics were inoculated with 5 mL seed culture. Cultures were incubated at 37 °C. OD_{600} was monitored, and temperature was reduced to 18 °C at an OD_{600} of 0.25-0.30. Cells were induced with 100 µM isopropyl-β-D-thiogalactopyranoside (IPTG) at an OD_{600} of 0.6.

Cells were harvested after ~ 15-19 h of induction period. A 10 minute centrifugation step was performed at 4 °C and 6000 g. Cell pellets were transferred to fresh 50 mL tubes. At this point pellets could be stored in the -80 °C fridge. For further purification process 20 mL appropriate lysis buffer were added to tubes containing the cell pellet of 1 L cell culture.

Sonication was performed using SonoPlus Ultrasonic homogenizer from Bandelin with the following settings: Amplitude: 34 %. Sonication was done for 7 min. The cells were intermittently sonicated on ice for 10 s with 10 s allowed for cooling. The cell lysate was cleared by centrifugation at 20000 rpm for 40 min at 4 °C. The obtained supernatant was used in further purification steps. Samples of the supernatant and the pellet were used for SDS-PAGE.

3.1.2 Ni-NTA Affinity Chromatography. For manual Ni-NTA affinity chromatography 2 mL resin in ethanol (Ni-NTA agarose (cube biotech)) per liter of culture were washed 3 times with 2 mL of the respective lysis buffer per liter of culture. After washing, 2 mL lysis buffer was added to the pure resin. The appropriate amount of cell lysate was added to tubes containing the resin and tubes were placed at a rocker at 4 °C for 1 h. A 10 mL syringe was prepared with a PE-filter (CHROMABOND®) and the suspension was added to the syringe after the incubation time. Once the lysate ran through (collected as 'FT' in a tube underneath the column), 10 mL of lysis buffer were placed on the column. Remaining resin in the used tubes was additionally added to the column by washing the tubes using the buffer. Once this liquid ran through (collected as 'LB'), 10-15 mL of the wash buffer were placed on the column. After this ran through (collected as 'WB'), 7-10 mL of elution buffer 1 were added to the column and the running through liquid was collected as 'E1). Next, 2 mL of elution buffer 2 were added and the flowthough was collected as E2. Otherwise Ni-NTA affinity chromatography was performed with the ÄKTA™ pure system and a HisTrap™ column (GE Healthcare; column volumes (CV) = 5 mL) following the manufacturer's instructions enabling for a linear gradient. Filtered and degassed lysis and elution buffer 2 were used.

Elutions were either kept at 4 °C overnight or further processed on the same day. Before storage at -80 °C or -20 °C, proteins were concentrated to a concentration of approximately 15 mg/mL (quantified with a DeNovix® DS-11+ Spectrophotometer, calculation of protein molecular weight and extinction coefficients was done using Benchling) and protein concentration was done with Amicon Ultra centrifugal filters from Millipore.

3.1.3 SDS-PAGE. Samples for analysis in SDS-PAGE were taken immediately after the corresponding fraction was generated and 4 : 1 diluted in 4 × Lämmli buffer, except for lysate and flow through samples which were 1 : 10 diluted in water before being diluted 4 : 1 in 4 × Lämmli buffer. 15 µL of each sample were added to the pockets of the gel and 4 µL of the ladder (Color Prestained Protein Standard, Broad Range (10 - 250 kDa) or Color Prestained Protein Standard, Broad Range (11-245 kDa)) were used. Electrophoresis was done using BioRad gel electrophorese systems and gels with 12 % polyacrylamide separating and 5 % stacking gel layers at 50 mA for 45 min and 1 × SDS running buffer was used as buffer.

Gels were incubated in 30 mL Coomassie staining solution for 30 min or overnight. Destaining was done using Coomassie bleach solution for 30 min or overnight. Gels were scanned using the Molecular Imager Gel doc XR+ (Bio-Rad) system and digital images of the gels were created with the associated software or with mobile phone cameras.

3.2 Expressed Gene Sequences

Table S10 Gene sequences of expressed proteins. 6x-His Tag used for purification is labelled in bold letters.

Gene name	Sequence
SQSAo	ATGGGCAGCAGC**CATCATCATCATCATCAC**AGCAGCGGCCTGGTGCCGCGCGGCAGCCATATGACCGA<br>AACACAGAAAGCCTGCTTTAAATTCCTGGATCTGACCAGCCGTAGCTTTAGCGCAGTTATTAAAGAACTG<br>CATCCGGAACTGCTGCTGCCGGTTTGTGTTTTTATCTGGTTCTGCGTGGTCTGGATACCATTGAAGATG<br>ATACCAGCATTCCGCTGAAAACCAAAGAACCGATGCTGCGTGAATTCAAAGATTATCTGGAACAGGATGG<br>TTGGACCTTTGATGGTAATCGTCCGGAAGAAAAAGATCGTGAACTGCTGGTTCAGTTTCATAACGTGATTA<br>CCGAGTTTAAAAACATGAAACCTGCCTATCGCGAAATCGTGAAAGATATCACCGATAAAATGGGTAATGG<br>CATGGCAGATTATTGTCGCAAAGCAGAATTTGAAGATGCCAGCGTTAAAACCATCGAAGAGTATGATCTG<br>TATTGCTATTATGTTGCAGGTCTGGTTGGTGAAGGTCTGACCCGTCTGTTTGTTGAAGCCGAATTTGGTAA<br>TCCTGCACTGCTGAGCCGTCCGCGTCTGCATAAAAGCATGGGTCTGTTTCTGCAAAAAACCAACATTATT<br>CGTGATGTGCGCGAAGATCATGATGATGATCGTCATTTTTGGCCGAAAGAAATTTGGAGCAAATATGTGA<br>CCGAATTCGAGGACCTGTTTAAACCGGAAAATCGCGAAACCGCACTGAATTGTGGTAGCGAAATGGTTCT<br>GAATGCACTGGAACATGCAGAAGAATGTCTGTTCTATCTGGCAGGTCTGCGTGAACAGAGCGTTTTTAAC<br>TTTTGTGCAATTCCGCAGGCAATGGCAATTGCAACCCTGGAACTGTGTTTTCGTAATCCGGATATGTTTGA<br>CCGCAACATCAAAATCACCAAAGGTGAAGCATGTCAGCTGATGATGGAAAGCACCCAGAATCTGCATGTG<br>CTGTGTGATACCTTTCGTCGTTATGCACGTCGTATCCATAAAAAGAATACCCCGAAAGATCCGAACTTCCT<br>GAAAATTAGCATTGTGTGCGGCAAAATCGAGAAATTCATCGATACCATTTTTCCGCAGCAGACCGCAGCA<br>CAGGCAAAACTGAAAGTTCAGGGTGAAAAAAGCGAAGCCGAAAAAGAAAAAGCACGTCAAGAAGCCTAA
ctrR1	ATGGGCAGCAGC**CATCATCATCATCATCAC**AGCAGCGGCCTGGTGCCGCGCGGCAGCCATATGATGGC<br>TGCTGCTGATGTCGCTACTACCACCACTGGCCTCATTGGCATGTGGGGCGGTACCCGGGACGGTGCTAC<br>CGATGGATTCATTGACTGGGCCAGCGGTGCTACCAATGTCCACGACCCCAAGCAGGTTAAGATTGAGGC<br>CGAGGACATGCGAACCATGAACCCCGTCCCTTCCTACATGAAGGATGGATACGGCATTGCCAAGCACAA<br>GACCTCTATCACCCCCGAGCAGTTCCTGGCCGGCAACGACCCCGAAGGCAAGAAGTTCCTCGAGGATGT<br>CTACTACAAGGAGGTTGAGGAGCTGGTCAAGCAGATCACCGGCTCCAGCATCGTAGTCCCCTACGTGTT<br>CCGCATCCGCCAGAACAGCATGAAGGCGGGCGAGTTCATGGCCGCGAAGCTCTCTCACTCCGCTCTTCC<br>CGTCGCCCACGTTGACCGTGACGCGATAACGGGCGAGGACGGTGTCCGCGAGGTGCTCGGCGAGCAG<br>GCCGACGAGCTCATCAAGCAGGGCAAGCGCTACGCCCAGGTCAACATCTGGCGCAGCATCGACCAGCC<br>CATCAAGAAGTGGCCCCTCTGCTTCATCAACCACGCCGGCGTCGAGGGCTGGAACTACGACTCCCACCT<br>CGCCCGCGTCTACCCCACCAACGACCCCCGCATTGCCATCCGAGGCGAGAAGAAGCACGACAGCGTCG<br>TCAAGTACGACCCTAACTACAAGTACCACTACGTCAGCAACCTCGACATCGACGAGGTCCTCGTCTTCTC<br>GTCGTTCGACTCGGATGTGTCTAAGGTGGTTCCCCACGGTGCTTTCTGGGATGATGCCAGCCCTGACGA<br>TGCGCCCACCCGACGATCCTTCGAGGTTCGCTCGTGGGTCTTCTTTGACTAA
ctrR1 (with TrxA-tag)	ATGAGCGATAAAATTATTCACCTGACTGACGACAGTTTTGACACGGATGTACTCAAAGCGGACGGGGCGA<br>TCCTCGTCGATTTCTGGGCAGAGTGGTGCGGTCCGTGCAAAATGATCGCCCCGATTCTGGATGAAATCG<br>CTGACGAATATCAGGGCAAACTGACCGTTGCAAAACTGAACATCGATCAAAACCCTGGCACTGCGCCGA<br>AATATGGCATCCGTGGTATCCCGACTCTGCTGCTGTTCAAAAACGGTGAAGTGGCGGCAACCAAAGTGG<br>GTGCACTGTCTAAAGGTCAGTTGAAAGAGTTCCTCGACGCTAACCTGGCCGGTTCTGGTTCTGGCCATAT<br>G**CACCATCATCATCATCAT**TCTTCTGGTCTGGAAGTTCTGTTCCAGGGGCCCATGGATATGGCTGCTGCT<br>GATGTCGCTACTACCACCACTGGCCTCATTGGCATGTGGGGCGGTACCCGGGACGGTGCTACCGATGG<br>ATTCATTGACTGGGCCAGCGGTGCTACCAATGTCCACGACCCCAAGCAGGTTAAGATTGAGGCCGAGGA<br>CATGCGAACCATGAACCCCGTCCCTTCCTACATGAAGGATGGATACGGCATTGCCAAGCACAAGACCTCT<br>ATCACCCCCGAGCAGTTCCTGGCCGGCAACGACCCCGAAGGCAAGAAGTTCCTCGAGGATGTCTACTAC<br>AAGGAGGTTGAGGAGCTGGTCAAGCAGATCACCGGCTCCAGCATCGTAGTCCCCTACGTGTTCCGCATC<br>CGCCAGAACAGCATGAAGGCGGGCGAGTTCATGGCCGCGAAGCTCTCTCACTCCGCTCTTCCCGTCGC<br>CCACGTTGACCGTGACGCGATAACGGGCGAGGACGGTGTCCGCGAGGTGCTCGGCGAGCAGGCCGAC<br>GAGCTCATCAAGCAGGGCAAGCGCTACGCCCAGGTCAACATCTGGCGCAGCATCGACCAGCCCATCAA<br>GAAGTGGCCCCTCTGCTTCATCAACCACGCCGGCGTCGAGGGCTGGAACTACGACTCCCACCTCGCCC<br>GCGTCTACCCCACCAACGACCCCCGCATTGCCATCCGAGGCGAGAAGAAGCACGACAGCGTCGTCAAG<br>TACGACCCTAACTACAAGTACCACTACGTCAGCAACCTCGACATCGACGAGGTCCTCGTCTTCTCGTCGT<br>TCGACTCGGATGTGTCTAAGGTGGTTCCCCACGGTGCTTTCTGGGATGATGCCAGCCCTGACGATGCGC<br>CCACCCGACGATCCTTCGAGGTTCGCTCGTGGGTCTTCTTTGACTAA
mfr1 (with TrxA-tag)	ATGAGCGATAAAATTATTCACCTGACTGACGACAGTTTTGACACGGATGTACTCAAAGCGGACGGGGCGA<br>TCCTCGTCGATTTCTGGGCAGAGTGGTGCGGTCCGTGCAAAATGATCGCCCCGATTCTGGATGAAATCG<br>CTGACGAATATCAGGGCAAACTGACCGTTGCAAAACTGAACATCGATCAAAACCCTGGCACTGCGCCGA<br>AATATGGCATCCGTGGTATCCCGACTCTGCTGCTGTTCAAAAACGGTGAAGTGGCGGCAACCAAAGTGG<br>GTGCACTGTCTAAAGGTCAGTTGAAAGAGTTCCTCGACGCTAACCTGGCCGGTTCTGGTTCTGGCCATAT<br>G**CACCATCATCATCATCAT**TCTTCTGGTCTGGAAGTTCTGTTCCAGGGGCCCATGGATATGGCGACAGC<br>TATCCTTCCTTCGACATCTGGCGTGATTGGGCTTTGGGATGGCACGACAGATGGTAAAGAGGGGTTCAT<br>GGATTATGCAAATGGTGATACGAACGTGAAACAGCCAAAGGAGTACGAGATTCAAGTCCACGACATTCGA<br>AAGCTTGATCCTCAGCCTACTTTGCTTAAGAACGGGTACGAGCTCGTGGATATTCCTACGGTAGTGACCG<br>ATGAGCAATTTATTGAGTCTGGGAAGAGCGATGAAGGCAACGCGTACATCAAAGATGTCTACTTCGCGGA<br>GTGCAAAAGGATCATAGAGGAGGTCTCAGGGGGAGTTGATCTTATCATTCCTGTTTCCTTCCGTATGCGT<br>GAACAGAAGGGCGAGAAAGAGAGCACTACCAAGAAGCTCGGCAACATAGAGTCTAGATATGCTCCGCGT<br>CCCGTGGCGCATCTTGACCGAGACACCCCGACTGCCATCACGGTTCTAGAGGAGACTGTGGGCAAGGA<br>GAAGGCGCAGGAGCTGCTCTCGAAACACAAGCGCTGGGCCCAGGTGAATGTATGGAGACCCATAGGAA<br>ACCCCGCAACGATGTGGCCGCTTTGCTTCCTGAATCATGATCGCATTCCCACGTGGAACTACGATACCCA<br>CGTTGGCCATGTCTGGTCTTTGAACGATCCCAGAGTTTCGGACCGCGGACAGAAGACCTATGACTGCGT<br>TGTCAAACACGATGATCGATATGACTACCACTACGTCAGTGATCTTAGGCCAGAGGAATGCCTTGTCTTC<br>TGCTCTTTTGACTCGATCCCGAAATACGCTATGCCCCATAGCGCTTTCTGGGACAACAATGTCCCCGCTG<br>ATGCCCCGAATCGCCGTTCCATCGAGGTTCGCTCCTTGGTGTTCTTTTAG

mfr2 (with TrxA-tag)	ATGAGCGATAAAATTATTCACCTGACTGACGACAGTTTTGACACGGATGTACTCAAAGCGGACGGGGCGA<br>TCCTCGTCGATTTCTGGGCAGAGTGGTGCGGTCCGTGCAAAATGATCGCCCCGATTCTGGATGAAATCG<br>CTGACGAATATCAGGGCAAACTGACCGTTGCAAAACTGAACATCGATCAAAACCCTGGCACTGCGCCGA<br>AATATGGCATCCGTGGTATCCCGACTCTGCTGCTGTTCAAAAACGGTGAAGTGGCGGCAACCAAAGTGG<br>GTGCACTGTCTAAAGGTCAGTTGAAAGAGTTCCTCGACGCTAACCTGGCCGGTTCTGGTTCTGGCCATAT<br>G**CACCATCATCATCATCAT**TCTTCTGGTCTGGAAGTTCTGTTCCAGGGGCCCATGGATATGGCGACTGC<br>AACAACAACTCTACACTCCACAACTGGAACCGTCTACGTCGCTGATGGTACCACAGACGGCAAAGTAGGA<br>TATTACAACCACACGGATGATTCCACCAACGTCATAAGGAAGCCAATCCCGATTGAAGTCGAAGATGCAC<br>GCACGCTAAGCAAGAGCCCAACGACAAAGGCAGAAGGCTATCAGCTAGTCAATTTCCACACTAAGATTCC<br>AGAGGAGCACTTTTTGAATTCGAAACTTCCGGAGAACAAGGAACTCATCGAAGAGGTGTACTTCGACGAA<br>TGCCGTCGCCTGGTTCAAGAAGTCACCGGTGCAGCAGAAGCCTACCCGTACGTGTATCGCGTCAGGAAC<br>CAAGAGCAAAACGCCAAAGAATCCAATAAATCCAACTTCCACACCGACTTTGTCCCGATCGTGCACGTTG<br>ATCGCGACGATGTCACTGCACCCCAAAGACTGCGGGCCTCGCTTGGCGCGGAGAAAGCCGACATGCTA<br>TTGAGCAAATACAAGAGCTACGGATCGATCAATGTGTGGCGTCCAGTCAAGAACATGGTGCAGAAGTGG<br>CCTCTGATGCTCGTAGACCACAAGTCTATTGAGGATTGGGATTACAGCACGCACATGTTCACCTTGCATT<br>CTAGCAACGACGAAAGAGTTGCAACTCGTGGGGCGAAAGAGCATGAAACGATCCTCACGCATGATAAGA<br>GATATCGATACATCTATGCGTCCGATATGACGCCGGAAGAAGCTTGGCTGTTCTTTGCTTTTCACTCGGA<br>CCCGGCGTTGGGGATACCACATGGGGCTTTCTGGGATGATTCCACGAAGGAAGAAGCTTTGACGAGATG<br>CTCTATTGAAGTGCGGATCTGGGTTTTCTTTGACTAG

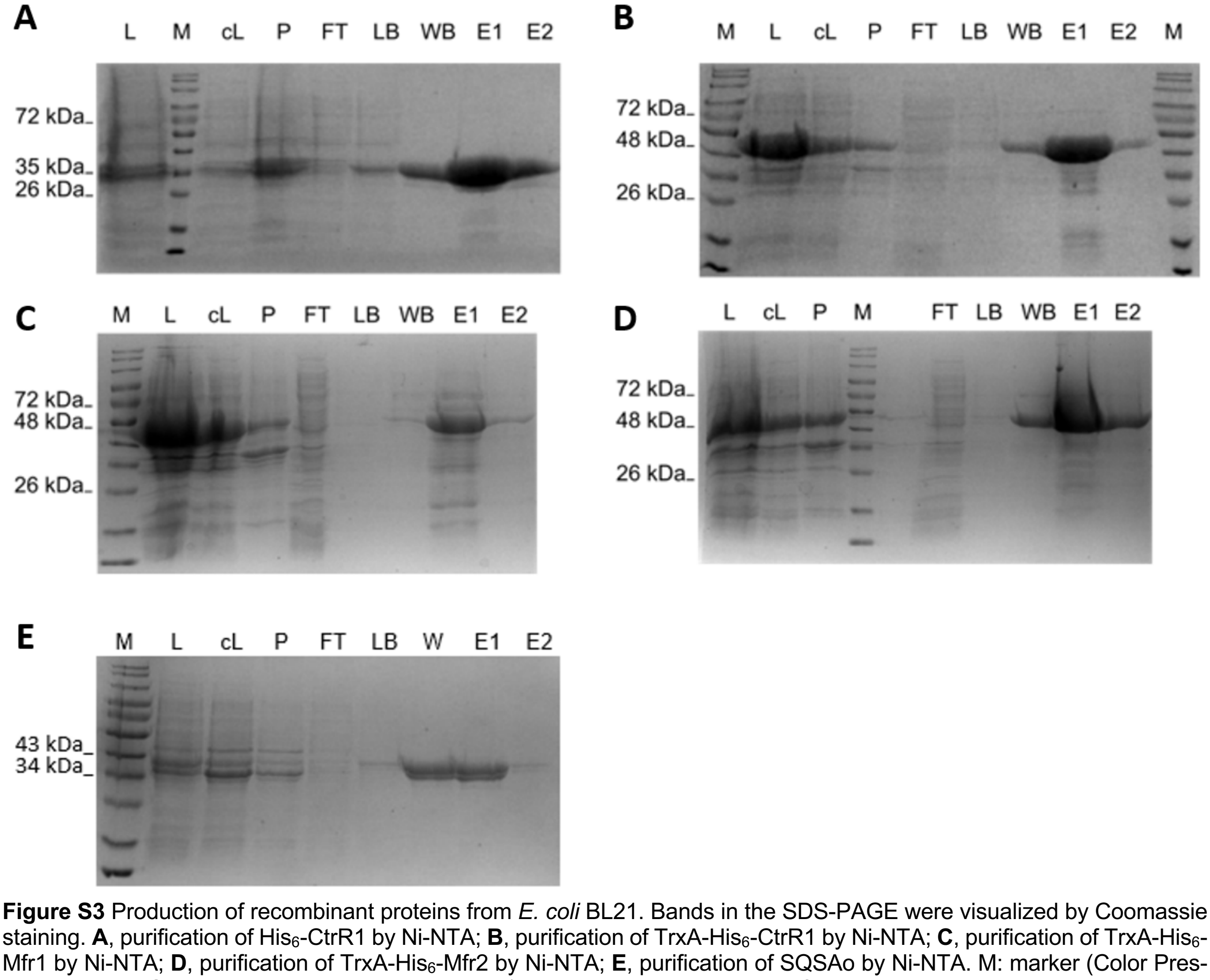

Figure S3 Production of recombinant proteins from *E. coli* BL21. Bands in the SDS-PAGE were visualized by Coomassie staining. **A**, purification of His_6-CtrR1 by Ni-NTA; **B**, purification of TrxA-His_6-CtrR1 by Ni-NTA; **C**, purification of TrxA-His_6-Mfr1 by Ni-NTA; **D**, purification of TrxA-His_6-Mfr2 by Ni-NTA; **E**, purification of SQSAo by Ni-NTA. M: marker (Color Prestained Protein Standard, Broad Range,10-250 kDa) L: protein sample prepared from lysate, cL: protein sample prepared from cleared lysate, P: protein sample prepared from insoluble pelleted fraction, FT: flow-through from Ni-NTA chromatography, LB: protein sample of wash with lysis buffer, WB: protein sample of wash with wash buffer (50 mM imidazole; 25 mM for TrxA-His_6-Mfr1 and TrxA-His_6-Mfr2), E1: protein sample of elution 1 (150 mM imidazol), E2: protein sample of elution 2 (500 mM imidazol).

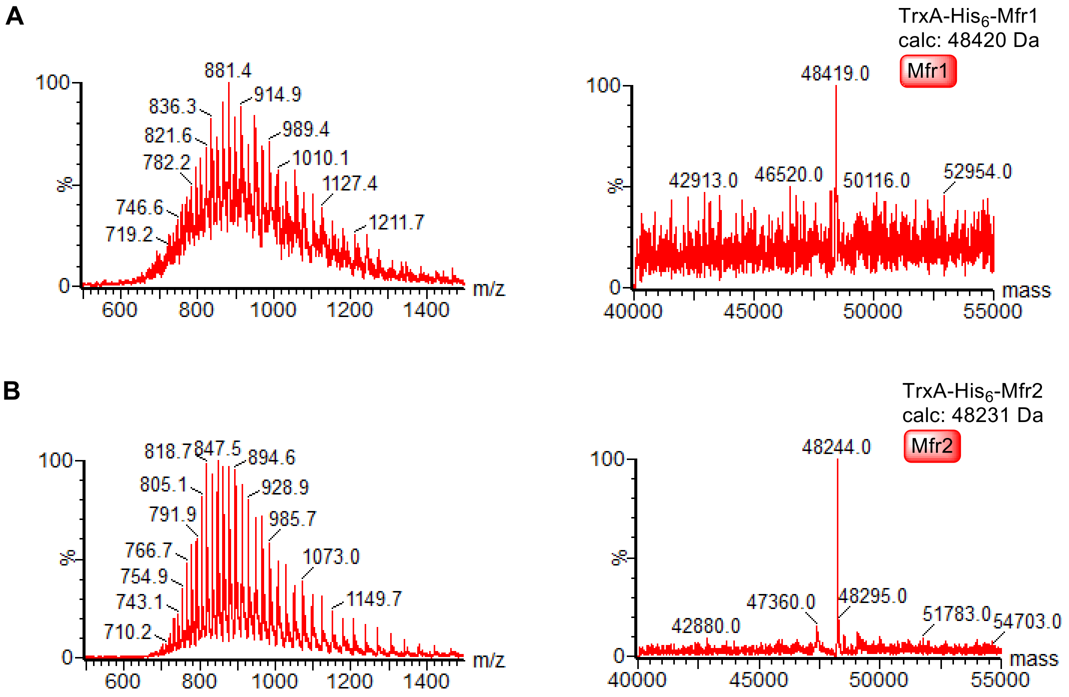

Figure S4 Mass spectrometry of TrxA-His$_6$-Mfr1 and TrxA-His$_6$-Mfr2 in H_2O at a concentration of 1 mg/mL. **A**, mass spectrum of TrxA-His$_6$-Mfr1 and zero-charge spectrum deconvoluted by MaxEnt; **B**, mass spectrum of TrxA-His$_6$-Mfr2 and zero-charge spectrum deconvoluted by MaxEnt.

3.3 In Vitro Preparation of Alkyl Citrates

3.3.1 Analytical *In Vitro* Preparation of 8, 9, 12, 13 and 14. (TrxA-)His$_6$-tagged NHI oxygenases were incubated at a concentration of 10 µM in a buffer containing 50 mM Tris, 0.1 mM $FeSO_4$, 8 mM ascorbate, 5 mM α-ketoglutarate and 2.5 mM CJ-13,982 **7** (stock solution diluted in DMSO) for 2 h at 28 °C. Optionally, 75 % of the amount of the initially added NHI oxygenase solution were added sequentially every 20 min over the course of the reaction. After 2 h of reaction time, one volume of acetonitrile was added to the reaction, the mixture was vortexed, the proteins precipitated by centrifugation and clear supernatant was analyzed by LCMS.

3.3.2 Preparative *In Vitro* Synthesis of 7-Hydroxy-CJ-13,982 14. A volume of 5 mL reaction buffer containing 50 mM Tris, 0.1 mM $FeSO_4$, 8 mM ascorbate, 5 mM α-ketoglutarate and 2.75 mM CJ-13,982 **7** (stock solution diluted in DMSO) and 10 µM TrxA-His$_6$-Mfr2 was incubated for 36 hours. The reaction mixture was stirred at 28 °C for 36 h and 75 % of the amount of the initially added NHI oxygenase solution were added sequentially every hour during day-time (8am - 8pm) to the mixture. After the 36 h the mixture was kept at RT for 12 more days without shaking. 0.6 mg (12 %) of **12** were isolated from the reaction mixture by preparative LCMS using a linear gradient from 40 % to 90 % CH_3CN.

3.4 Buffers

Table S11 Buffers and solutions used in this study.

Application	Buffer or solution	Composition
Protein purification<br>Tris buffer system<br>(TrxA-Mfr1, TrxA- Mfr2, CtrR1, TrxA-CtrR1)	Lysis buffer	50 mM Tris/HCl, pH 7.5<br>150 mM NaCl<br>10 mM imidazole<br>10 % (*v/v*) glycerol
	Wash Buffer	0.2 mL/ 0.5 mL/ 1 mL elution buffer 2<br>ad 10 mL lysis buffer
	Elution buffer 1	3.5 mL lysis buffer<br>1.5 mL elution buffer 2
	Elution buffer 2	50 mM Tris/HCl, pH 7.5<br>150 mM NaCl<br>500 mM imidazole<br>10 % (*v/v*) glycerol
	Storage buffer	50 mM Tris/HCl, pH 7.5<br>150 mM NaCL<br>10 % (*v/v*) glycerol
SDS PAGE	10x SDS buffer	25 mM Tris-HCl, pH 8.3<br>192 mM glycine<br>0.1 % (*w/v*) SDS
	Coomassie dye	25 % (*v/v*) acetic acid<br>10 % (*v/v*) isopropanol<br>0.1 % (*w/v*) coomassie
	Coomassie bleach	25 % (*v/v*) acetic acid<br>10 % (*v/v*) isopropanol
	4x Lämmli	10 % (*v/v*) β-mercaptoethanol<br>0.25 % (*w/v*) bromophenol blue<br>30 % glycerol<br>0.25 % xylene cyanol
In vitro reactions	Tris assay buffer	50 mM Tris/ HCl, pH 7.5
In vitro reactions	Sodium phosphate assay buffer	50 mM sodium phosphate buffer, pH 7.5

4. General Chemical Methods

4.1 Extraction of Secondary Metabolites

Liquid fungal cultures were homogenized with a blender and filtered by vacuum filtration. The filtrate was acidified with 2 M HCl to pH 2-4. The media was extracted twice with an equal amount of ethyl acetate, or three times if the extract was used for preparative LCMS. The organic layers were dried over anhydrous $MgSO_4$. Solvent was removed with a rotary evaporator. The extract was then dissolved in an appropriate amount of solvent (methanol, acetonitrile, H_2O) filtered with glass wool and analyzed by LCMS.

4.2 Protein Mass Spectrometry

MS data was obtained using a Quattro API mass spectrometer Waters system, which consists of a Waters 2767 autosampler and a Waters 2545 pump. Proteins were measured by direct injection (no column installed) of a protein solution (approx. 1 mg/mL) in a 50/50 mix of water/acetonitrile (0.05 % formic acid) at 0.2 mL/min. The mass spectrometer scanned over a range of 500 - 1500 *m/z*. Deconvolution of the protein peak area was done with MaxEnt® to give the molecular weight.[15,16]

4.3 High Resolution Mass Spectrometry

High Resolution Mass Spectrometry (HRMS) was performed with a Q-TOF Premier mass spectrometer (Waters) coupled to an Acquity UPLC system (Waters) by the MS department of the department of Organic Chemistry, Leibniz University Hannover.

4.4 Liquid Chromatography Mass Spectrometry

4.4.1 Analytical Liquid Chromatography Mass Spectrometry. Analytical liquid chromatography mass spectrometry (LCMS) data was obtained using a Waters LCMS system, which consists of a Waters 2767 autosampler, Waters 2545 pump, a Phenomenex Kinetex column (2.6 µm, C_{18}, 100 Å, 4.6 × 100 mm) equipped with a Phenomenex Security Guard precolumn (Luna, C_5, 300 Å). The system was used at a flow rate of 1 mL/min. Detection was carried out by a diode array detector (Waters 2998) in the range 210 to 600 nm and an ELSD detector (Waters 2424) together with a mass spectrometer, Waters SQD-2 mass detector, operating simultaneously in ES^+ and ES^- modes between 150 and 1000 *m/z*. Typically, a solvent gradient was run over 15 min starting at 10 % acetonitrile/ 90 % HPLC grade water (0.05 % formic acid) and ramping to 90 % acetonitrile.

4.4.2 Preparative Liquid Chromatography Mass Spectrometry. Preparative LCMS was used to purify chemoenzymatically and chemically synthesized compounds or compounds from microbial extracts. The used system is a Waters mass-directed auto-purification system, which consists of a Waters 2767 autosampler, Waters 2545 pump, a Phenomenex Kinetex Axia column (5 µm, C_{18}, 100 Å, 21.2 × 250 mm) equipped with a Phenomenex Security Guard precolumn (Luna, C_5, 300 Å). The system was used at a flow rate of 20 mL/min. Typically, a solvent gradient was run over 15 min starting at 10 % acetonitrile (solvent B, with 0.045 % formic acid) and 90 % HPLC grade water (solvent A; with 0.05 % formic acid) and ramping to 90 % solvent B. The post-column flow was split (100 : 1) and the minority flow was made up with HPLC-grade (1 : 1 solvent A/B) to 1.0 mL/ min for simultaneous analysis by diode array (Waters 2998) in the range 210 to 600 nm, an ELSD

detector (Waters 2424) together with a mass spectrometer, Waters SQD-2 mass detector, operating simultaneously in ES^+ and ES^- modes between 150 and 1000 *m/z*. Purification was done mass-directed. Compounds were fractionated and collected into glass test tubes and combined fractions were lyophilized after residual solvent was removed *in vacuo*. Compounds were stored at - 20 °C. Mass data was analyzed with MassLynx™ (Waters).

4.5 Nuclear Magnetic Resonance

Nuclear Magnetic Resonance (NMR) measurements were performed by the NMR department of Organic Chemistry department, Leibniz University Hannover. NMR data were recorded using either a Bruker Ultrashield 500 (^1H 500 MHz, ^{13}C 125 MHz), Bruker Ascend 600 (^1H 600 MHz, ^{13}C 150 MHz), Bruker Avance III 600 (^1H 600 MHz, ^{13}C 150 MHz) spectrometer. Chemical shifts are shown in parts per million (ppm) and referenced to solvents. The coupling constants *J* are given in Hz.

5. EXPERIMENT 1 (*ctrR4* + *ctrR3* + *ctrACS*) *in vivo*

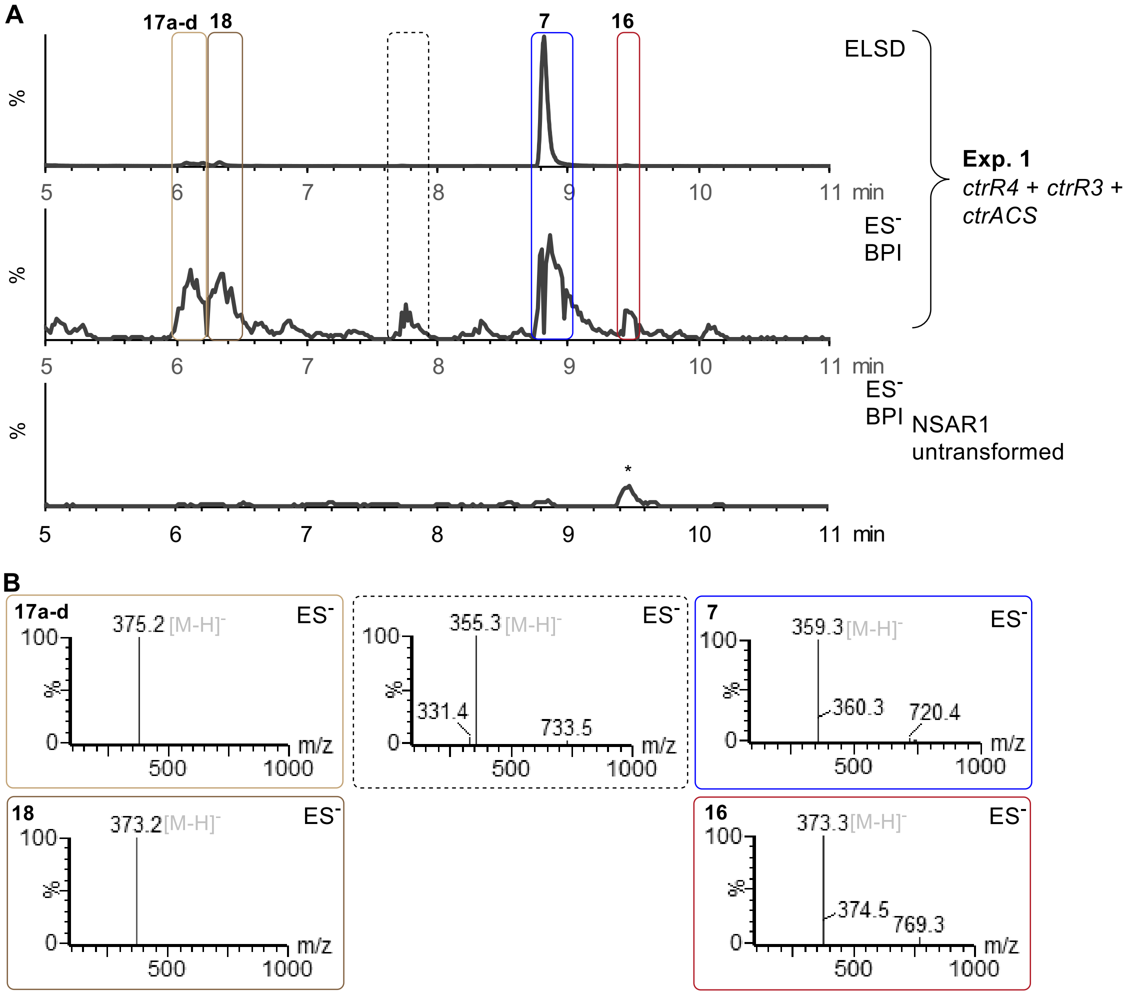

Figure S5 Heterologous expression of the structural genes of the *ctr* BGC: *ctrR4, ctrR3, ctrACS*. **A**, LCMS chromatograms of extracts of *A. oryzae* NSAR1 transformant and untransformed control strain (arbitrary units, ELSD smoothed in Excel, * = unrelated compound); **B**, ES$^-$ spectra of compounds detected in *A. oryzae* NSAR1 transformants.

5.1 Chacterisation of CJ-13,982, 7

Table S12 Chemical shifts of CJ-13,982 **7** in CD_3OD (600 MHz).

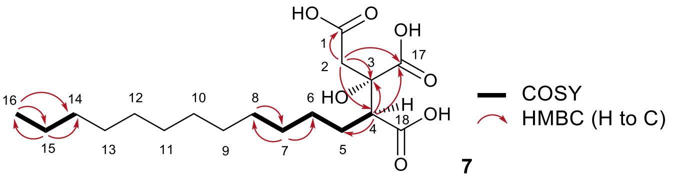

7

Chemical Formula: $C_{18}H_{32}O_7$
Mass: 360.21

	Experimentally observed					Compound **7** literature in CD_3OD[17]	
**Pos.**	**δ_H/ ppm (mult, *J* in Hz)**	**δ_C/ ppm**	**C-type**	**HMBC H to C**	**COSY**	**δ_H/ ppm (mult, *J* in Hz)**	**δ_C/ ppm**
**1**	-	173.9	CO	-	-	-	174.5
**2a**	3.07 (d, 16.4)	42.3	CH_2	1, 3, 17	2b	3.03 (d, 16.3)	43.1
**2b**	2.69 (d, 16.4)	42.3	CH_2	1, 3, 4, 17	2a	2.69 (d, 16.3)	43.1
**3**	-	76.8	C	-	-	-	77.0
**4**	2.65 (dd, 11.8, 3)	54.8	CH	2, 3, 5, 18	5, 6	2.65 (m)	54.5
**5a**	1.81 (m)	28.1	CH_2	4, 6, 18	4, 5b, 6	1.81 (m)	28.3
**5b**	1.49 (m)	28.1	CH_2	6, 18	4, 5a, 6	1.49 (m)	28.3
**6**	1.3 (m)	28.8	CH_2	5, 7-14	5b, 7-14	1.17-1.39 (m)	28.8
**7†**	1.3 (m)	30.5	CH_2	6, 8-14	6-14	1.17-1.39 (m)	30.4
**8†**	1.3 (m)	30.5	CH_2	7-14	7-14	1.17-1.39 (m)	30.5
**9†**	1.3 (m)	30.5	CH_2	7-14	7-14	1.17-1.39 (m)	30.6
**10†**	1.3 (m)	30.7	CH_2	7-14	7-14	1.17-1.39 (m)	30.7
**11†**	1.3 (m)	30.7	CH_2	7-14	7-14	1.17-1.39 (m)	30.7
**12†**	1.3 (m)	30.8	CH_2	7-14	7-14	1.17-1.39 (m)	30.7
**13†**	1.3 (m)	30.8	CH_2	7-14	7-14	1.17-1.39 (m)	30.8
**14**	1.3 (m)	33.1	CH_2	7-14, 15	7-14	1.17-1.39 (m)	33.1
**15**	1.3 (m)	23.7	CH_2	14, 16	14, 16	1.17-1.39 (m)	23.7
**16**	0.90 (t, 7)	14.4	CH_3	14, 15	15	0.90 (t, 6.9)	14.4
**17**	-	176.7	CO	-	-	-	177.5
**18**	-	176.2	CO	-	-	-	177.0

† These signals are indistinguishable from each other

Table S13 Optical rotation of CJ-13,982 **7** in CD_3OD.

**Experimentally observed**	**Compound 7 literature in acetone**[18]	**Compound 7 literature in acetone**[19]	**Compound 7 literature in acetone**[17]
$[\alpha]_D^{23}$ -12.7 (c 0.22, MeOH)	$[\alpha]_D^{23}$ -14.9 (c 0.52, acetone)	$[\alpha]_D^{26}$ -18.3 (c 2.6, acetone)	$[\alpha]_D^{25}$ -14.1 (c 0.25, acetone)

A

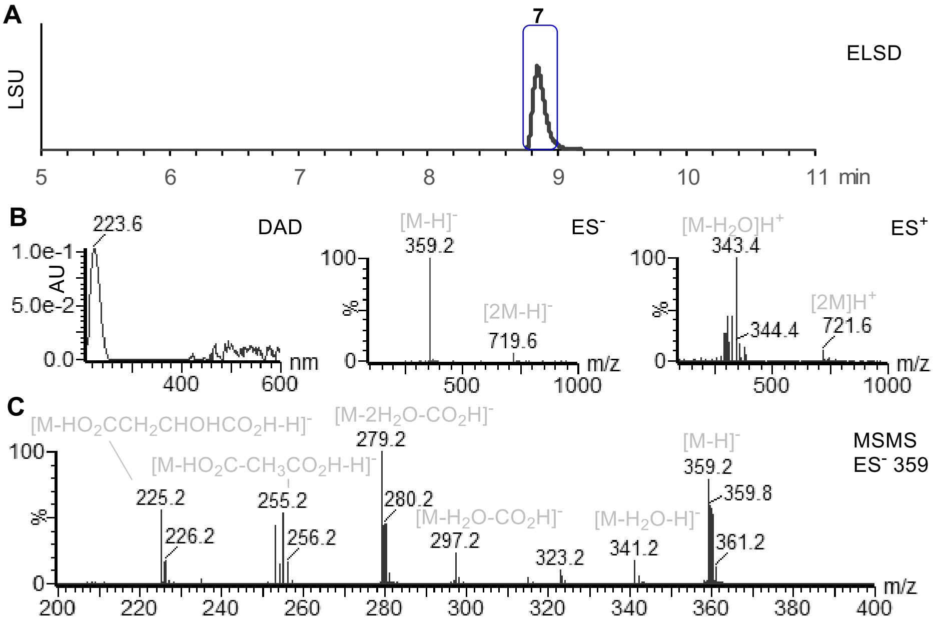

D

Elemental Composition Report

Single Mass Analysis

Tolerance = 20.0 PPM / DBE: min = -1.5, max = 50.0
Element prediction : Off
Number of isotopes peaks used for i-FIT = 3

Monoisotopic Mass, Even Electron Ions
75 formula(e) evaluated with 3 results within limits (all results (up to 1000) for each mass)
Elements Used:
C: 0-30 H: 0-50 O: 0-8 Na: 0-1

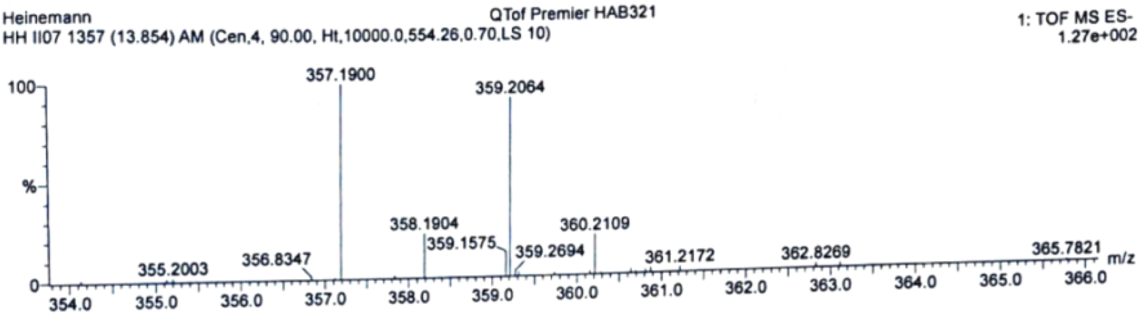

Minimum:				-1.5			
Maximum:		5.0	20.0	50.0			
Mass	Calc. Mass	mDa	PPM	DBE	i-FIT	i-FIT (Norm)	Formula
359.2064	359.2070	-0.6	-1.7	3.5	24.7	0.6	C18 H31 O7
	359.2046	1.8	5.0	0.5	25.1	1.0	C16 H32 O7 Na
	359.2011	5.3	14.8	12.5	27.0	2.9	C25 H27 O2

Figure S6 LCMS characterisation and structure elucidation of **7**. **A**, ELSD trace of **7** (arbitrary units; smoothed in Excel); **B**, DAD, ES^- and ES^+ spectra of **7**; **C**, MSMS data obtained for **7**; **D**, HRMS analysis and calculated molecular formula of **7**.

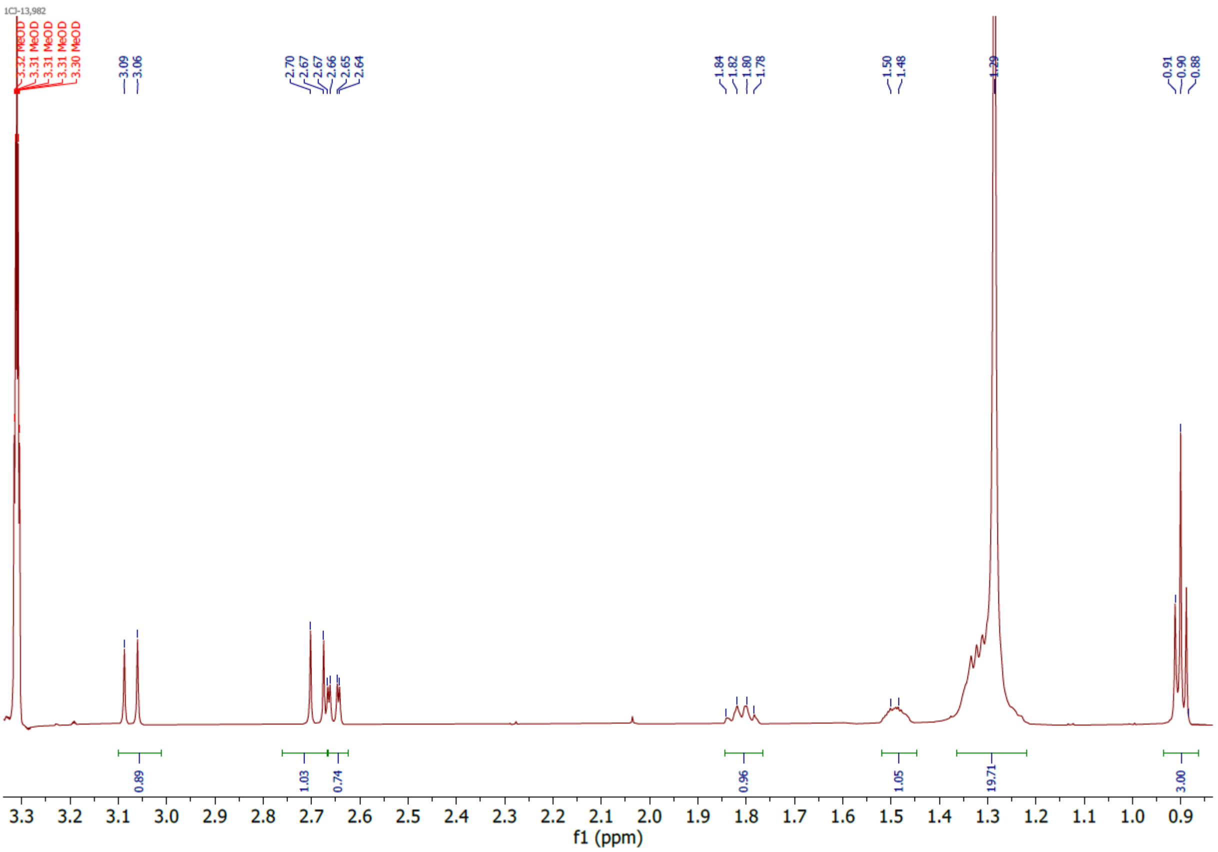

Figure S7 ^{1}H NMR of **7** in CD_3OD (600 MHz). See Table S12 for assignment.

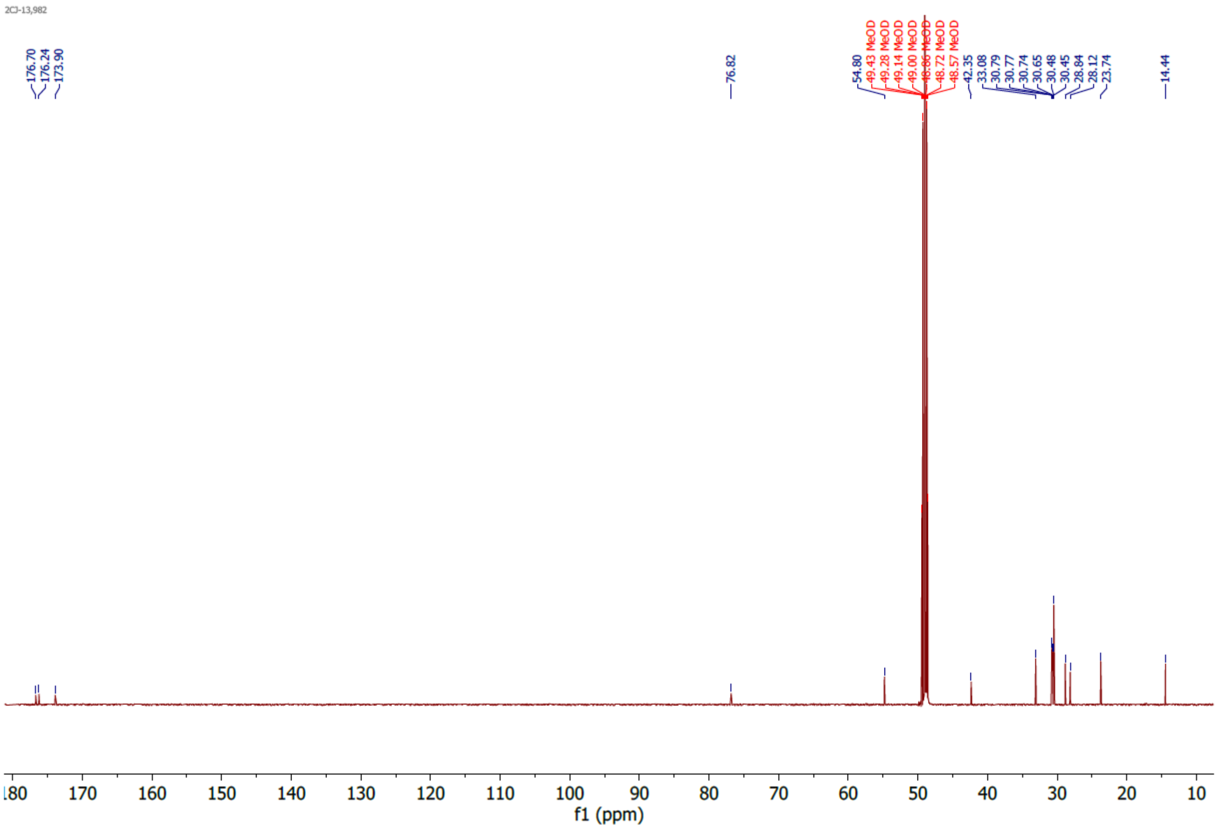

Figure S8 ^{13}C NMR of **7** in CD_3OD (150 MHz). See Table S12 for assignment.

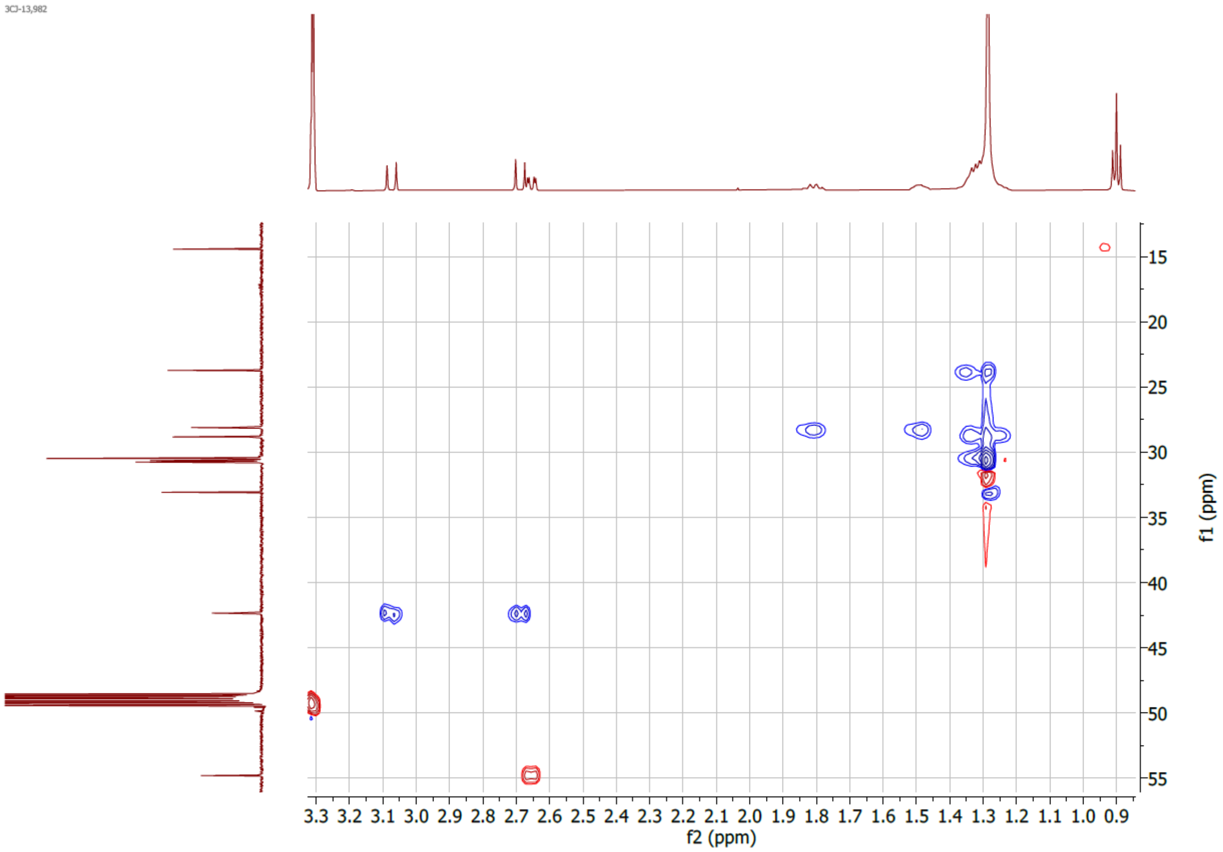

Figure S9 HSQC NMR of 7 in CD_3OD.

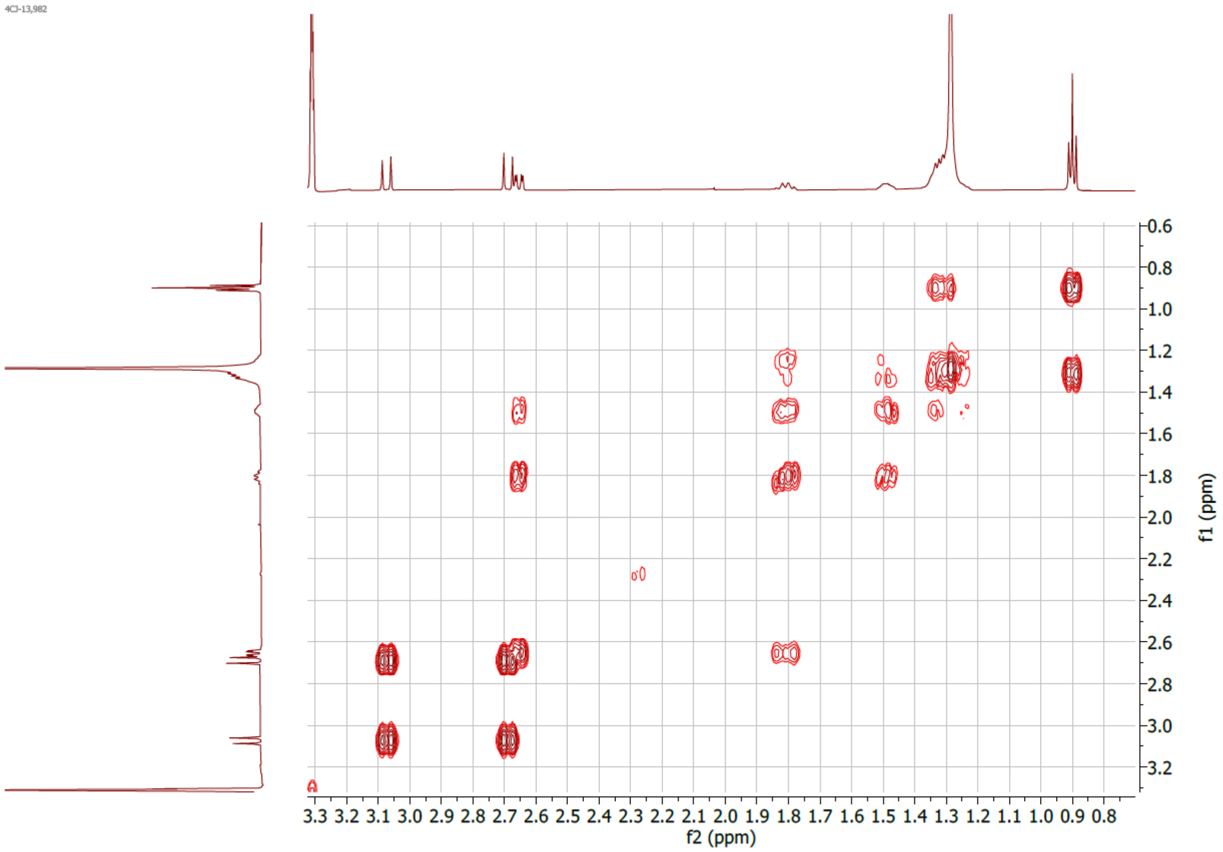

Figure S10 ^{1}H, ^{1}H COSY NMR of **7** in CD_3OD.

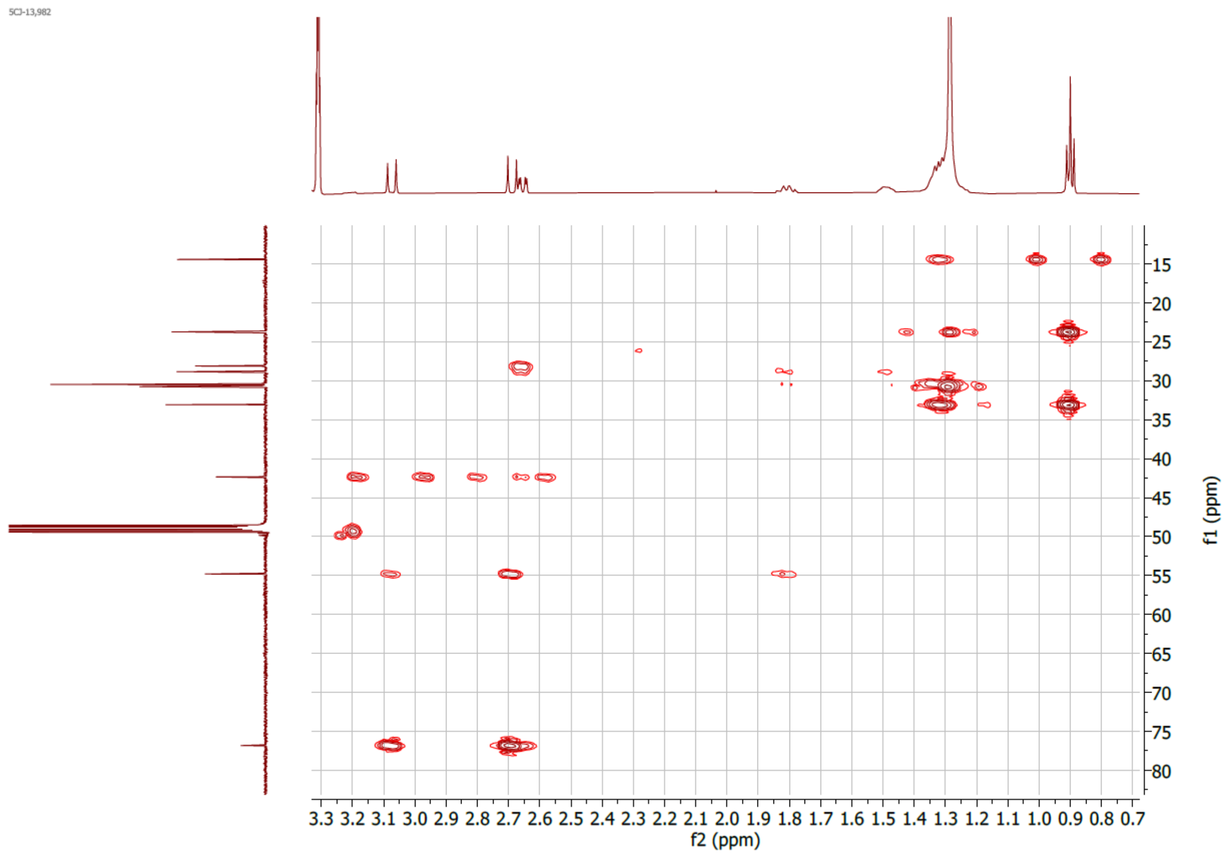

Figure S11 HMBC NMR of **7** in CD_3OD.

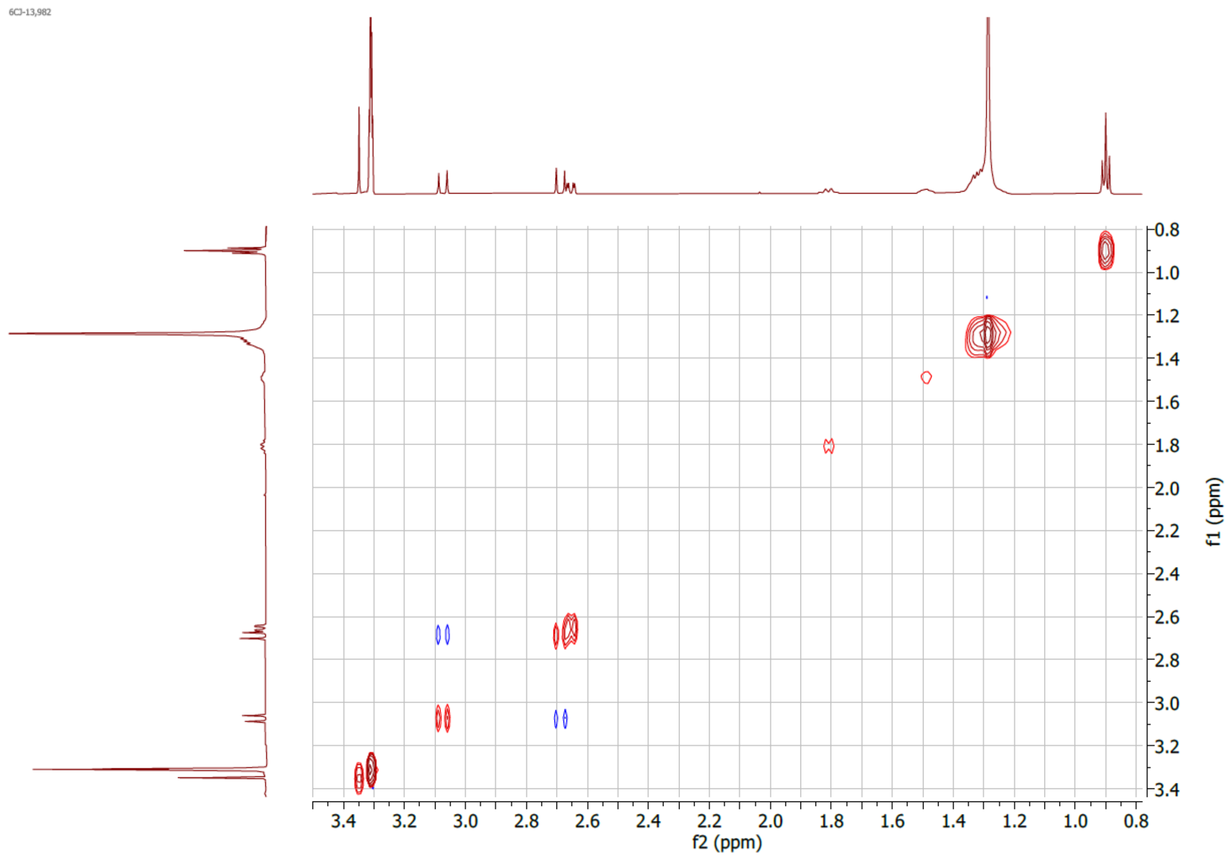

Figure S12 NOESY NMR of **7** in CD_3OD.

5.2 Chacterisation of CJ-13,982 17-methyl ester, 16

Table S14 Chemical shifts of **16** in CD_3OD (600 MHz).

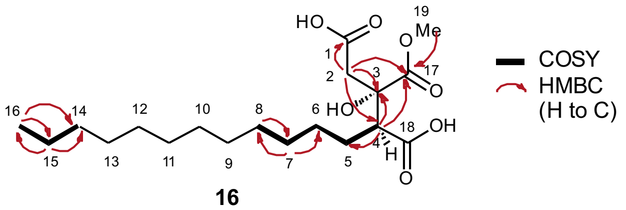

16

Chemical Formula: $C_{19}H_{34}O_7$
Exact Mass: 374.23

Pos.	δ_H/ ppm (mult, *J* in Hz)	δ_C/ ppm	C-type	HMBC H to C	COSY
**1**	-	174.5	CO	-	-
**2a**	3.03 (d, 16.1)	44.3	CH_2	1, 3,	2b
**2b**	2.64 (d, 16.1)	44.3	CH_2	1, 3, 4, 17	2a
**3**	-	77.5	C	-	-
**4**	2.70 (dd, 11.7, 2.8)	54.4	CH	3, 5, 18	5, 6
**5a**	1.77 (m)	28.6	CH_2	-	4, 5b, 6
**5b**	1.40 (m)	28.6	CH_2	-	5a, 6
**6**	1.37 (m)	28.9	CH_2	5	5, 7
**7†**	1.3 (m)	30.5	CH_2	6, 8-14	6-14
**8†**	1.3 (m)	30.6	CH_2	7-14	7-14
**9†**	1.3 (m)	30.6	CH_2	7-14	7-14
**10†**	1.3 (m)	30.7	CH_2	7-14	7-14
**11†**	1.3 (m)	30.8	CH_2	7-14	7-14
**12†**	1.3 (m)	30.8	CH_2	7-14	7-14
**13†**	1.3 (m)	30.8	CH_2	7-14	7-14
**14**	1.27 (m)	33.1	CH_2	7-14	7-14
**15**	1.3 (m)	23.7	CH_2	14, 16	14, 16
**16**	0.90 (app. q, 7.1)	14.4	CH_3	14, 15	15
**17**	-	175.6	CO	-	-
**18**	-	177.4	CO	-	-
**19**	3.73 (s)	52.8	CH_3	17	-

† These signals are indistinguishable from each other.

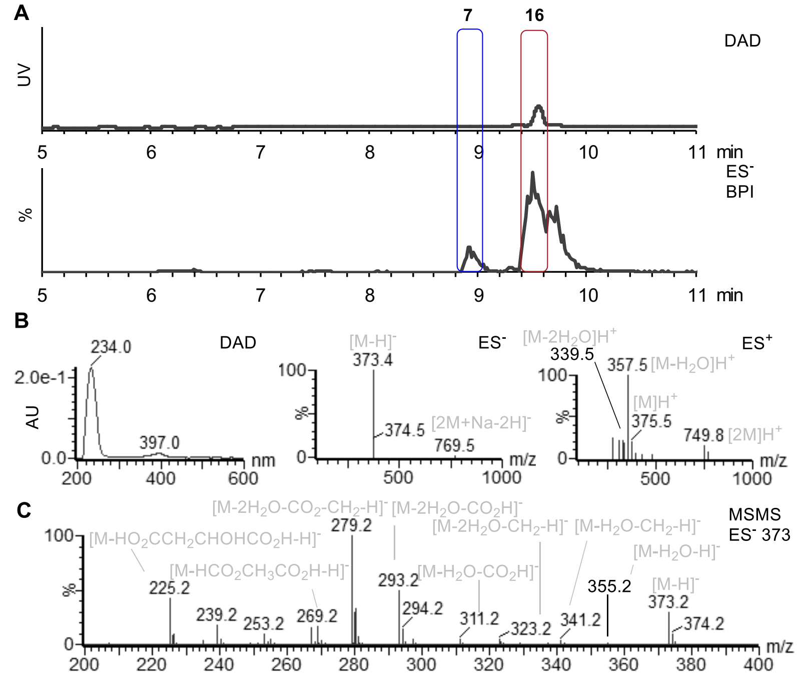

D

Elemental Composition Report

Single Mass Analysis
Tolerance = 20.0 PPM / DBE: min = -1.5, max = 50.0
Element prediction : Off
Number of isotopes peaks used for i-FIT = 3

Monoisotopic Mass, Even Electron Ions
463 formula(e) evaluated with 4 results within limits (all results (up to 1000) for each mass)
Elements Used:
C: 0-22 H: 0-35 N: 0-4 O: 0-8 S: 0-3

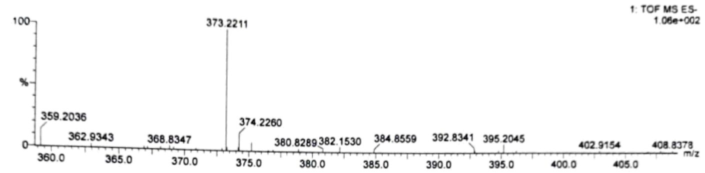

Mass	Calc. Mass	mDa	PPM	DBE	i-FIT	i-FIT (Norm)	Formula
Minimum:				-1.5			
Maximum:		5.0	20.0	50.0			
373.2211	373.2226	-1.5	-4.0	3.5	14.2	1.6	C19 H33 O7
	373.2240	-2.9	-7.8	8.5	15.0	2.4	C20 H29 N4 O3
	373.2161	5.0	13.4	3.5	13.4	0.8	C18 H33 N2 O4 S
	373.2273	-6.2	-16.6	3.5	14.1	1.4	C17 H33 N4 O3 S

Figure S13 LCMS characterisation and structure elucidation of **16**. **A**, DAD and ES$^-$ traces of **16** (arbitrary units); **B**, DAD, ES$^-$ and ES$^+$ spectra of **16**; **C**, MSMS analysis of **16**; **D**, HRMS analysis.

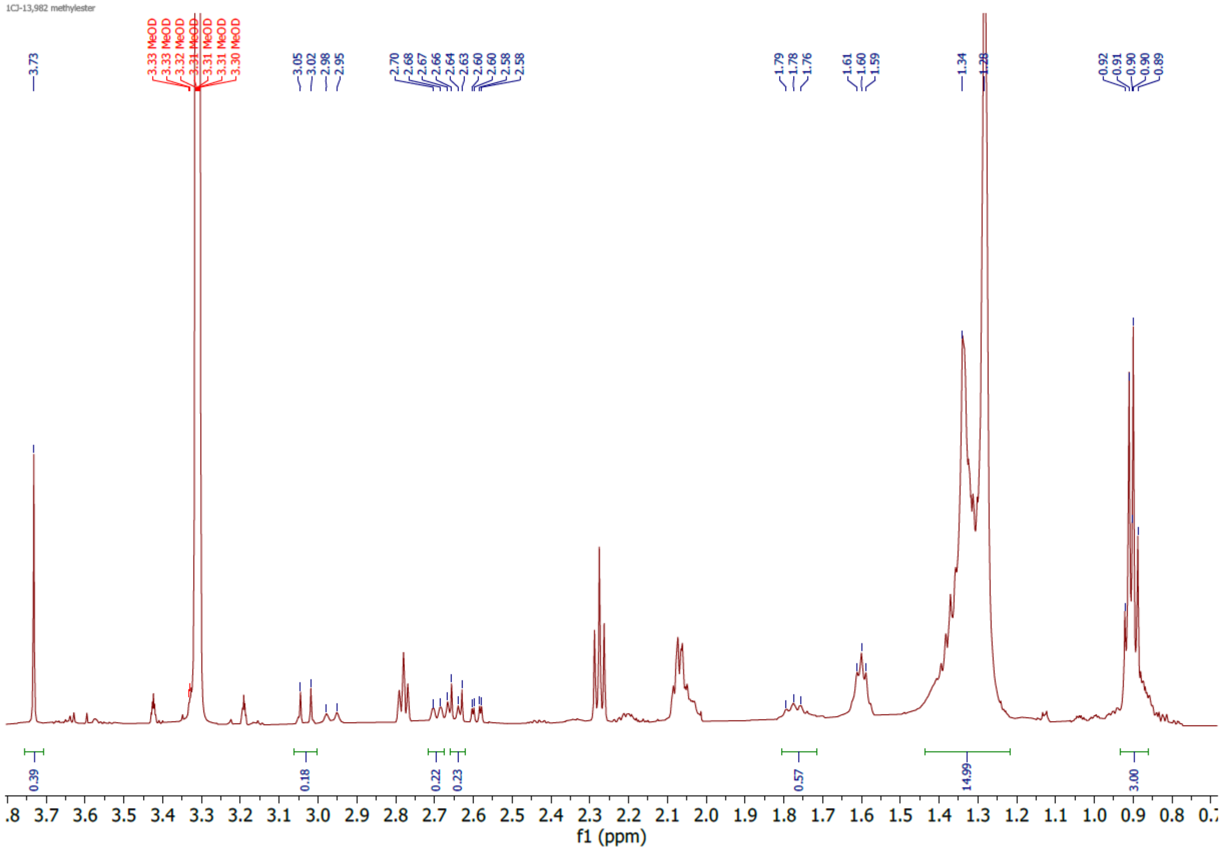

Figure S14 ^{1}H NMR of **16** in CD_3OD (600 MHz). See Table S14 for assignment.

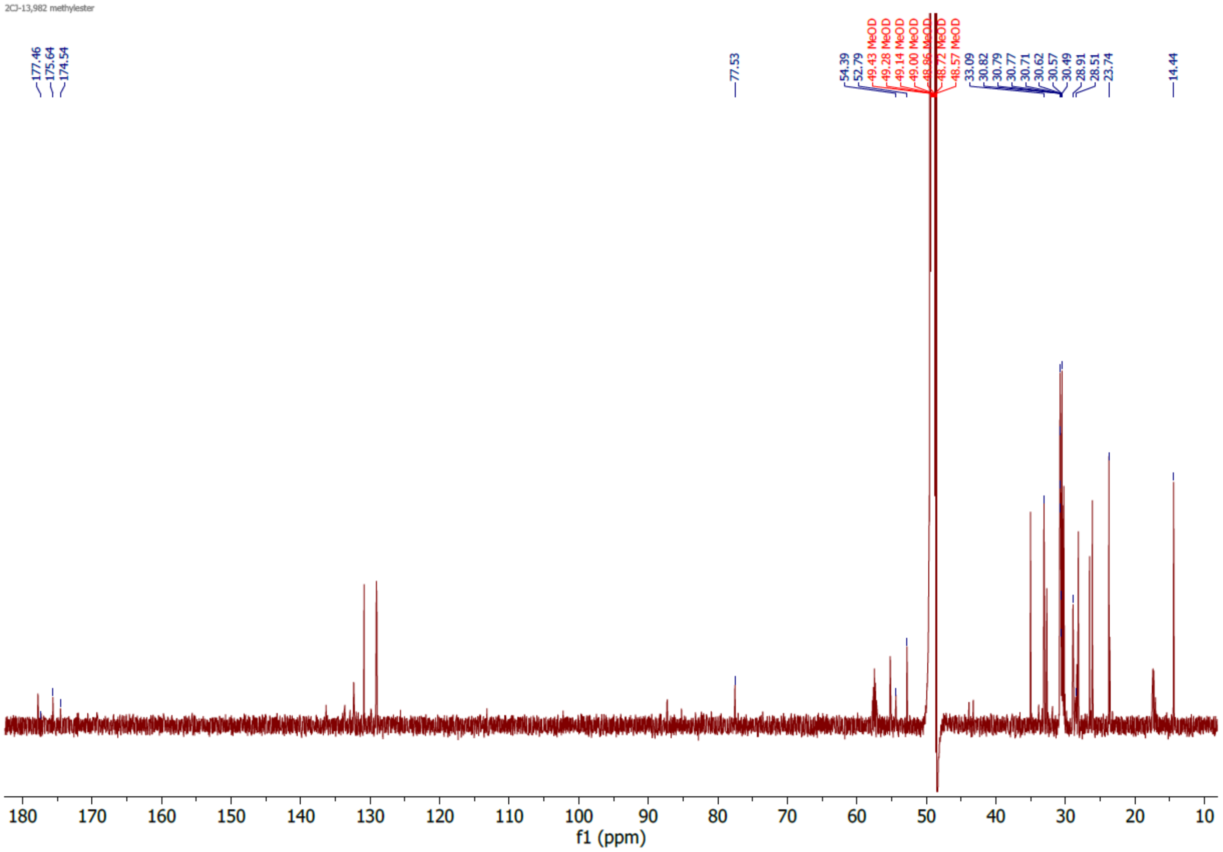

Figure S15 ^{13}C NMR of **16** in CD_3OD (150 MHz). See Table S14 for assignment.

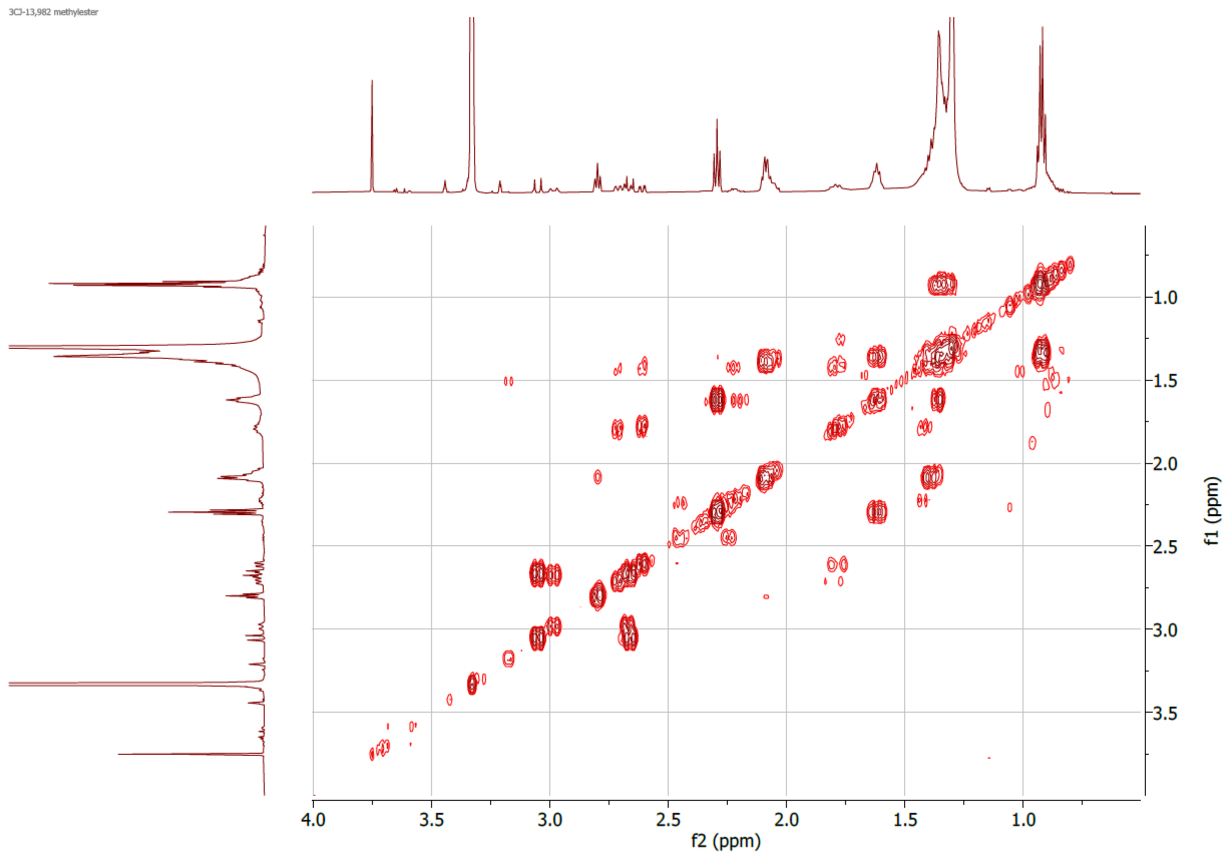

Figure S16 ^{1}H, ^{1}H COSY NMR of **16** in CD_3OD.

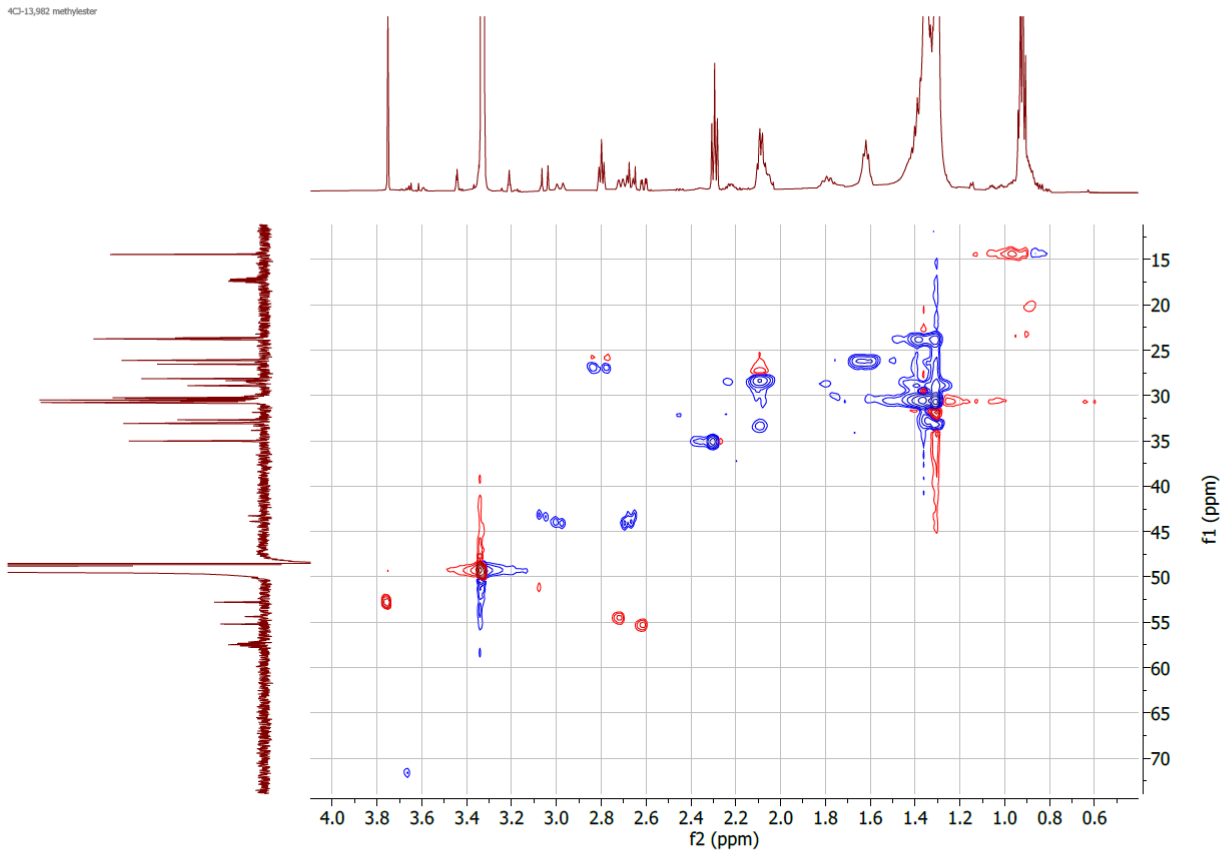

Figure S17 HSQC NMR of **16** in CD_3OD.

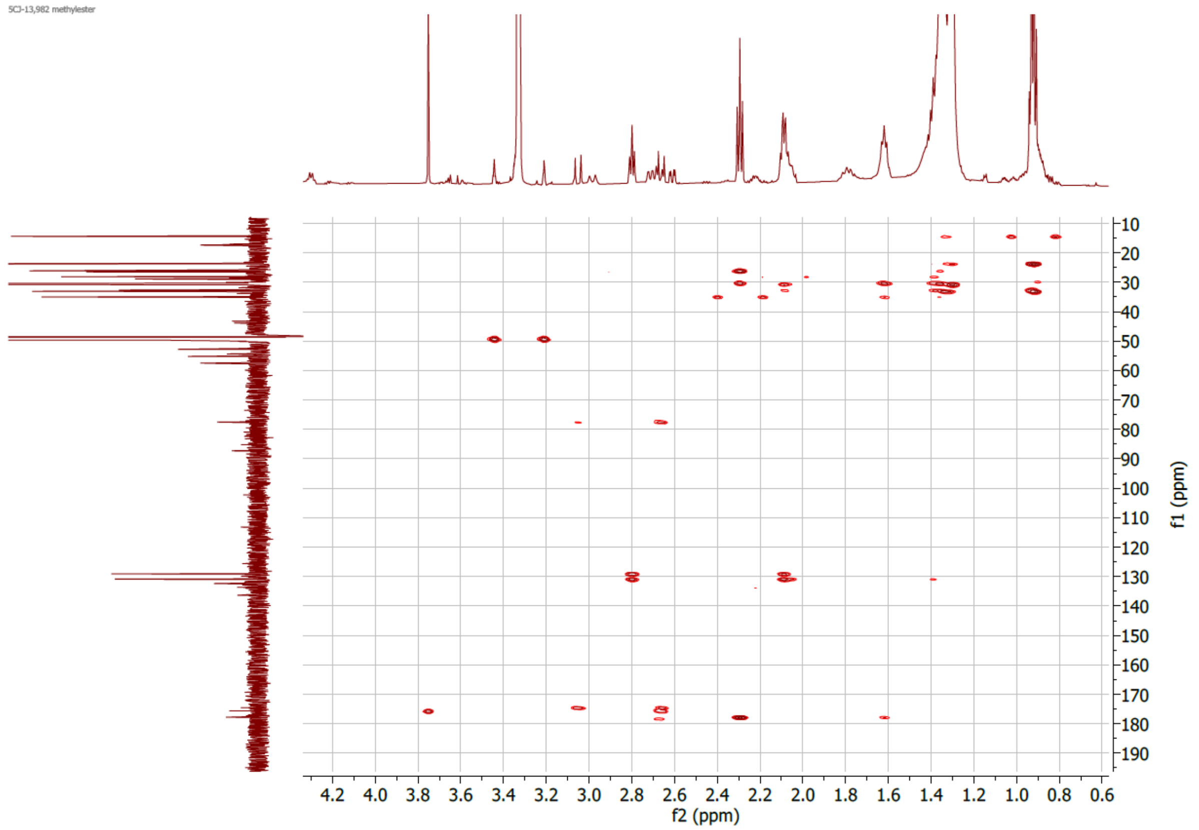

Figure S18 HMBC of **16** in CD_3OD.

5.3 Chacterisation of Inseparable Mixture of 13,14,15 hydroxy-CJ-13,982, 17a-c and 15-oxo- CJ-13,982 17d

Table S15 Chemical shifts of common NMR signals of **17a - d** in CD_3OD (600 MHz).

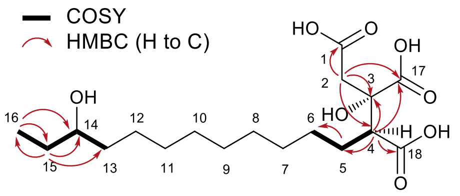

17a

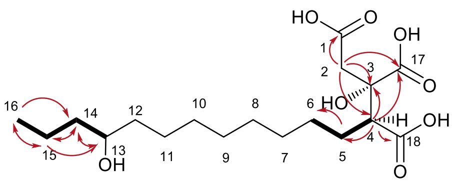

17b

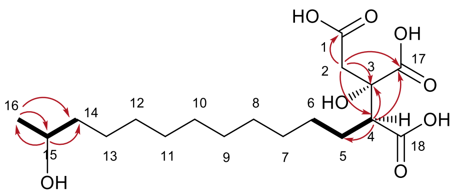

17c

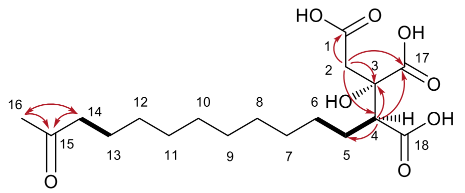

17d

Pos.	δH/ ppm (mult, *J* in Hz)	δC/ ppm	C-type	HMBC H to C	COSY
**1**	-	174.1	CO	-	-
**2a**	3.05 (d, 16.2)	42.6	CH_2	1, 3	2b
**2b**	2.68 (d, 16.2)	42.6	CH_2	1, 3, 4, 17	2a
**3**	-	76.9	C	-	-
**4**	2.65 (m)	54.7	CH	3, 5, 17, 18	5
**5a**	1.81 (m)	28.2	CH_2	4, 6, 18	4, 6
**5b**	1.47 (m)	28.2	CH_2	6	4, 6
**6**	1.35 (m)	28.8	CH_2	7-14	5, 7
**7†**	1.3 (m)	30.2	CH_2	6, 7-14	6, 8
**8†**	1.3 (m)	30.5	CH_2	7-14	7-14
**9†**	1.3 (m)	30.6	CH_2	7-14	7-14
**10†**	1.3 (m)	30.6	CH_2	7-14	7-14
**11†**	1.3 (m)	30.7	CH_2	7-14	7-14
**12†**	1.3 (m)	30.8	CH_2	7-14	7-14
**17**	-	177.0	CO	-	-
**18**	-	176.5	CO	-	-

† These signals are indistinguishable from each other.

Table S16 Chemical shifts of **17a** in CD_3OD (600 MHz).

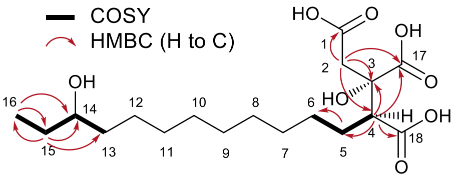

17a

Pos.	δH/ ppm (mult, *J* in Hz)	δC/ ppm	C-type	HMBC H to C	COSY
13	1.39 (m)	40.7	CH_2	-	14, 12
14	3.43 (m)	73.9	CH	-	-
15	1.47 (m)	31.0	CH_2	13, 14, 16	14, 16
16	0.93 (t, 7.5)	10.3	CH_3	14, 15	15

Table S17 Chemical shifts of **17b** in CD_3OD (600 MHz).

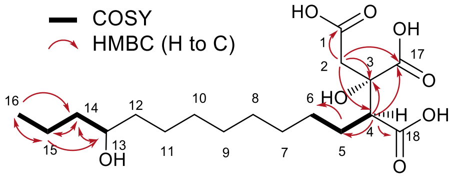

17b

Pos.	δ_H/ ppm (mult, *J* in Hz)	δ_C/ ppm	C-type	HMBC H to C	COSY
**13**	3.52 (m)	72.2	CH	-	12, 14
**14**	1.39 (m)	40.7	CH_2	13, 15	13, 15
**15a**	1.48 (m)	19.7	CH_2	13, 14, 16	14, 16
**15b**	1.36 (m)	19.7	CH_2	13, 14, 16	14, 16
**16**	0.93 (t, 7.2)	14.5	CH_3	14, 15	15

Table S18 Chemical shifts of **17c** in CD_3OD (600 MHz).

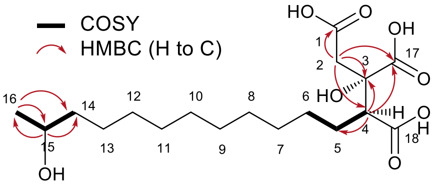

17c

Pos.	δ_H/ ppm (mult, *J* in Hz)	δ_C/ ppm	C-type	HMBC H to C	COSY
**13**†	1.3 (m)	30.8	CH_2	7-14	7-14
**14a**	1.46 (m)	40.0	CH_2	-	15
**14b**	1.39 (m)	40.0	CH_2	-	15
**15**	3.69 (m)	68.6	CH	14, 16	14, 16
**16**	1.14 (d, 6.2)	23.5	CH_3	14, 15	15

† This signal is not distinguishable from position 7 - 12 in Table S15.

Table S19 Chemical shifts of **17d** in CD_3OD (600 MHz).

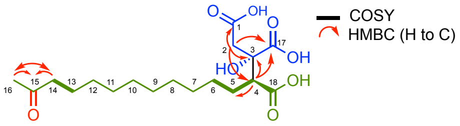

Chemical Formula: $C_{18}H_{30}O_8$
Exact Mass: 374.2

Pos.	δ_H/ ppm (mult, *J* in Hz)	δ_C/ ppm	C-type	HMBC H to C	COSY
**12**†	1.3 (m)	30.8	CH_2	7-14	7-14
**13**	1.53 (m)	30.8	CH_2	12 etc	12 etc
**14**	2.47 (t, 7.4 Hz)	40.0	CH_2	13, 15, 16	13
**15**	-	212.3	C	-	-
**16**	2.12 (s)	23.5	CH_3	14, 15	-

† This signal is not distinguishable from position 7 - 12 in Table S15.

A

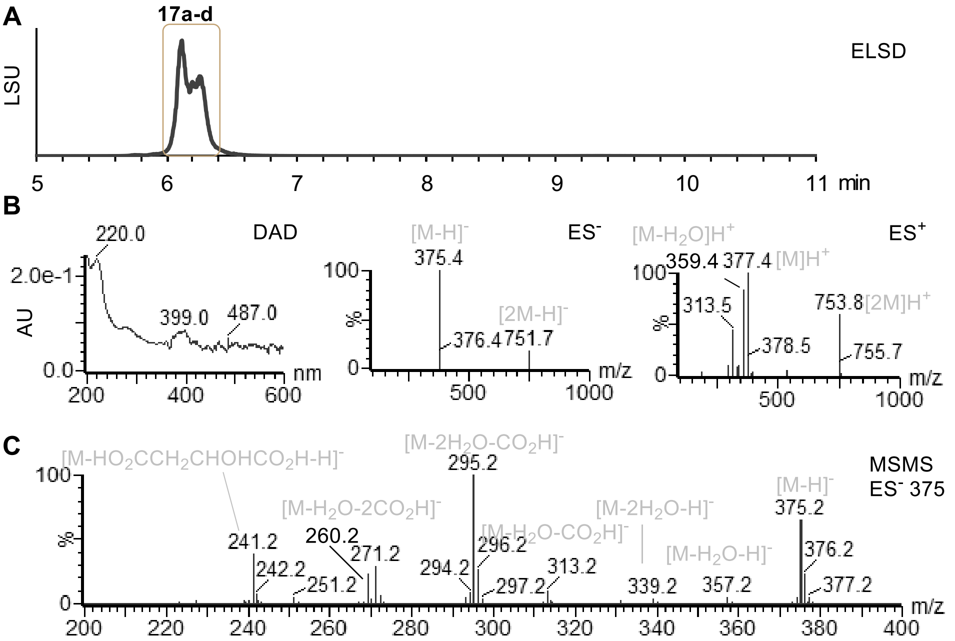

D

Elemental Composition Report

Single Mass Analysis
Tolerance = 20.0 PPM / DBE: min = -1.5, max = 50.0
Element prediction : Off
Number of isotopes peaks used for i-FIT = 3

Monoisotopic Mass, Even Electron Ions
466 formula(e) evaluated with 9 results within limits (all results (up to 1000) for each mass)
Elements Used:
C: 0-22 H: 0-35 N: 0-4 O: 0-8 S: 0-3

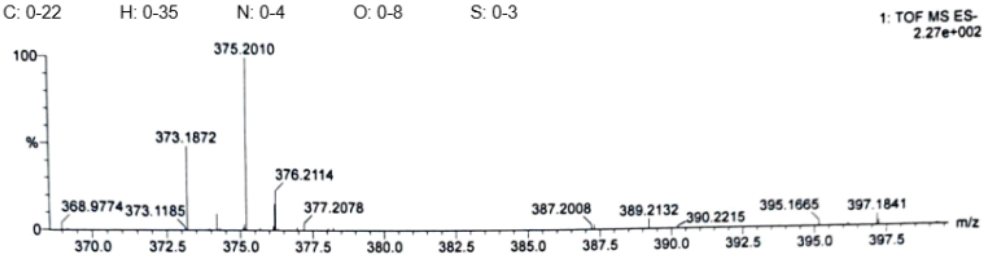

Minimum:				-1.5			
Maximum:		5.0	20.0	50.0			
Mass	Calc. Mass	mDa	PPM	DBE	i-FIT	i-FIT (Norm)	Formula
375.2010	375.2019	-0.9	-2.4	3.5	24.7	0.7	C18 H31 O8
	375.1994	1.6	4.3	7.5	27.8	3.8	C22 H31 O3 S
	375.2028	-1.8	-4.8	2.5	29.8	5.9	C19 H35 O3 S2
	375.2032	-2.2	-5.9	8.5	24.8	0.8	C19 H27 N4 O4
	375.1987	2.3	6.1	-1.5	30.6	6.6	C14 H35 N2 O5 S2
	375.2053	-4.3	-11.5	-1.5	27.9	3.9	C15 H35 O8 S
	375.1962	4.8	12.8	2.5	32.5	8.5	C18 H35 N2 S3
	375.1954	5.6	14.9	3.5	28.7	4.7	C17 H31 N2 O5 S
	375.2066	-5.6	-14.9	3.5	27.7	3.7	C16 H31 N4 O4 S

Figure S19 LCMS characterization and structure elucidation of **17a-d**. **A**, ELSD trace of **17a-d** (arbitrary units, smoothed in Excel); **B**, DAD, ES^- and ES^+ spectra of **17a-d**; **C**, MSMS analysis of 375.4 ion; **D**, HRMS analysis and calculated molecular formula of **17a-c**.

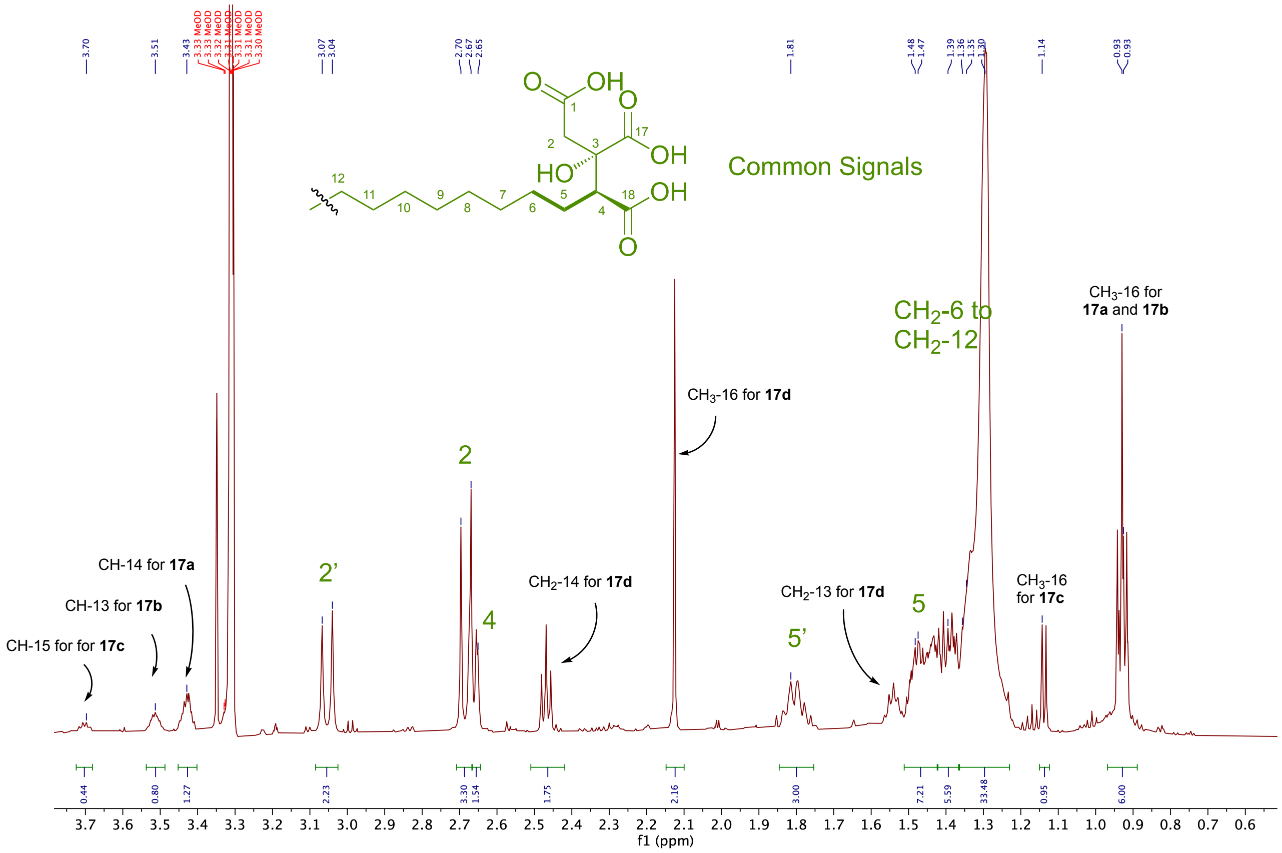

Figure S20 Annotated ^{1}H NMR spectrum of mixture of **17a-d** in CD_3OD.

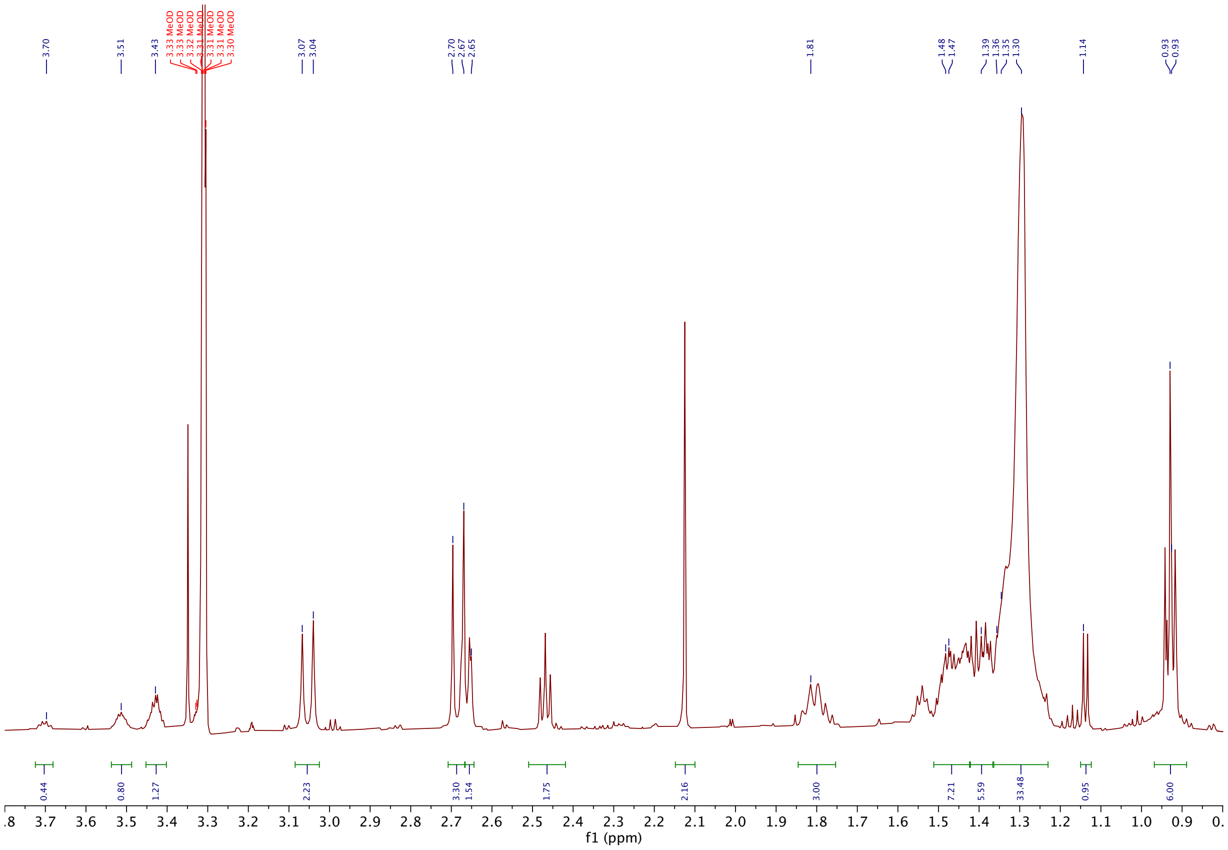

Figure S21 ^{1}H NMR of mixture of **17a-d** in CD_3OD.

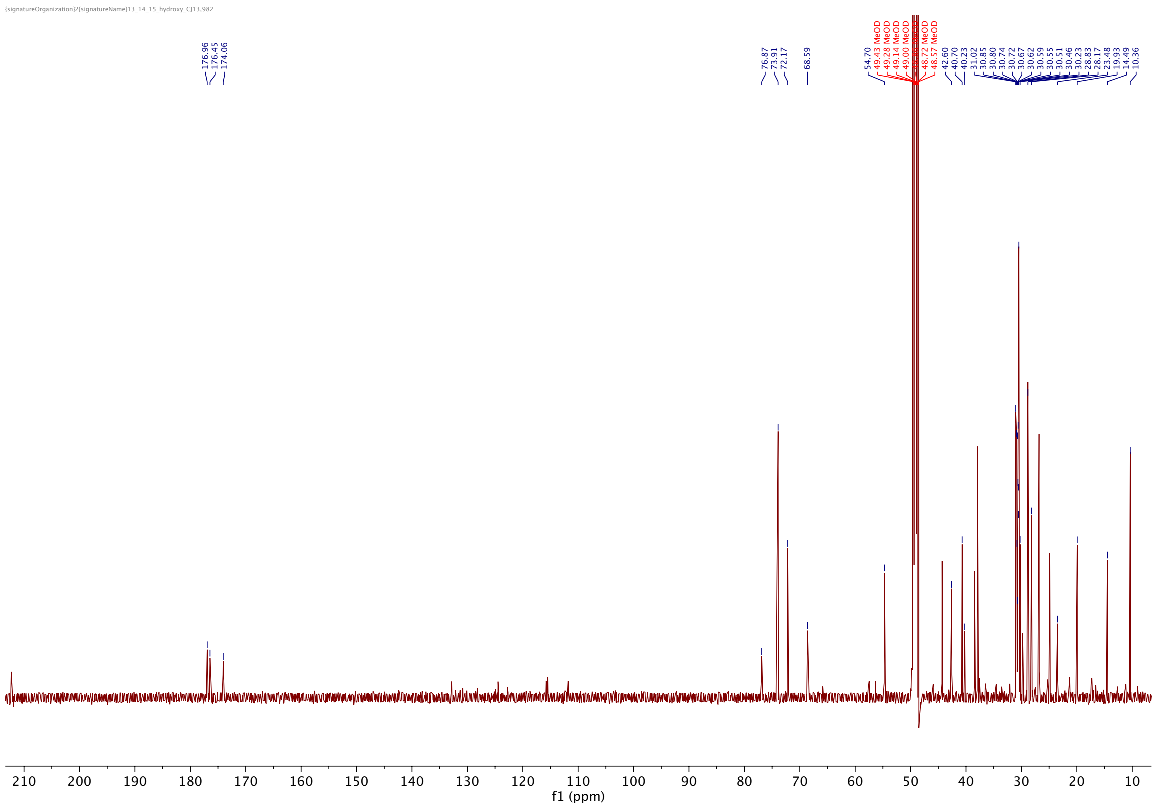

Figure S22 ^{13}C NMR of **17a-d** in CD_3OD.

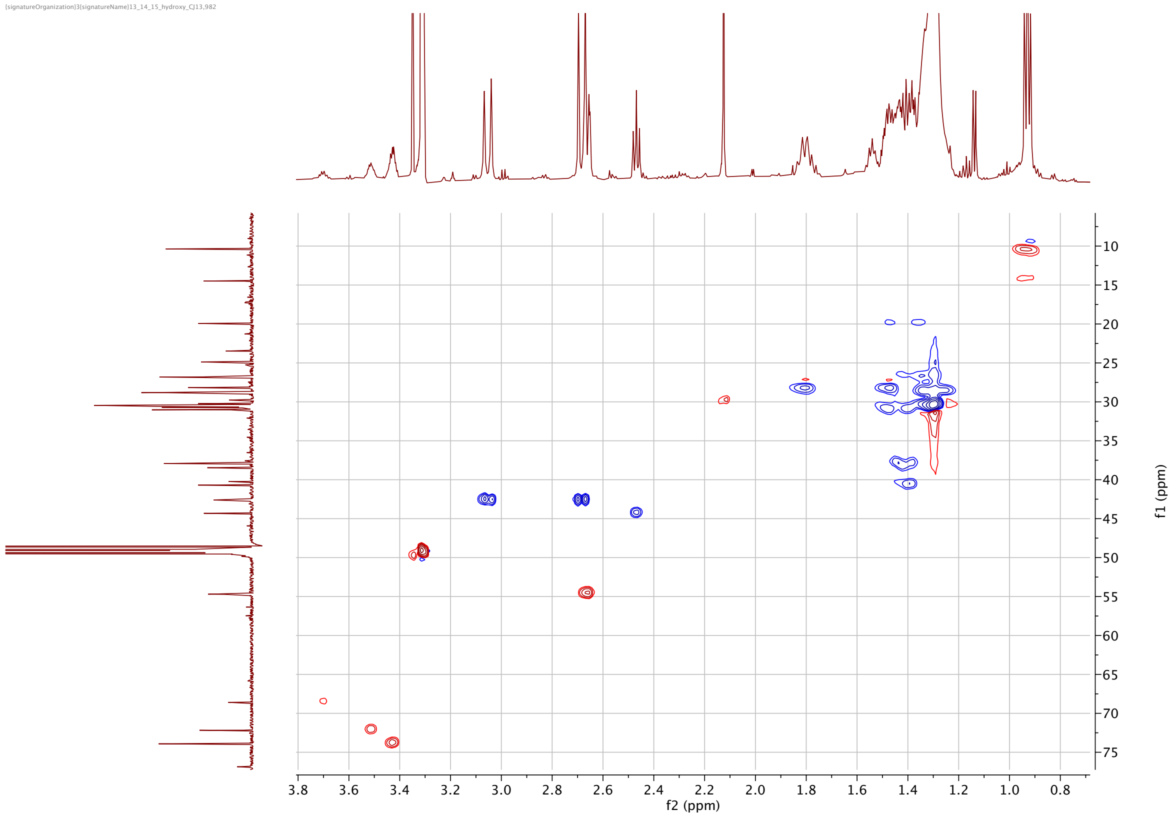

Figure S23 HSQC NMR of **17a-d** in CD_3OD.

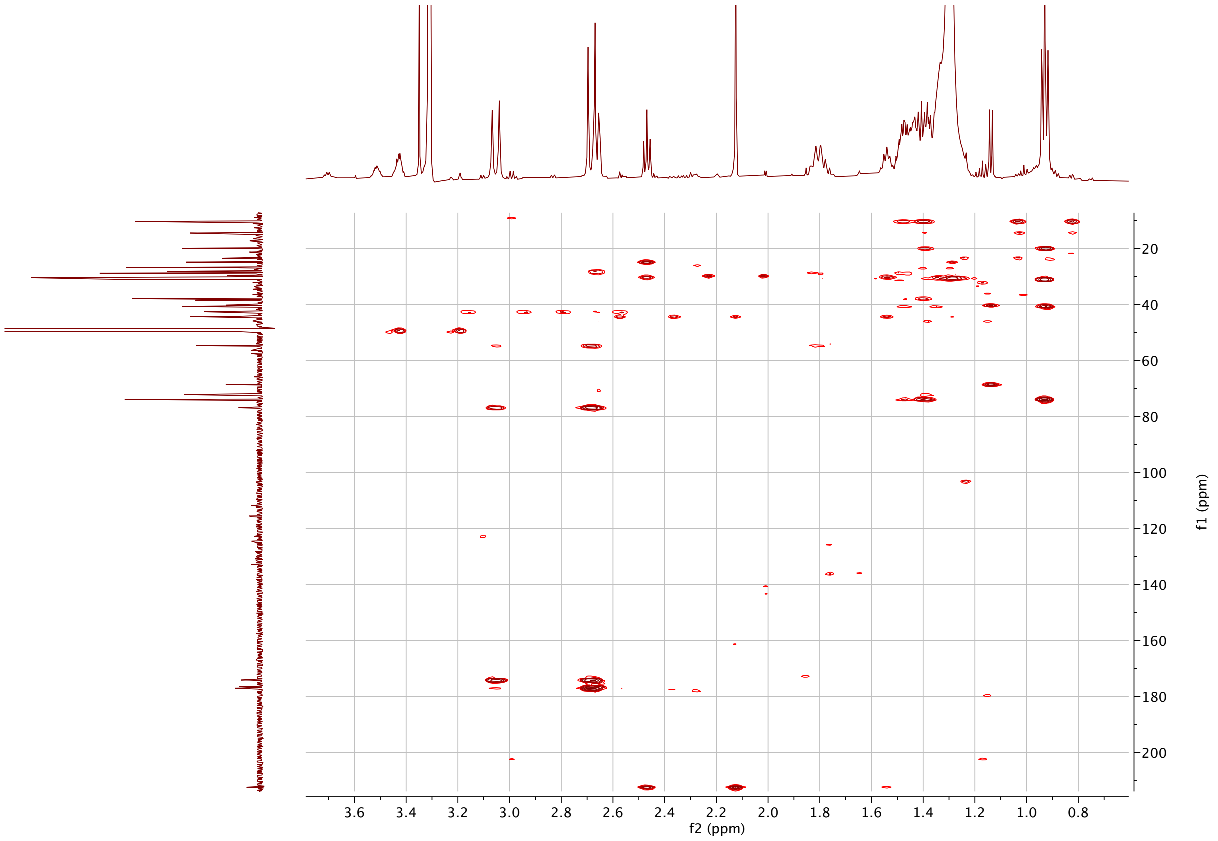

Figure S24 HMBC NMR of **17a-d** in CD_3OD.

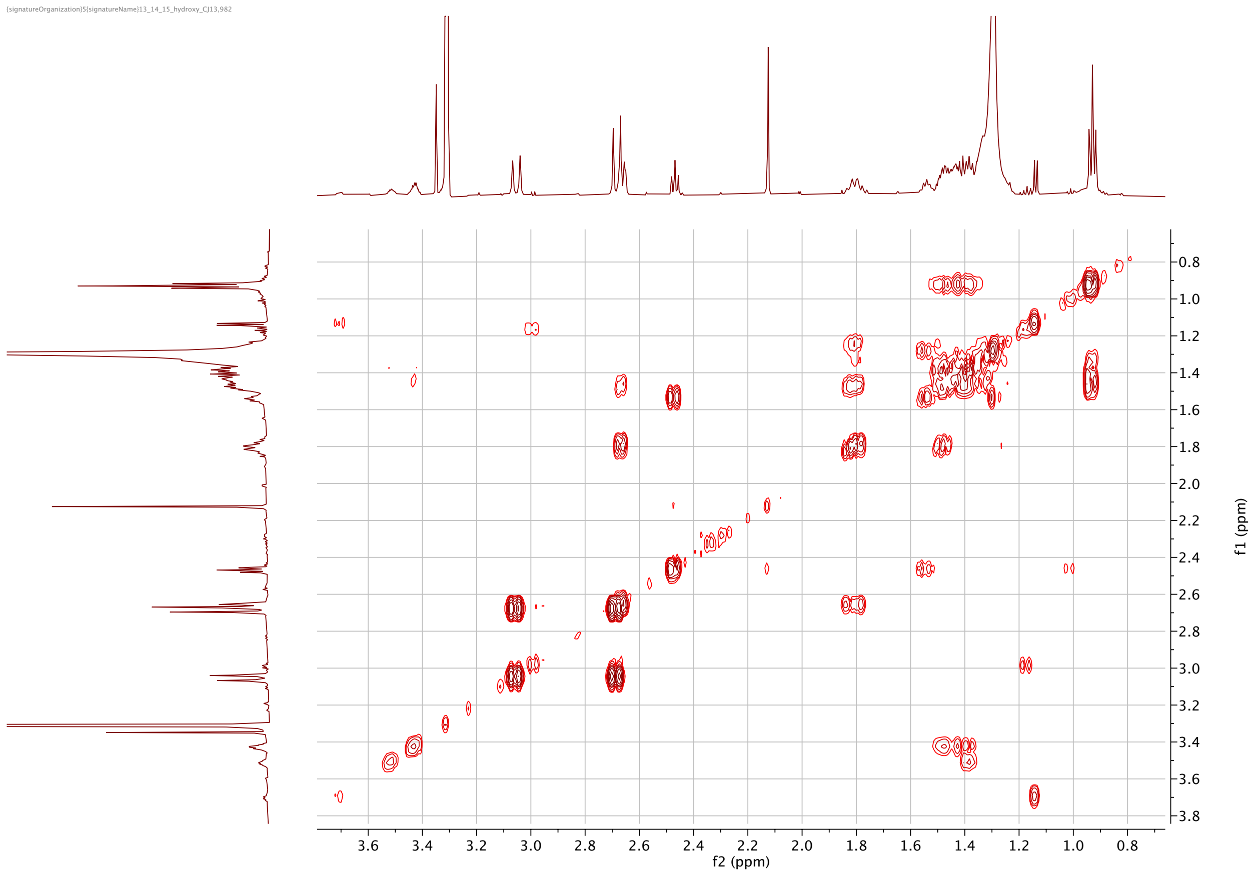

Figure S25 COSY NMR of **17a-d** in CD_3OD.

5.4 Chacterisation of 14-oxo-CJ-13,982, 18

Table S20 Chemical shifts of **18** in CD_3OD (600 MHz).

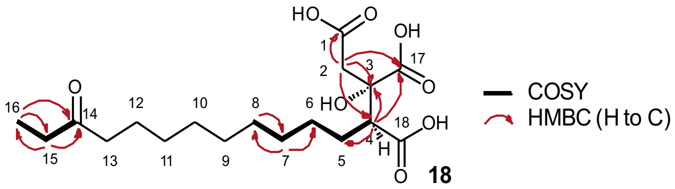

Chemical Formula: $C_{18}H_{30}O_8$
Exact Mass: 374.1941

Pos.	δ_H/ ppm (mult, *J* in Hz)	δ_C/ ppm	C-type	HMBC H to C	COSY
**1**	-	174.5	CO	-	-
**2a**	3.01 (d, 16.2)	43.1	CH_2	1, 3	2b
**2b**	2.67 (d, 16.2)	43.1	CH_2	1, 3, 4, 17	2a
**3**	-	77.0	C	-	-
**4**	2.68 (m)	54.5	CH	3, 5, 18	5a, b
**5a**	1.79 (m)	28.8	CH_2	4	4, 5b, 6
**5b**	1.43 (m)	28.8	CH_2	6	5a
**6**	1.3 (m)	29.5	CH_2	7-14	5b, 7-14
**7†**	1.3 (m)	30.5	CH_2	7-14	7-14
**8†**	1.3 (m)	30.6	CH_2	7-14	7-14
**9†**	1.3 (m)	30.6	CH_2	7-14	7-14
**10†**	1.3 (m)	30.7	CH_2	7-14	7-14
**11†**	1.3 (m)	30.8	CH_2	7-14	7-14
**12**	1.54 (m)	24.9	CH_2	11, 13	7-14
**13**	2.44 (m)	43.4	CH_2	11, 12	14
**14**	-	214.7	CO	-	-
**15**	2.46 (q, 7.3)	36.5	CH_2	16	14, 16
**16**	1.01 (t, 7.3)	8.07	CH_3	14, 15	15
**17**	-	177.0	CO	-	-
**18**	-	177.5	CO	-	-

† These peaks are indistinguishable from each other.

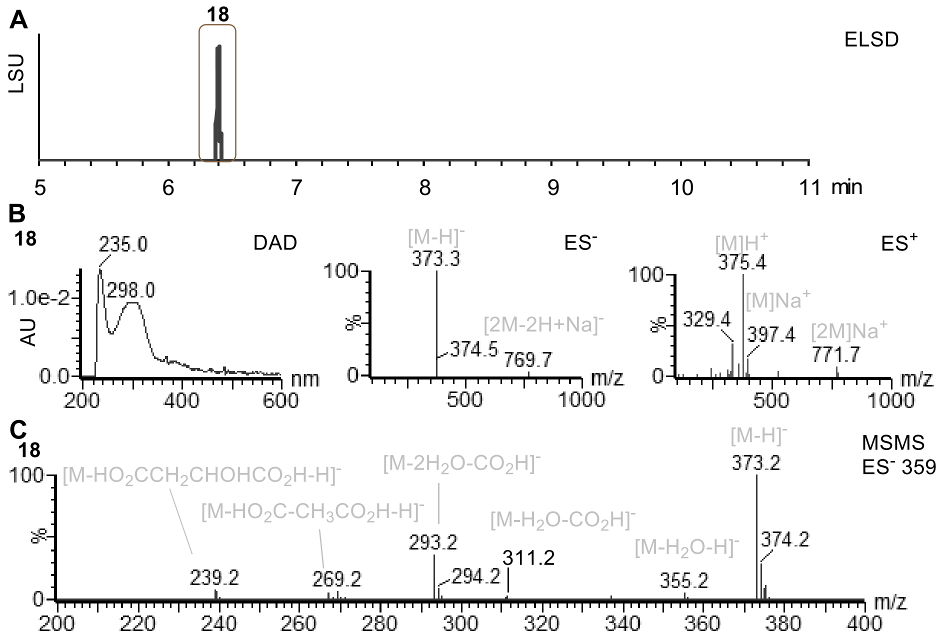

D

Elemental Composition Report

Single Mass Analysis
Tolerance = 20.0 PPM / DBE: min = -1.5, max = 50.0
Element prediction : Off
Number of isotopes peaks used for i-FIT = 3

Monoisotopic Mass, Even Electron Ions
395 formula(e) evaluated with 5 results within limits (all results (up to 1000) for each mass)
Elements Used:
C: 0-22 H: 0-35 N: 0-4 O: 0-8 S: 0-3

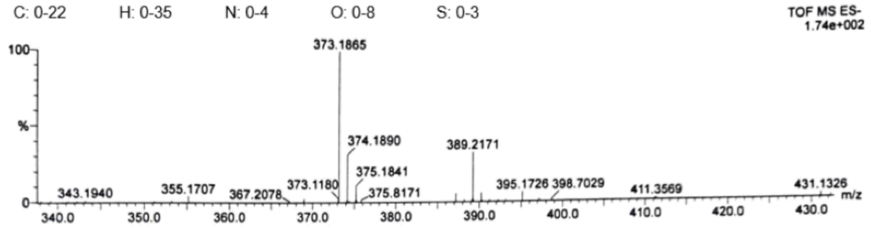

Minimum:				-1.5			
Maximum:		5.0	20.0	50.0			
Mass	Calc. Mass	mDa	PPM	DBE	i-FIT	i-FIT (Norm)	Formula
373.1865	373.1862	0.3	0.8	4.5	23.1	3.4	C18 H29 O8
	373.1876	-1.1	-2.9	9.5	22.9	3.2	C19 H25 N4 O4
	373.1837	2.8	7.5	8.5	19.9	0.2	C22 H29 O3 S
	373.1910	-4.5	-12.1	4.5	22.1	2.4	C16 H29 N4 O4 S
	373.1797	6.8	18.2	4.5	23.0	3.3	C17 H29 N2 O5 S

Figure S26 LCMS characterization and structure elucidation of **18**. **A**, ELSD trace of **18** (arbitrary units, smoothed in Excel); **B**, DAD, ES^- and ES^+ spectra of **18**; **C**, MSMS analysis of **18**; **D**, HRMS analysis.

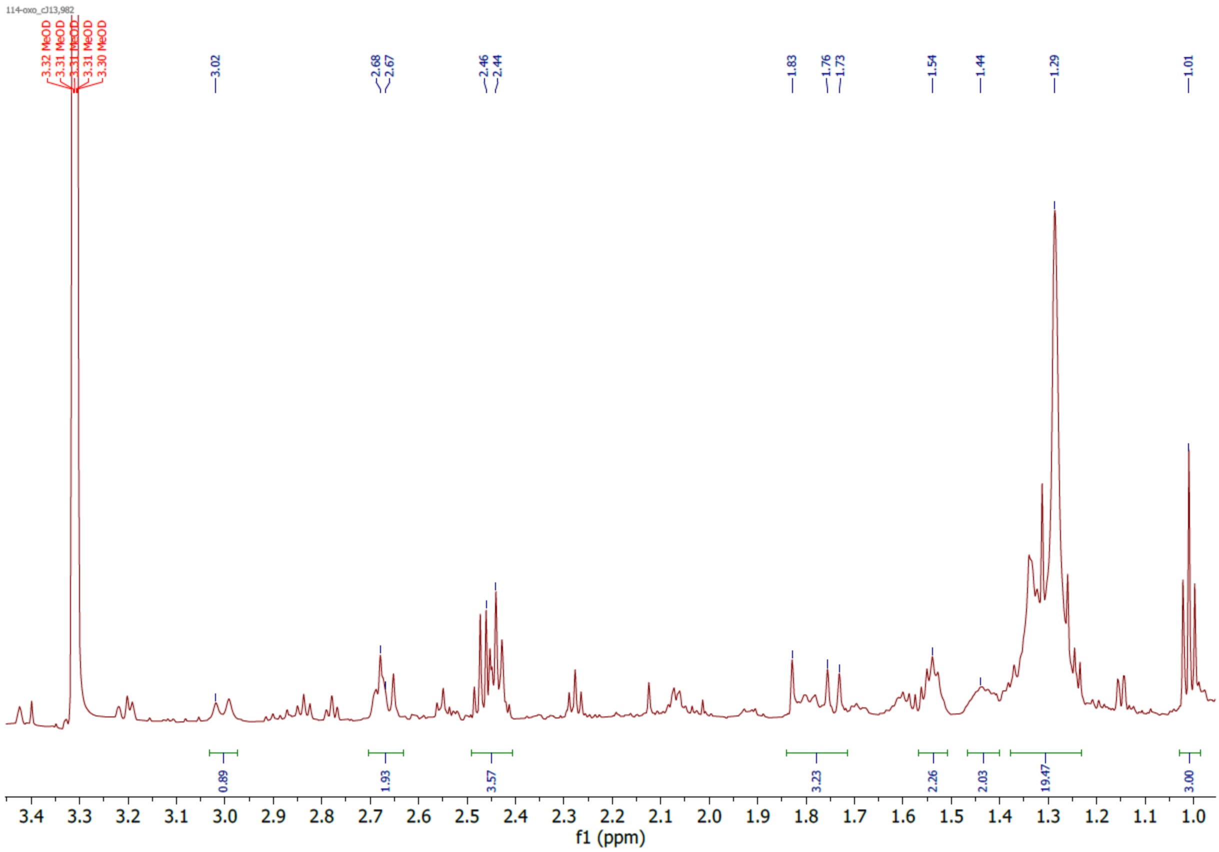

Figure S27 ^{1}H NMR of **18** in CD_3OD (600 MHz). See Table S20 for assignment.

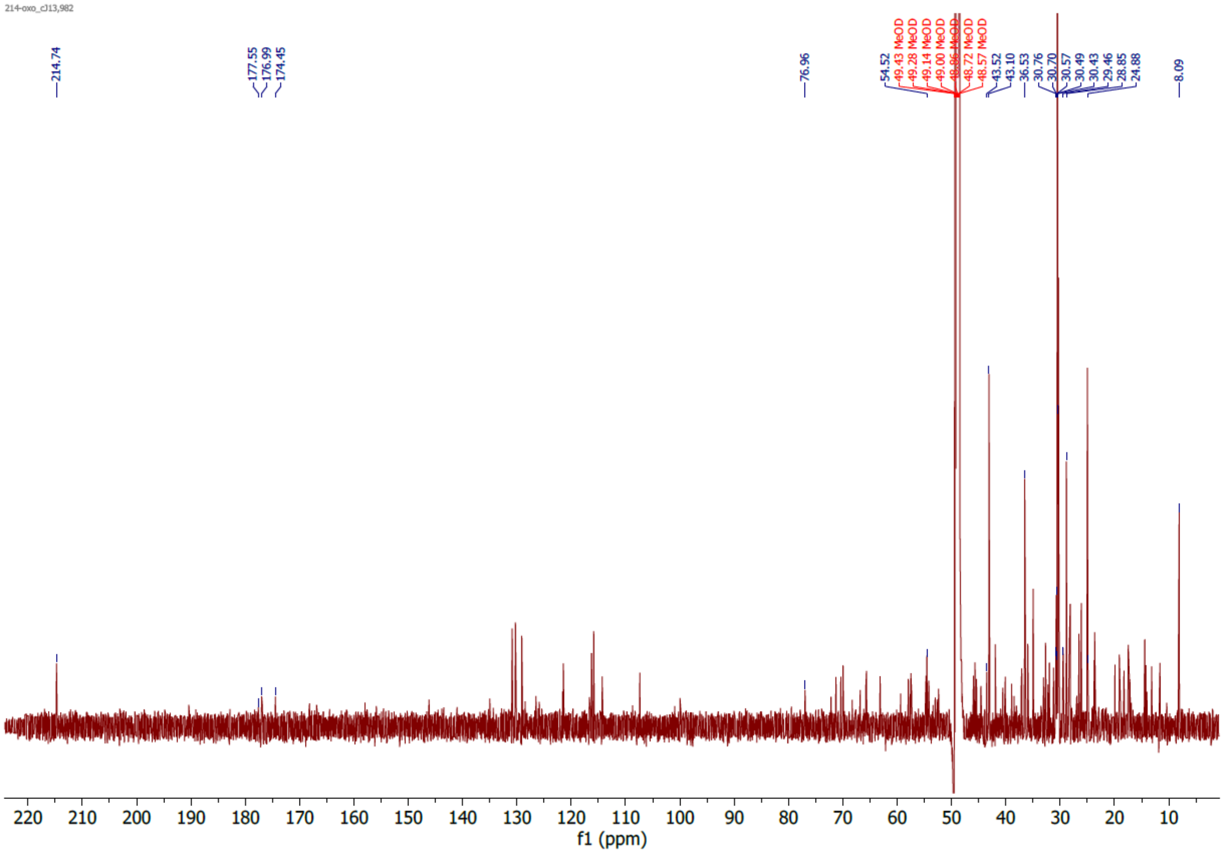

Figure S28 ^{13}C NMR of **18** in CD_3OD (150 MHz). See Table S20 for assignment.

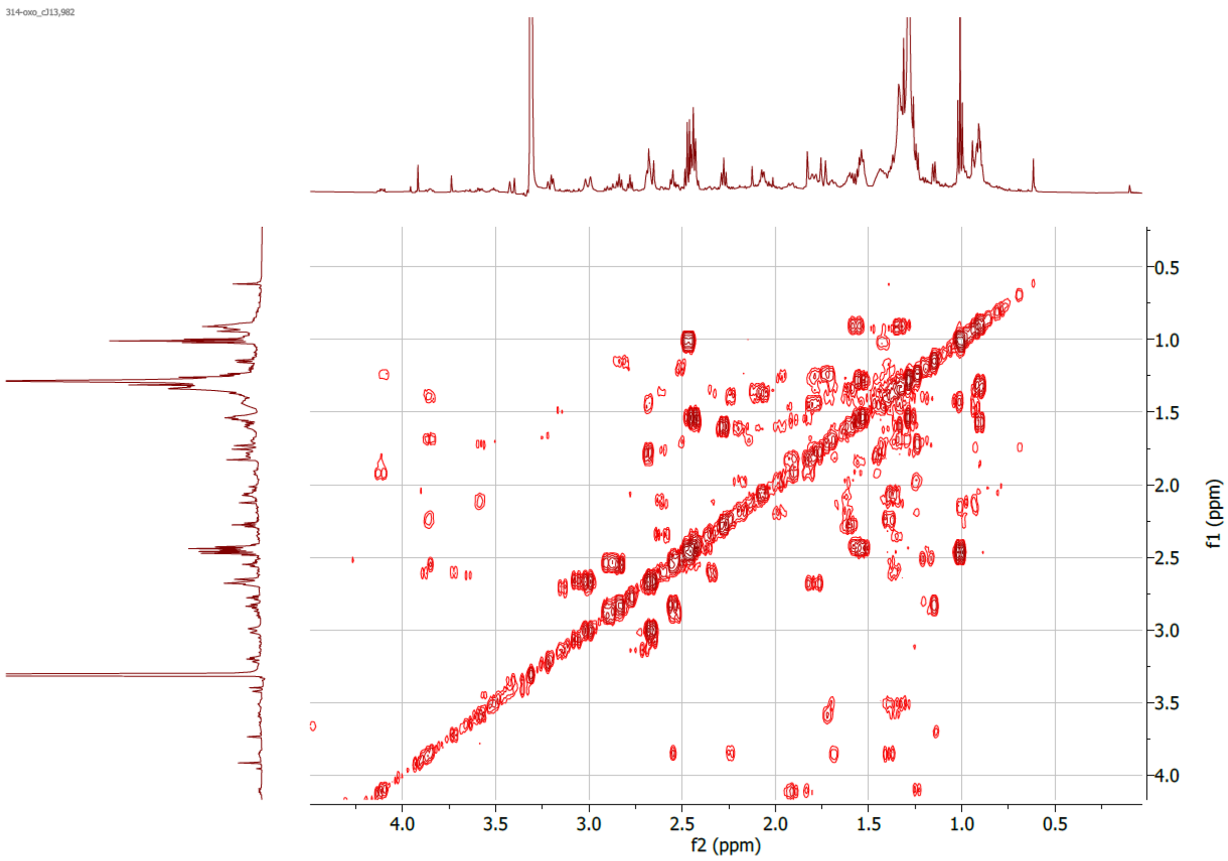

Figure S29 ^{1}H, ^{1}H COSY of **18** in CD_3OD.

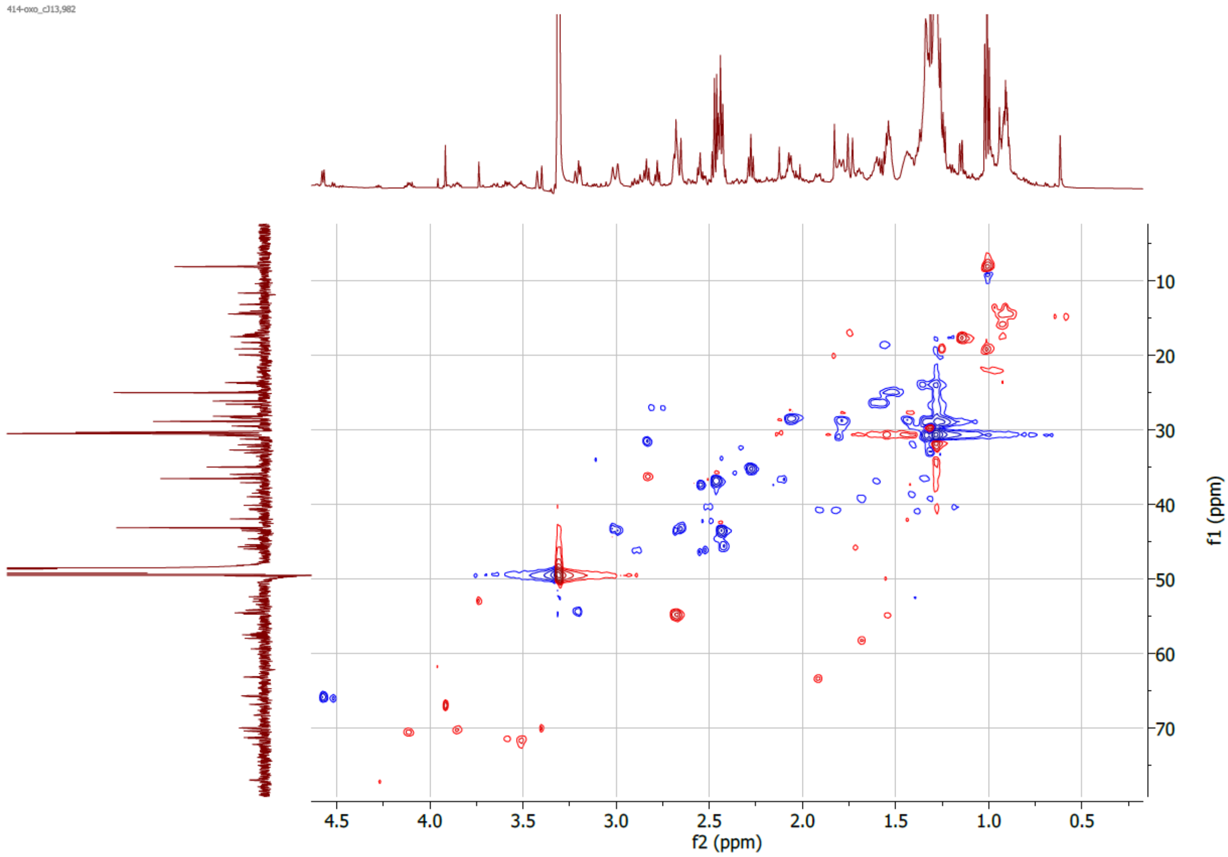

Figure S30 HSQC of **18** in CD_3OD.

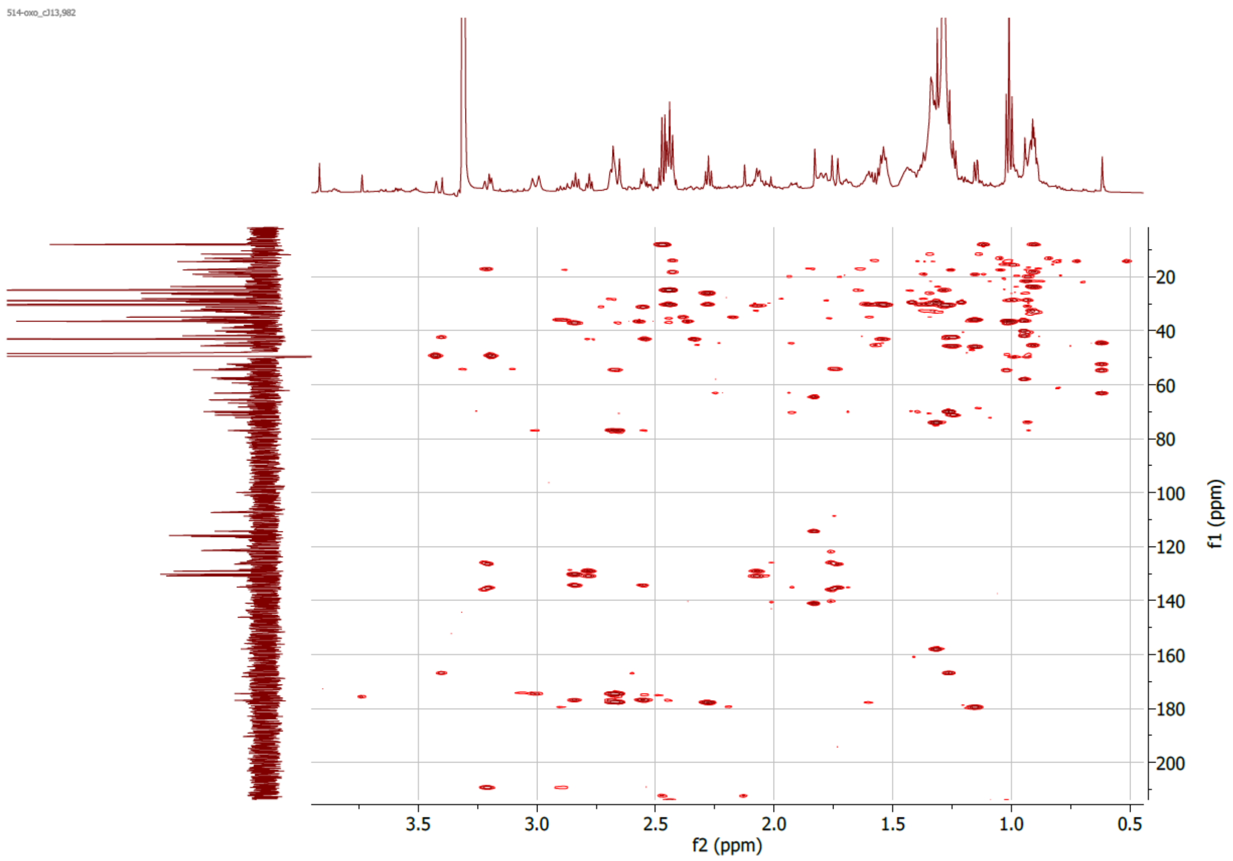

Figure S31 HMBC of **18** in CD_3OD.

6. EXPERIMENT 2 (*ctrR4* + *ctrR3* + *ctrACS* + *ctrR1*) *in vivo*

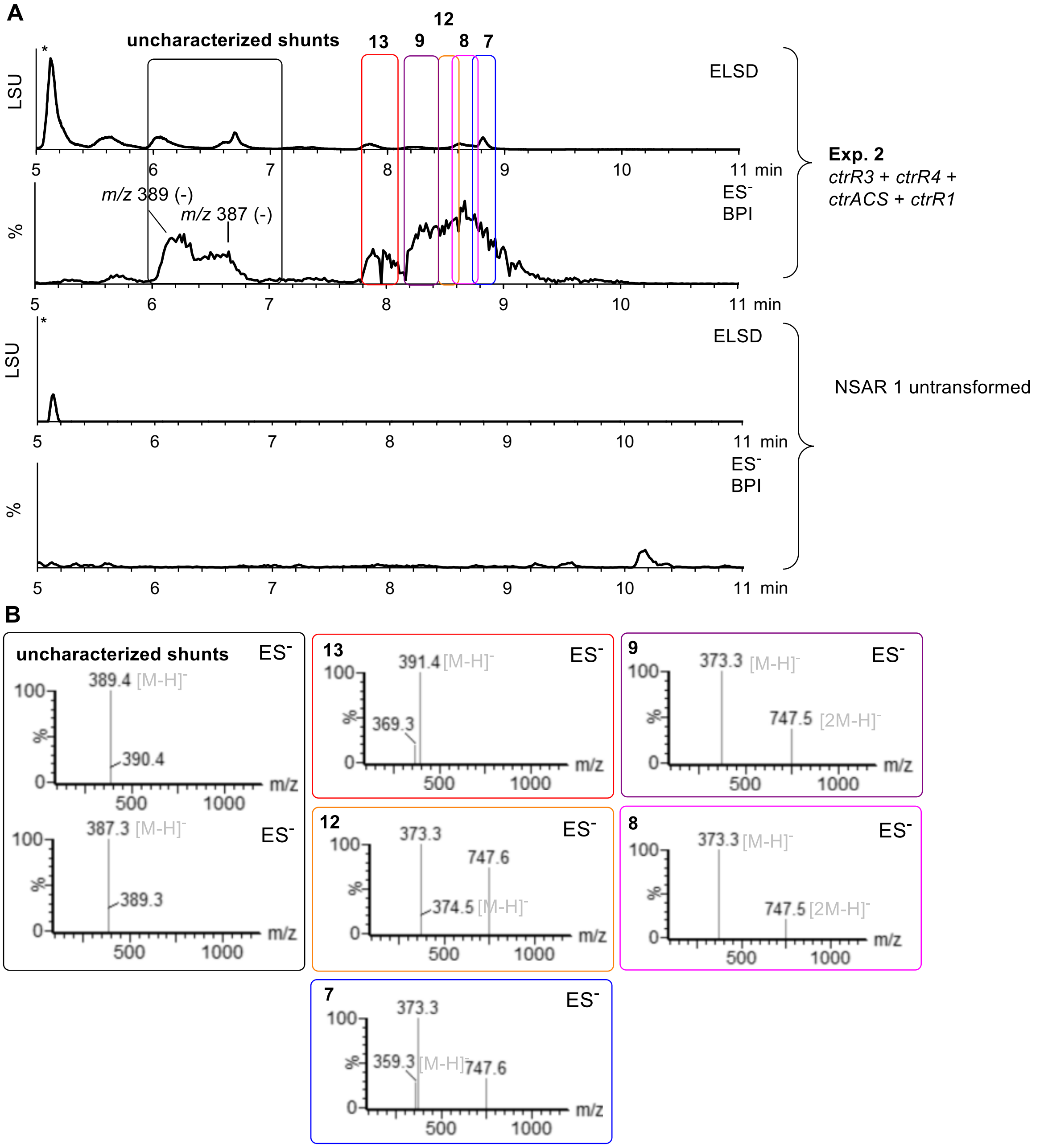

Figure S32 Heterologous expression of the structural genes of the *ctr* BGC: *ctrR4, ctrR3, ctrACS,* and *ctrR1*. **A**, LCMS chromatograms of extracts of *A. oryzae* NSAR1 transformant and untransformed control strain (arbitrary units, ELSD trace was smoothed in Excel); **B**, ES- spectra of compounds detected in *A. oryzae* NSAR1 transformants.

EXPERIMENT 2b (*ctrR4* + *ctrR3* + *ctrACS* + *ctrR1* +*ctrL2*) *in vivo*

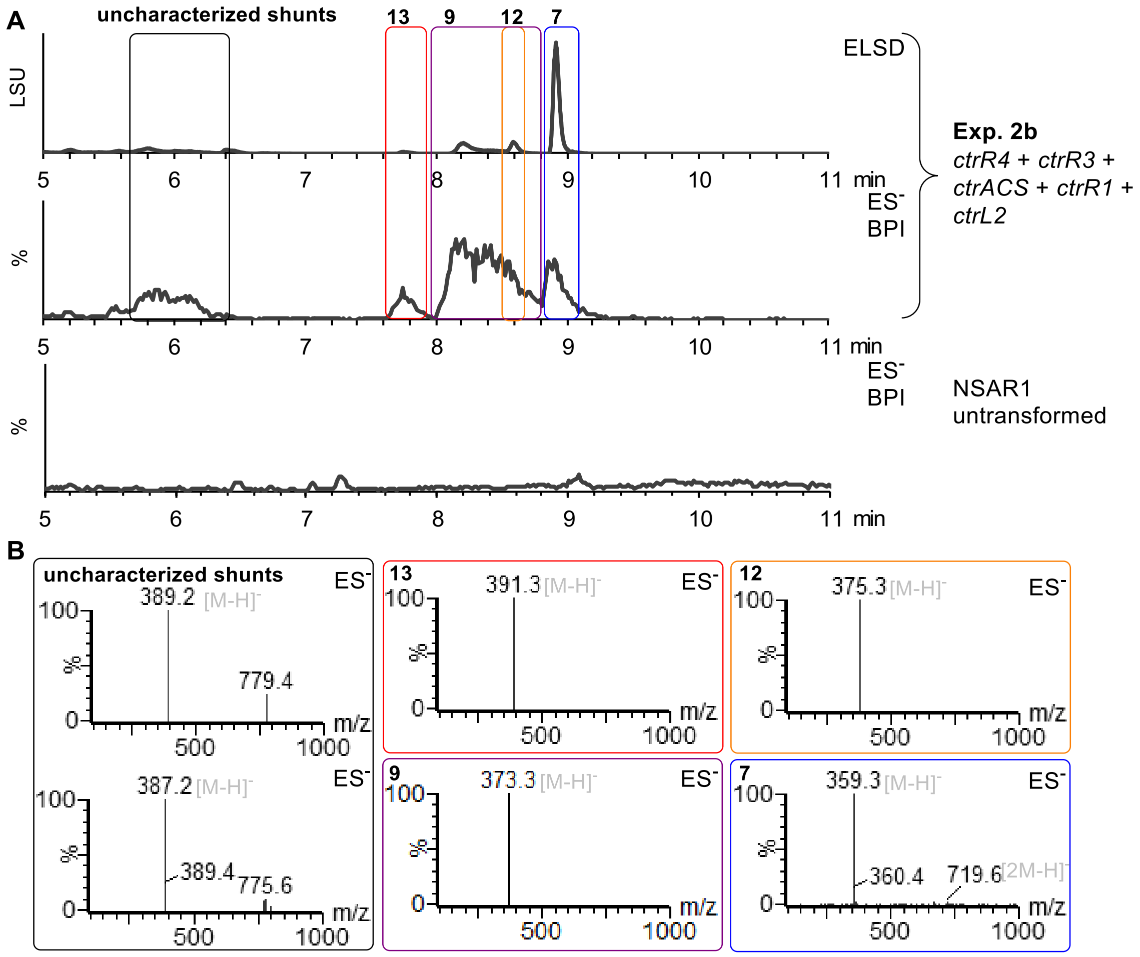

Figure S33 Heterologous expression of the structural genes of the *ctr* BGC: *ctrR4, ctrR3, ctrACS, ctrR1* and *ctrL2*. **A**, LCMS chromatograms of extracts of *A. oryzae* NSAR1 transformant and untransformed control strain (arbitrary units, ELSD trace was smoothed in Excel); **B**, ES- spectra of compounds detected in *A. oryzae* NSAR1 transformants.

6.1 Chacterisation of 4-Hydroxy CJ-13,982, 12

Table S21 Chemical shifts of **12** in DMSO-d6 (600 MHz).

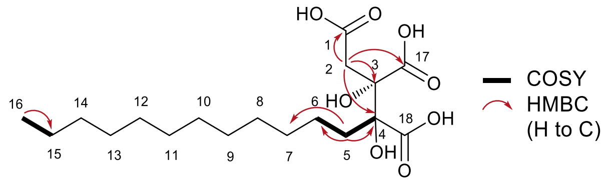

12

Chemical Formula: $C_{18}H_{32}O_8$
Exact Mass: 376.21

Pos.	δ_H/ ppm (mult, *J* in Hz)	δ_C/ ppm	C-type	HMBC H to C	COSY
**1**	-	174.8	CO	-	-
**2a**	3.13 (d, 17.7)	43.4	CH_2	1, 17	2b
**2b**	2.41 (d, 17.7)	43.4	CH_2	1, 3, 4, 17	2a
**3**	-	94.5	C	-	-
**4**	-	94.5	C	-	-
**5a**	1.81 (m)	27.6	CH_2	4	5b, 6
**5b**	1.50 (m)	27.6	CH_2	4, 18	5a, 6
**6**	1.3 (m)	31.8	CH_2	7-14	7-14
**7†**	1.3 (m)	32.0	CH_2	7-14	7-14
**8†**	1.3 (m)	32.1	CH_2	7-14	7-14
**9†**	1.3 (m)	32.1	CH_2	7-14	7-14
**10†**	1.3 (m)	32.2	CH_2	7-14	7-14
**11†**	1.3 (m)	32.2	CH_2	7-14	7-14
**12†**	1.3 (m)	32.2	CH_2	7-14	7-14
**13†**	1.3 (m)	32.4	CH_2	7-14	7-14
**14**	1.3 (m)	34.4	CH_2	7-14	7-14
**15**	1.3 (m)	25.2	CH_2	14	7-14
**16**	0.86 (t, 6.8)	17.1	CH_3	14, 15	14, 15
**17**	-	176.8	CO	-	-
**18**	-	172.4	CO	-	-

† These signals are indistinguishable from each other.

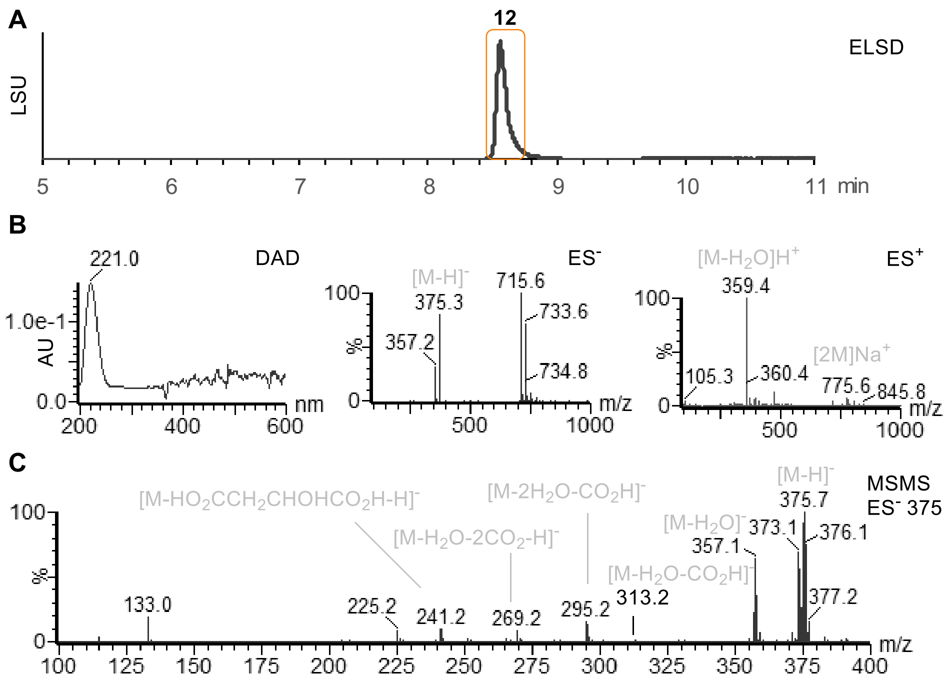

D

Elemental Composition Report

Single Mass Analysis
Tolerance = 20.0 PPM / DBE: min = -1.5, max = 50.0
Element prediction : Off
Number of isotopes peaks used for i-FIT = 3

Monoisotopic Mass, Even Electron Ions
75 formula(e) evaluated with 4 results within limits (all results (up to 1000) for each mass)
Elements Used:
C: 0-22 H: 0-50 O: 0-8 Na: 0-1

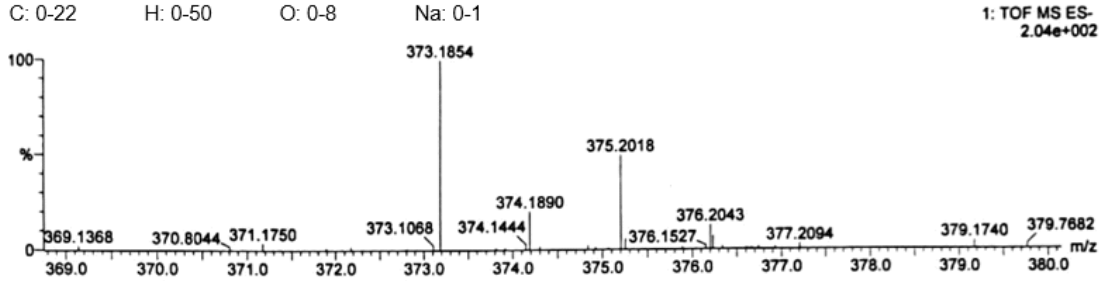

Minimum:				-1.5			
Maximum:		5.0	20.0	50.0			
Mass	Calc. Mass	mDa	PPM	DBE	i-FIT	i-FIT (Norm)	Formula
375.2018	375.2019	-0.1	-0.3	3.5	30.4	0.6	C18 H31 O8
	375.1995	2.3	6.1	0.5	31.0	1.3	C16 H32 O8 Na
	375.1960	5.8	15.5	12.5	31.9	2.1	C25 H27 O3
	375.2089	-7.1	-18.9	13.5	32.8	3.0	C27 H28 Na

Figure S34 LCMS characterization and structure elucidation of **12**. **A**, ELSD trace of **12** (arbitrary units, smoothed in Excel); **B**, DAD, ES- and ES+ spectra of **12**; **C**, MSMS data obtained for **12**; **D**, HRMS analysis and calculated molecular formula of **12**.

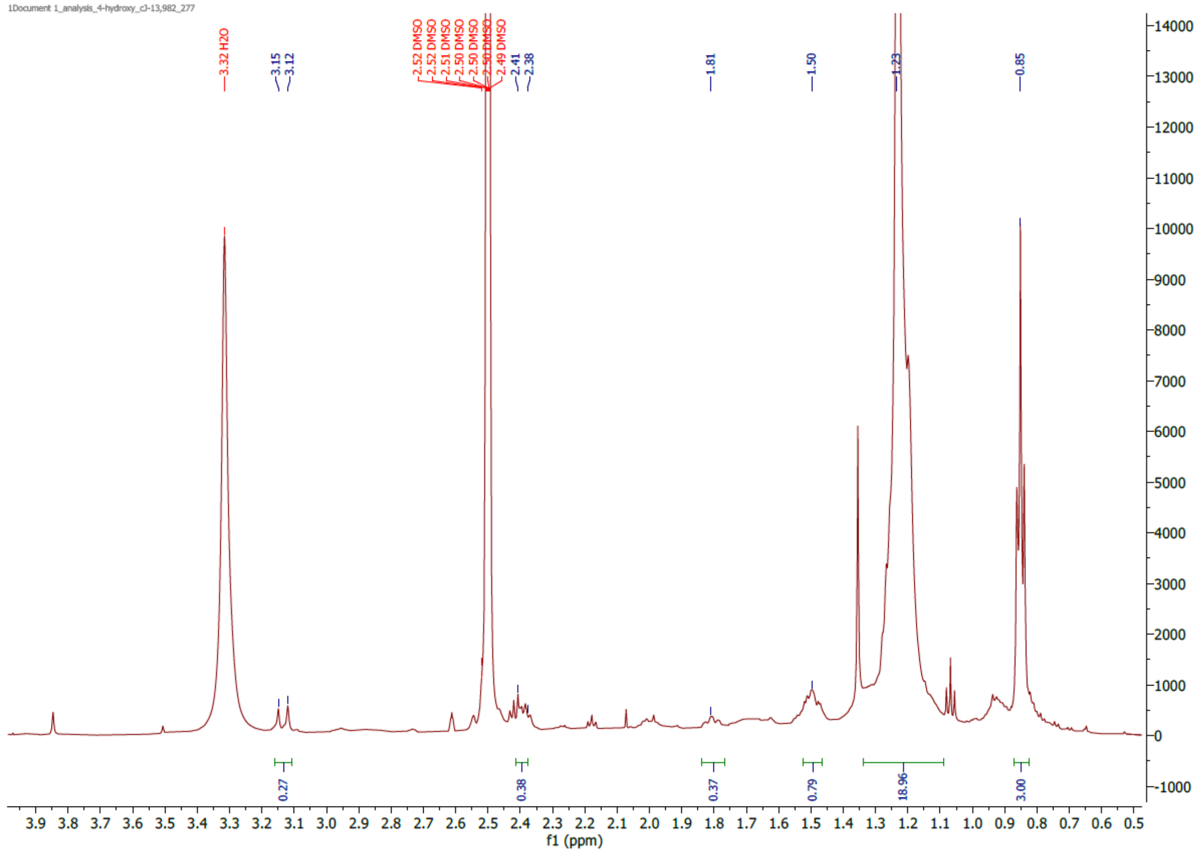

Figure S35 ^{1}H NMR of **12** in DMSO-d_6 (600 MHz). See Table S21 for assignment.

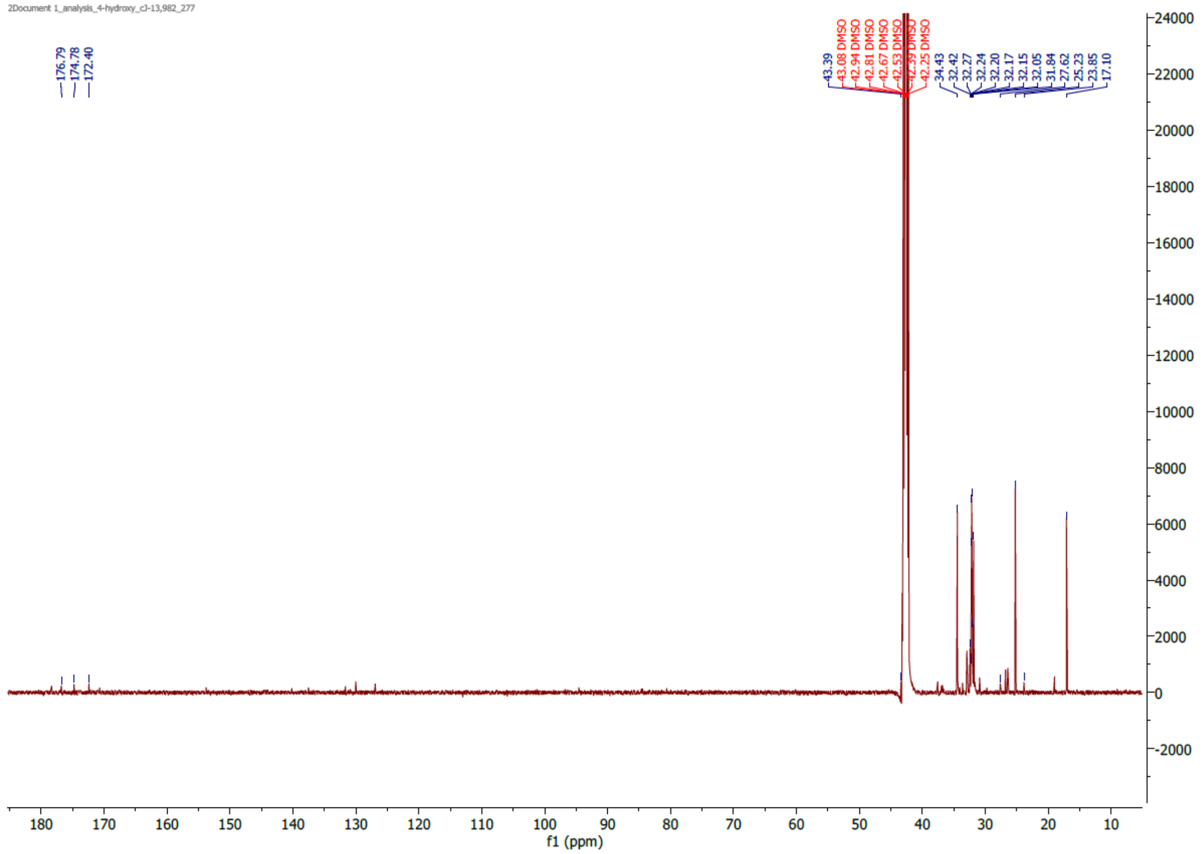

Figure S36 ^{13}C NMR of **12** in DMSO-d_6 (150 MHz). See Table S21 for assignment.

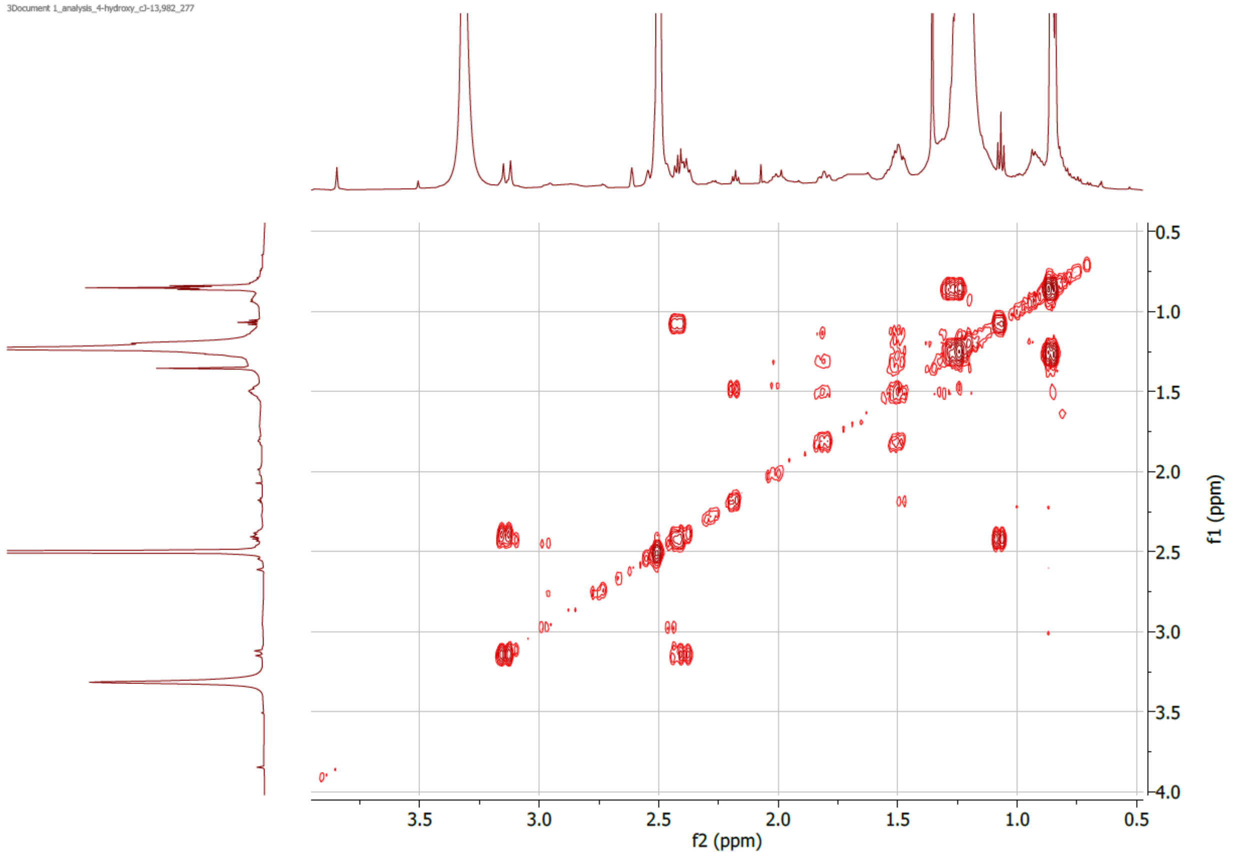

Figure S37 ^{1}H, ^{1}H COSY NMR of **12** in DMSO-d$_6$.

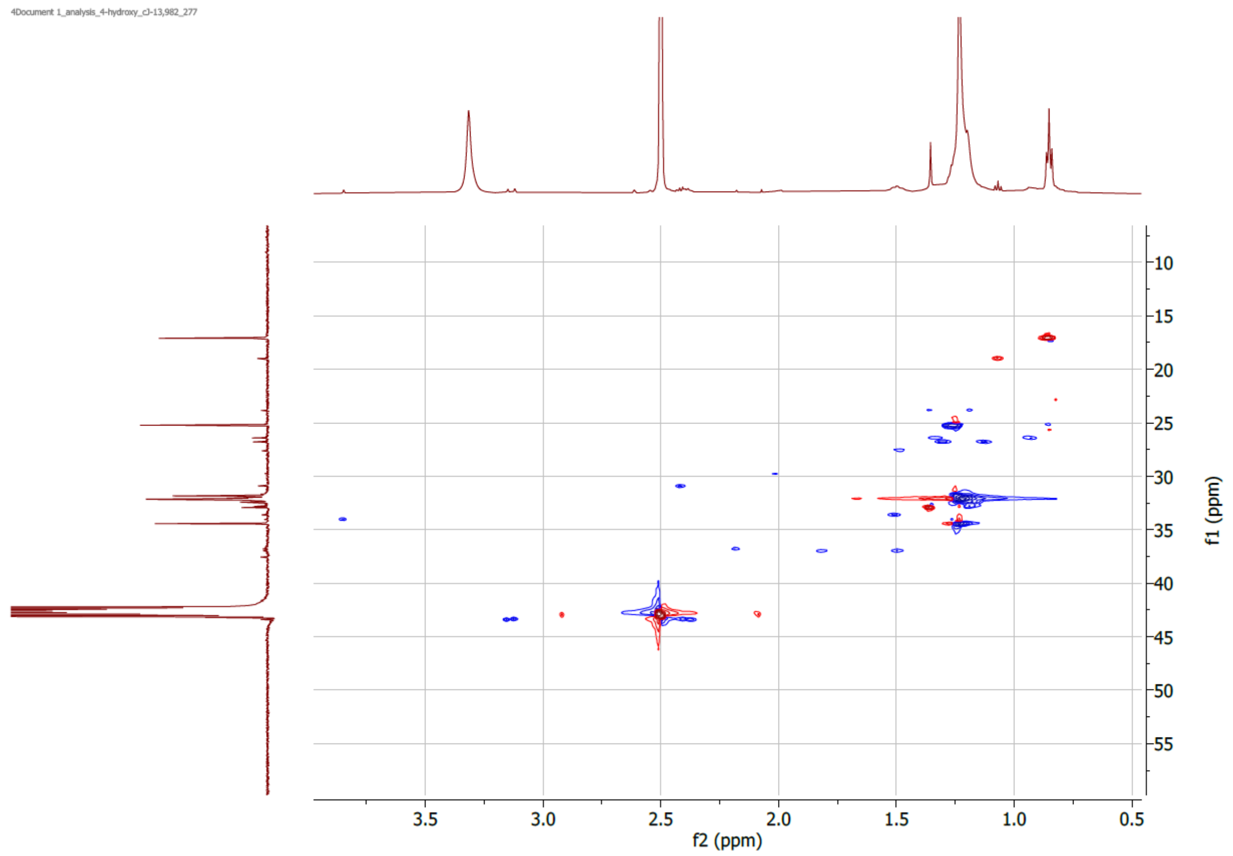

Figure S38 HSQC NMR of **12** in DMSO-d$_6$.

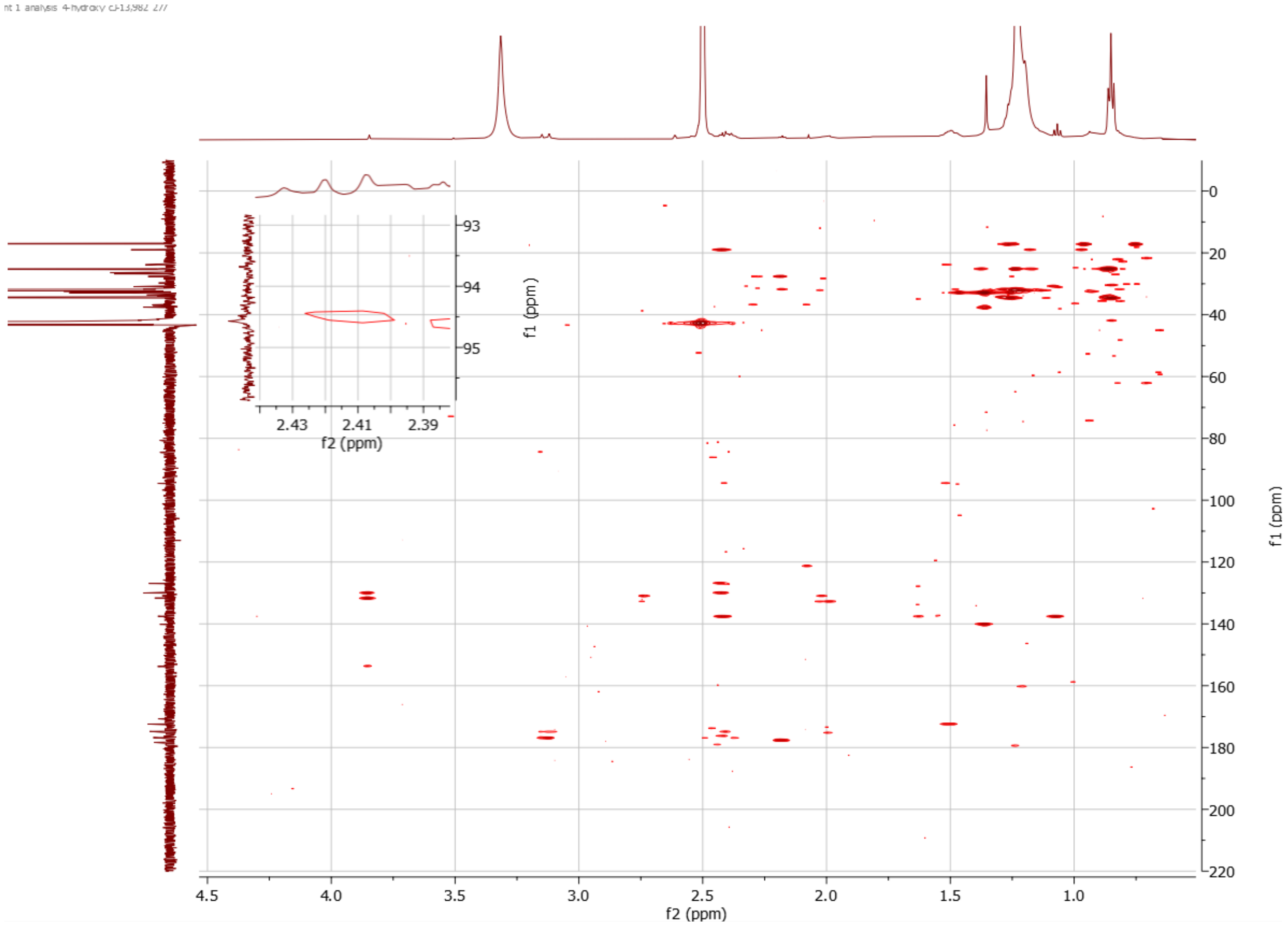

Figure S39 HMBC NMR of **12** in DMSO-d_6. Expansion window shows the HMBC correlation of H-2b to C-4.

6.2 Characterization of Cinatrin C_1 (8) and Cinatrin C_3 (9)

Table S22 Chemical shifts of partial NMR of **8** in DMSO-d_6 (600 MHz). n.d. = not detected.

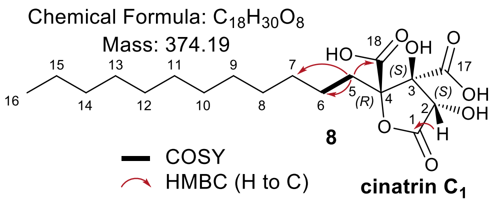

Experimentally observed						Compound **8** literature in ^{13}C in DMSO-d_6, ^{1}H in CD_3OD[20]	
**Pos.**	**δ_H/ ppm (mult, *J* in Hz)**	**δ_C/ ppm**	**C-type**	**HMBC H to C**	**COSY**	**δ_H/ ppm (mult, *J* in Hz)**	**δ_C/ ppm**
**1**	-	172.9	CO	-	-	-	173.6
**2**	4.72 (bs)	72.5	CH	1	-	4.69 (s)	73.1
**3**	-	n.d.	-	-	-	-	84.0
**4**	-	n.d.	-	-	-	-	86.5
**5a**	2.19 (app t, 7.4)	33.5	CH_2	6, 7, 18	6	2.14 (app.t, 8.4)	30.8
**5b**	n.d.	-	-	-	-	1.7 (m)	30.8
**6a**	1.5 (m)	24.5	CH_2	-	5	1.5 (m)	23.5
**6b**	n.d.	-	-	-	-	1.39 (m)	23.5
**7**	1.26 (m)	28.5	CH_2	-	-	1.28 (m)	28.7
**8†**	n.d.	-	-	-	-	1.28 (m)	28.8
**9†**	n.d.	-	-	-	-	1.28 (m)	29.2
**10†**	n.d.	-	-	-	-	1.28 (m)	29.0
**11†**	n.d.	-	-	-	-	1.28 (m)	29.0
**12†**	n.d.	-	-	-	-	1.28 (m)	29.0
**13†**	n.d.	-	-	-	-	1.28 (m)	28.7
**14†**	n.d.	-	-	-	-	1.28 (m)	31.3
**15†**	n.d.	-	-	-	-	1.28 (m)	22.1
**16†**	n.d.	-	-	-	-	0.89 (t, 6.9)	13.9
**17**	-	n.d.	CO	-	-	-	170.4
**18**	-	174.3	CO	-	-	-	170.6

† These signals are indistinguishable from cinatrin C_3 signals.

Table S23 Chemical shifts of **9** in DMSO-d_6 (600 MHz).

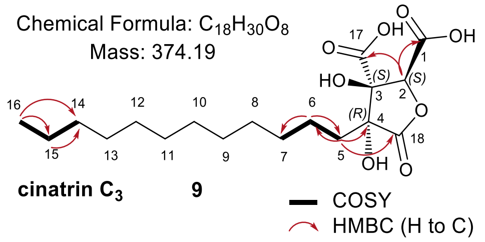

Experimentally observed						Compound **9** literature in DMSO-d_6[21]	
**Pos.**	**δ_H/ ppm (mult, *J* in Hz)**	**δ_C/ ppm**	**C-type**	**HMBC H to C**	**COSY**	**δ_H/ ppm (mult, *J* in Hz)**	**δ_C/ ppm**
**1**	-	167.6	CO	-	-	-	167.5
**2**	5.21 (s)	80.4	CH	1, 17	-	5.32 (s)	79.5
**3**	-	78.2	C	-	-	6.25 (br, 3-OH)	81.3
**4**	-	78.2	C	-	-	-	78.7
**5**	1.61 (m)	30.5	CH_2	4, 6, 7, 18	6	1.69 (m)	30.5
**6**	1.32 - 1.38 (m)	20.9	CH_2	5, 7	7, 5	1.41 (m)	21.0
**7**	1.25 (m)	29.7	CH_2	8-13	8-14	1.24 (m)	29.6
**8†**	1.25 (m)	28.7	CH_2	7-14	7-14	1.24 (m)	28.9
**9†**	1.25 (m)	29.0	CH_2	7-14	7-14	1.24 (m)	29.0
**10†**	1.25 (m)	29.0	CH_2	7-14	7-14	1.24 (m)	29.0
**11†**	1.25 (m)	29.0	CH_2	7-14	7-14	1.24 (m)	29.0
**12†**	1.25 (m)	29.1	CH_2	7-14	7-14	1.24 (m)	29.0
**13†**	1.25 (m)	29.1	CH_2	7-14	7-14	1.24 (m)	28.7
**14**	1.25 (m)	31.3	CH_2	15	7-14	1.24 (m)	31.2
**15**	1.26 (m)	22.1	CH_2	14, 16	14	1.24 (m)	22.0
**16**	0.85 (t, 6.9)	14.0	CH_3	14, 15	15	0.86 (t)	13.9
**17**	-	170.9	CO	-	-	-	170.4
**18**	-	174.9	CO	-	-	-	174.6

† These signals are indistinguishable from each other.

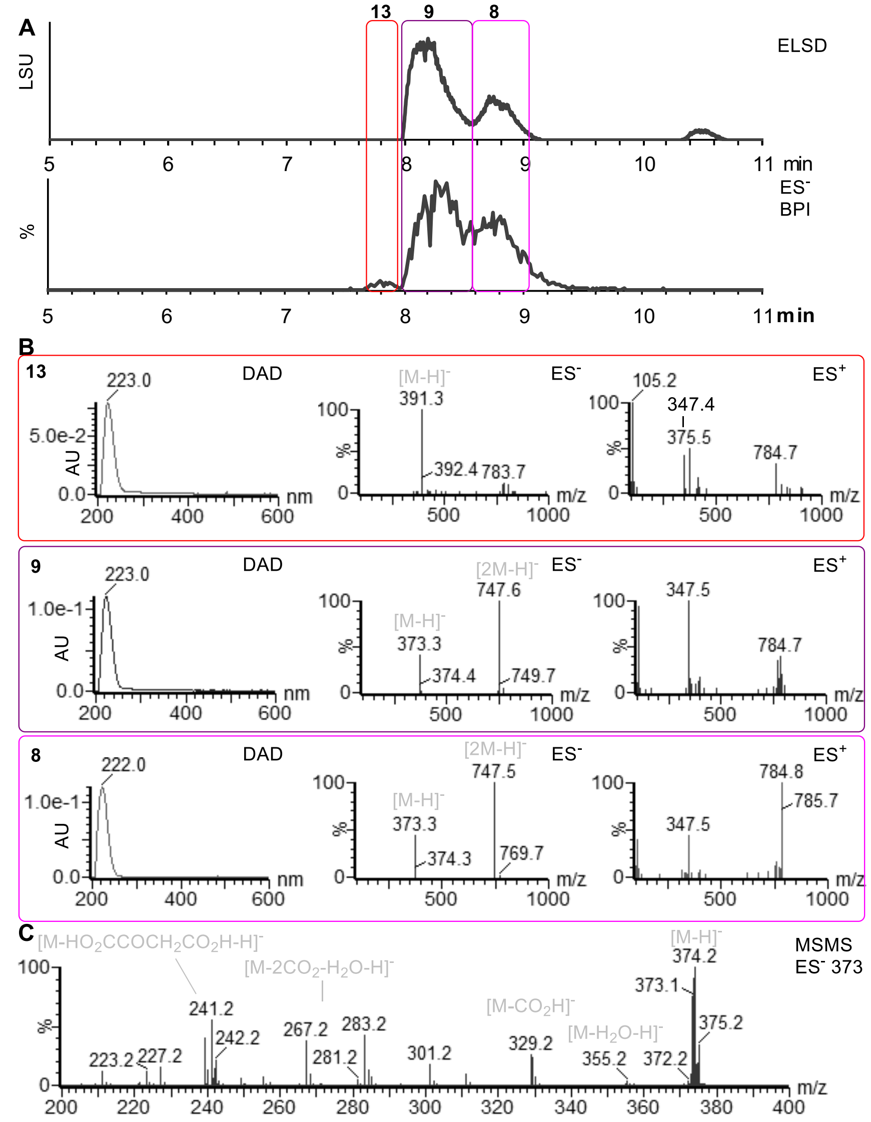

D

Elemental Composition Report

Single Mass Analysis
Tolerance = 20.0 PPM / DBE: min = -1.5, max = 50.0
Element prediction : Off
Number of isotopes peaks used for i-FIT = 3

Monoisotopic Mass, Even Electron Ions
74 formula(e) evaluated with 4 results within limits (all results (up to 1000) for each mass)
Elements Used:
C: 0-22 H: 0-50 O: 0-8 Na: 0-1

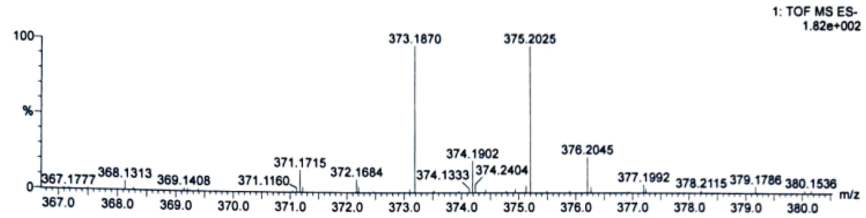

Mass	Calc. Mass	mDa	PPM	DBE	i-FIT	i-FIT (Norm)	Formula
Minimum:				-1.5			
Maximum:		5.0	20.0	50.0			
373.1870	373.1862	0.8	2.1	4.5	46.8	1.4	C18 H29 O8
	373.1838	3.2	8.6	1.5	47.1	1.7	C16 H30 O8 Na
	373.1932	-6.2	-16.6	14.5	46.1	0.8	C27 H26 Na
	373.1804	6.6	17.7	13.5	47.4	2.1	C25 H25 O3

Figure S40 LCMS characterization of **8** and **9**. **A**, ELSD and ES$^-$ traces of **13**, **8** and **9** (arbitrary units, ELSD smoothed in Excel); **B**, DAD, ES$^-$ and ES$^+$ spectra of **13**, **8** and **9**; **C**, MSMS data obtained for **8** and **9; D**, HRMS analysis and calculated molecular formula of **8** and **9**.

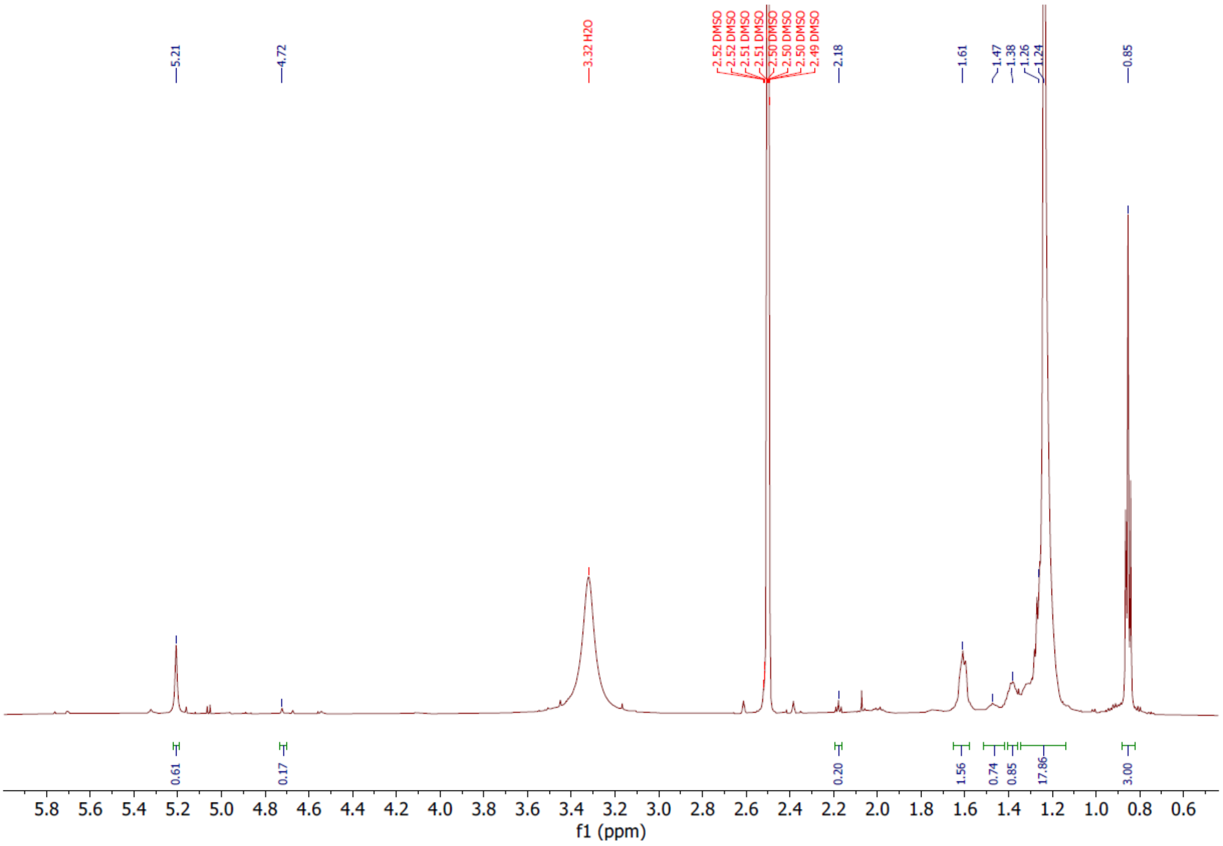

Figure S41 ^{1}H NMR of **8** and **9** in DMSO-d_6 (600 MHz). See Table S22 and Table S23 for assignments.

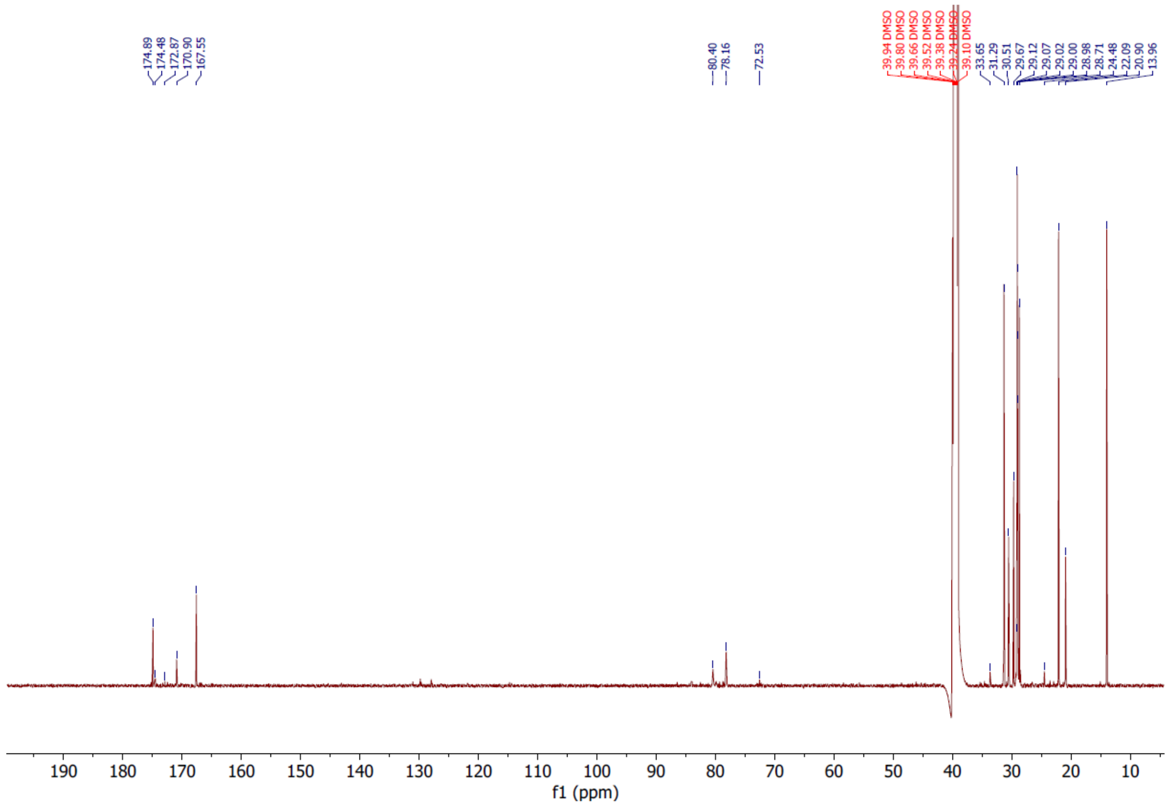

Figure S42 ^{13}C NMR of **8** and **9** in DMSO-d_6 (150 MHz). See Table S22 and Table S23 for assignments.

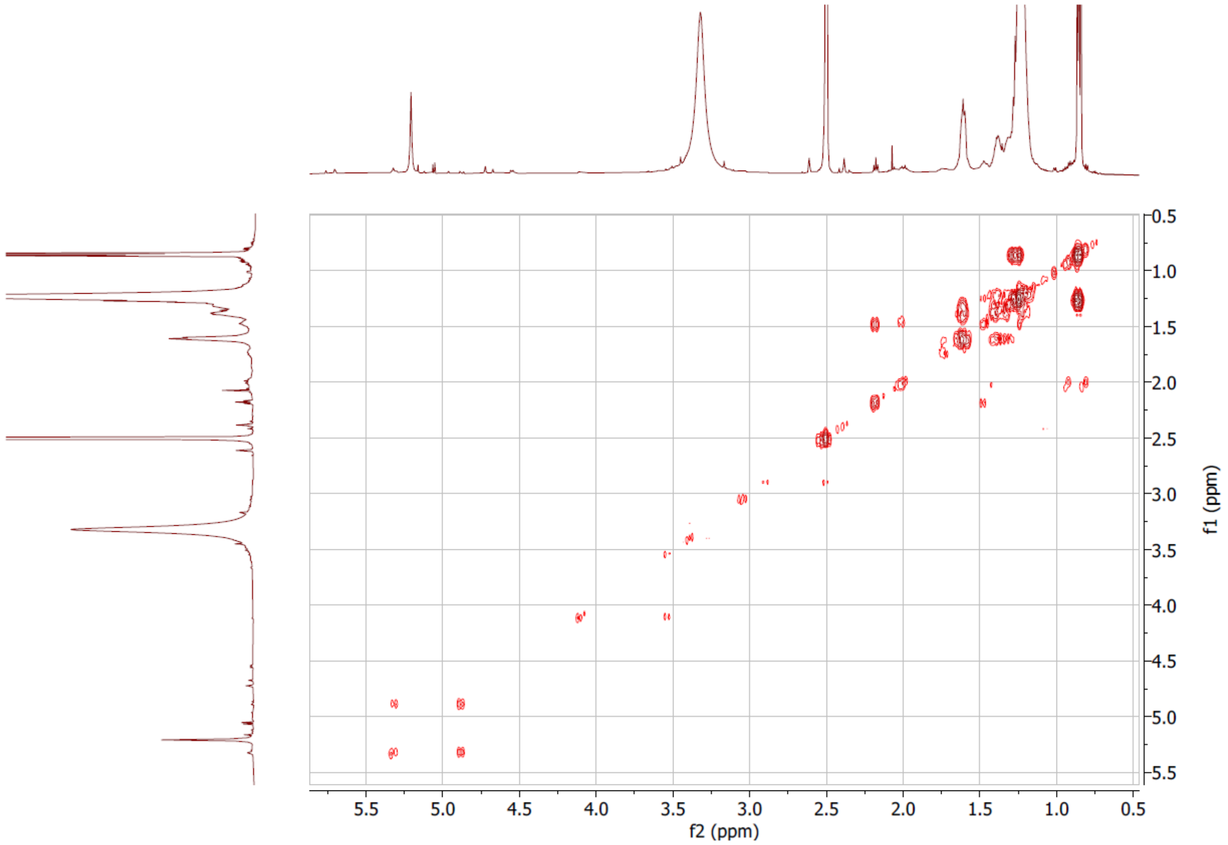

Figure S43 ^{1}H, ^{1}H COSY NMR of **8** and **9** in DMSO-d$_6$.

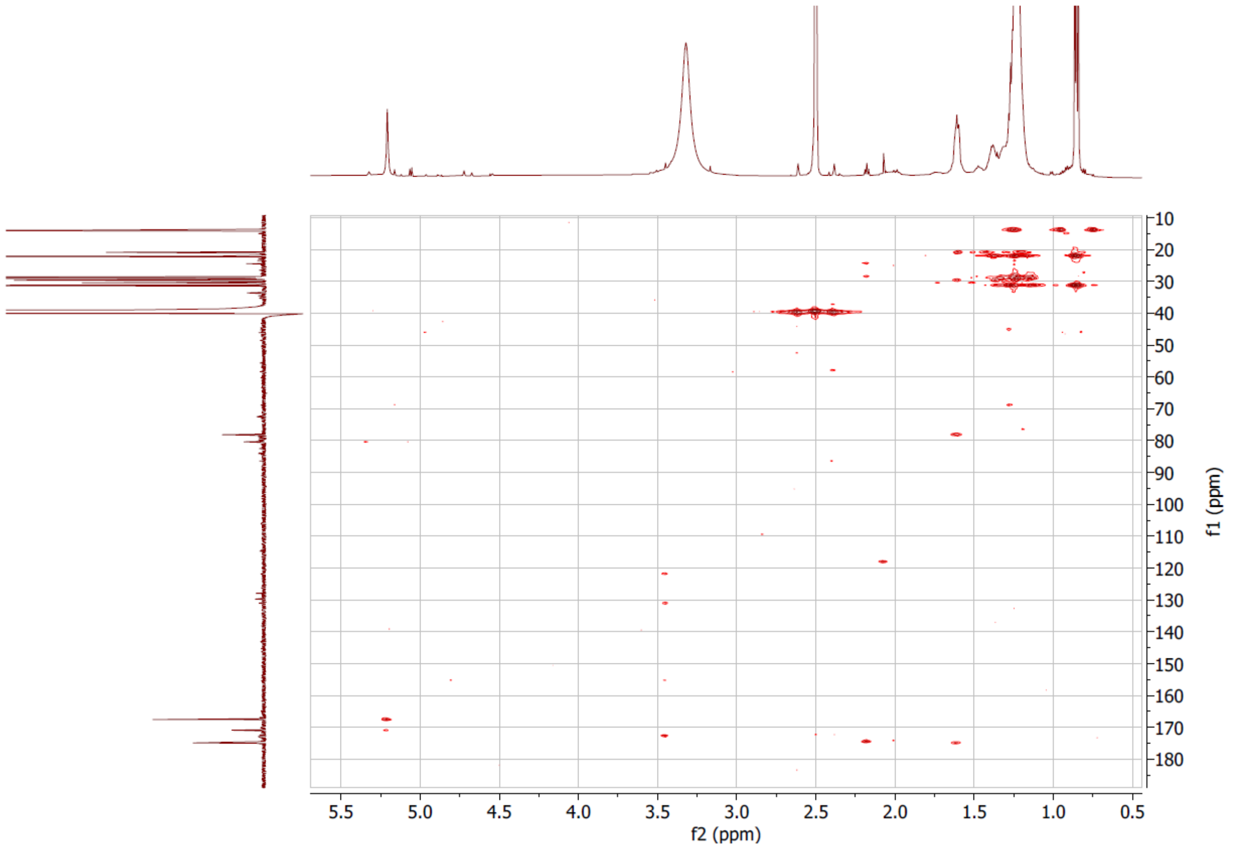

Figure S44 HMBC NMR of **8** and **9** in DMSO-d$_6$.

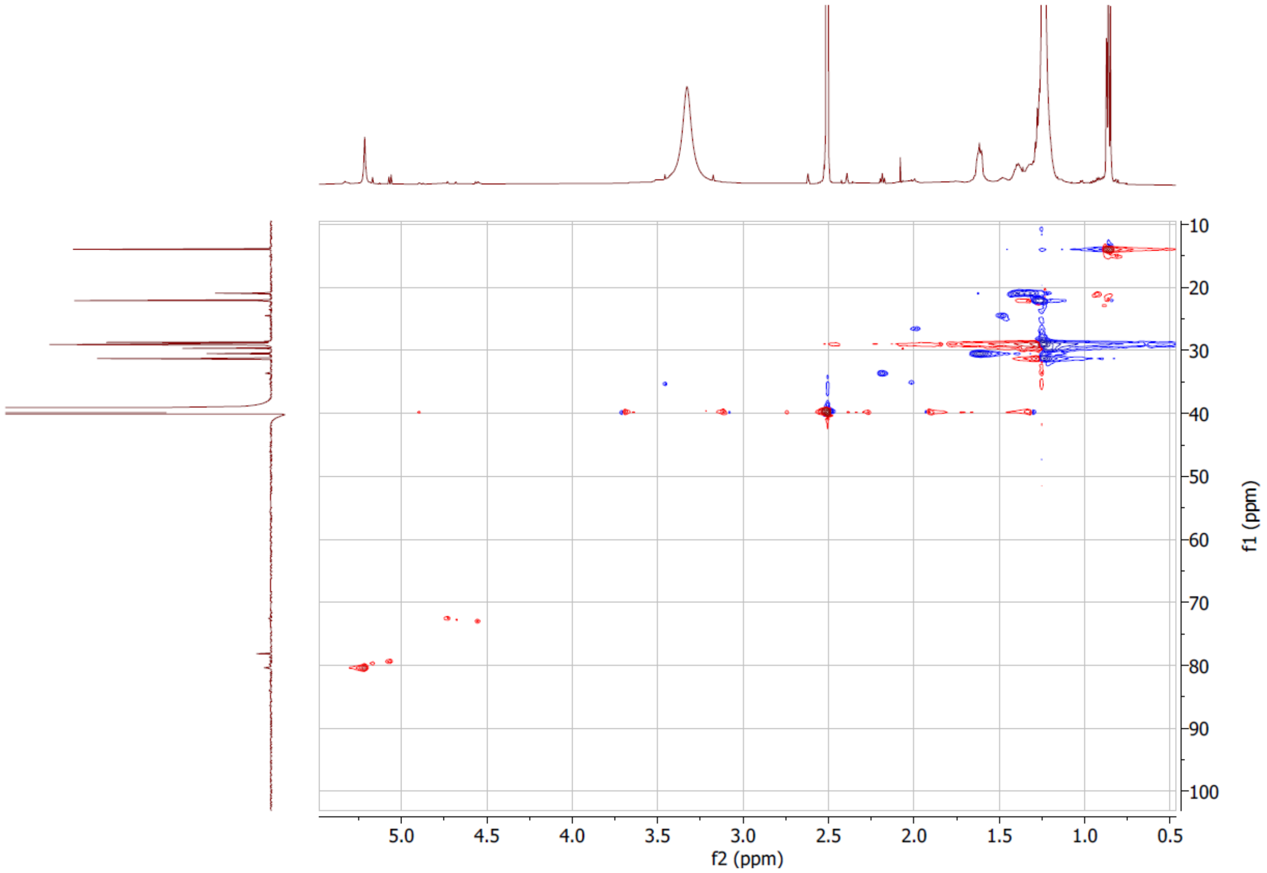

Figure S45 HSQC NMR of **8** and **9** in DMSO-d_6.

7. EXPERIMENT 3 (7 + CtrR1) *in vitro*

Incubation of purified Trx-His₆-CtrR1 with CJ-13,982 **7**, $FeSO_4$, α-ketoglutarate, and ascorbate at 28 °C for 2 h resulted in formation of **12** and **13** (Fig. S46A4), while omission of α-ketoglutarate abolished the reaction (Fig. S46A1). In reactions with purified His₆-CtrR1 sequential addition of 75 % of the amount of the initially added NHI oxygenase solution every 20 min over the course of the reaction, resulted in complete consumption of substrate CJ-13,982 **7** (Fig. S47).

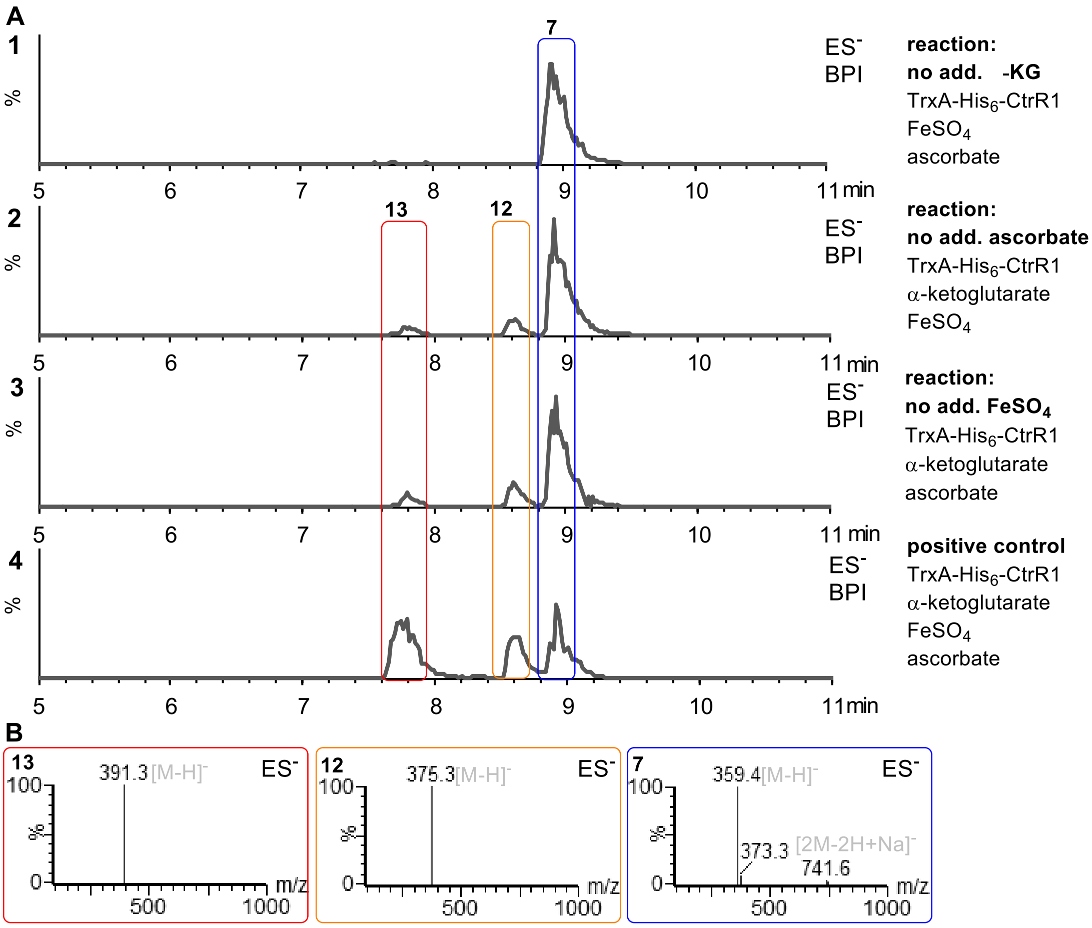

Figure S46 *In vitro* analysis of cofactor-dependency of TrxA-His₆-CtrR1. **A**, LCMS analysis of reactions containing all or a subset of additives (arbitrary units); **B**, ES⁻ spectra of substrate and products.

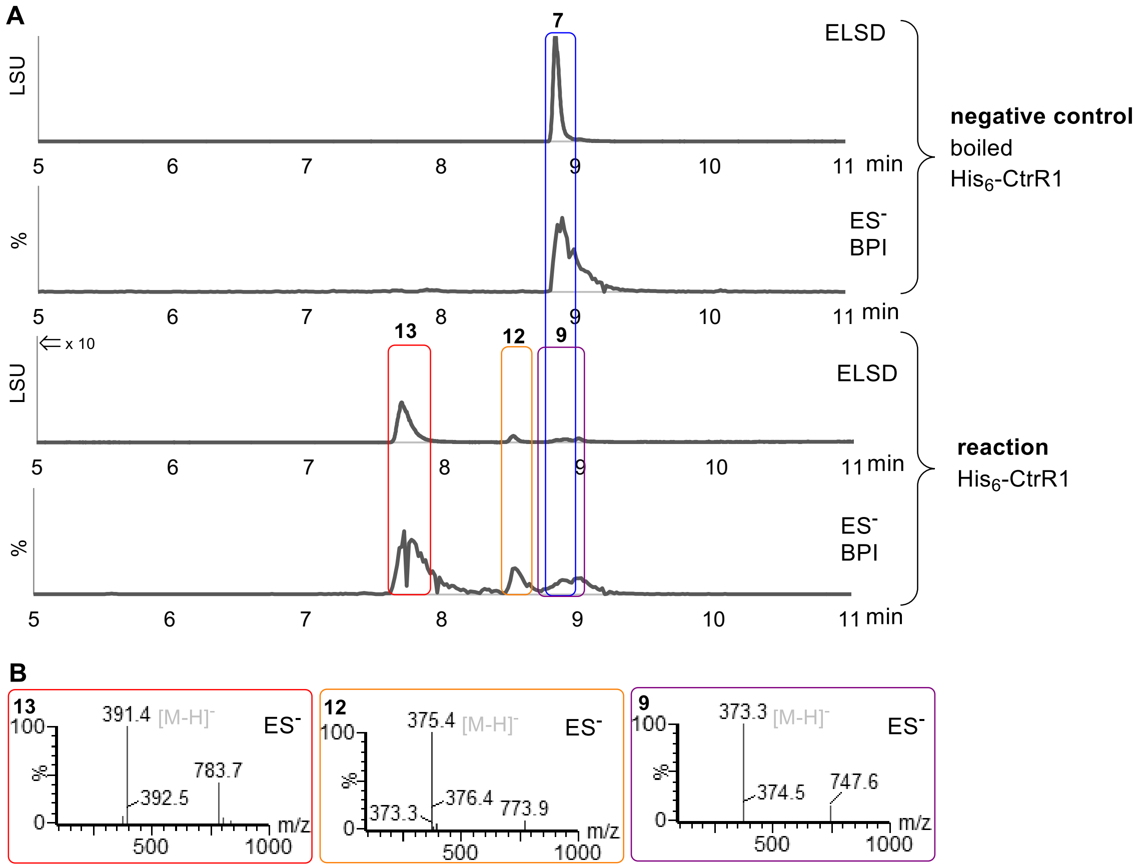

Figure S47 *In vitro* studies with recombinant His$_6$-CtrR1. **A**, LCMS analysis of *in vitro* assays with recombinant His$_6$-CtrR1 and boiled His$_6$-CtrR1 as control (arbitrary units, zoom is indicated by (←x) where applied, ELSD trace smoothed in Excel); **B**, ES$^-$ spectra of substrate and products.

8. EXPERIMENT 4 (7 + Mfr1) *in vitro*

Incubation of purified Trx-His$_6$-MfR1 with CJ-13,982 **7**, $FeSO_4$, α-ketoglutarate, and ascorbate at 28 °C for 2 h resulted in the formation of the same products as observed for CtrR1 (Fig. S48 compare to Fig. S46). In addition, another peak appeared at t_R = 6.8 min with *m/z* ES$^-$ = 407.3 Da (**21**, Fig. S48A1, B) corresponding to triply hydroxylated CJ-13,982 **7**. This peak was not visible in a reaction with no additional ascorbate or $FeSO_4$ (Fig. S48A3, 4) and is so-far unidentified.

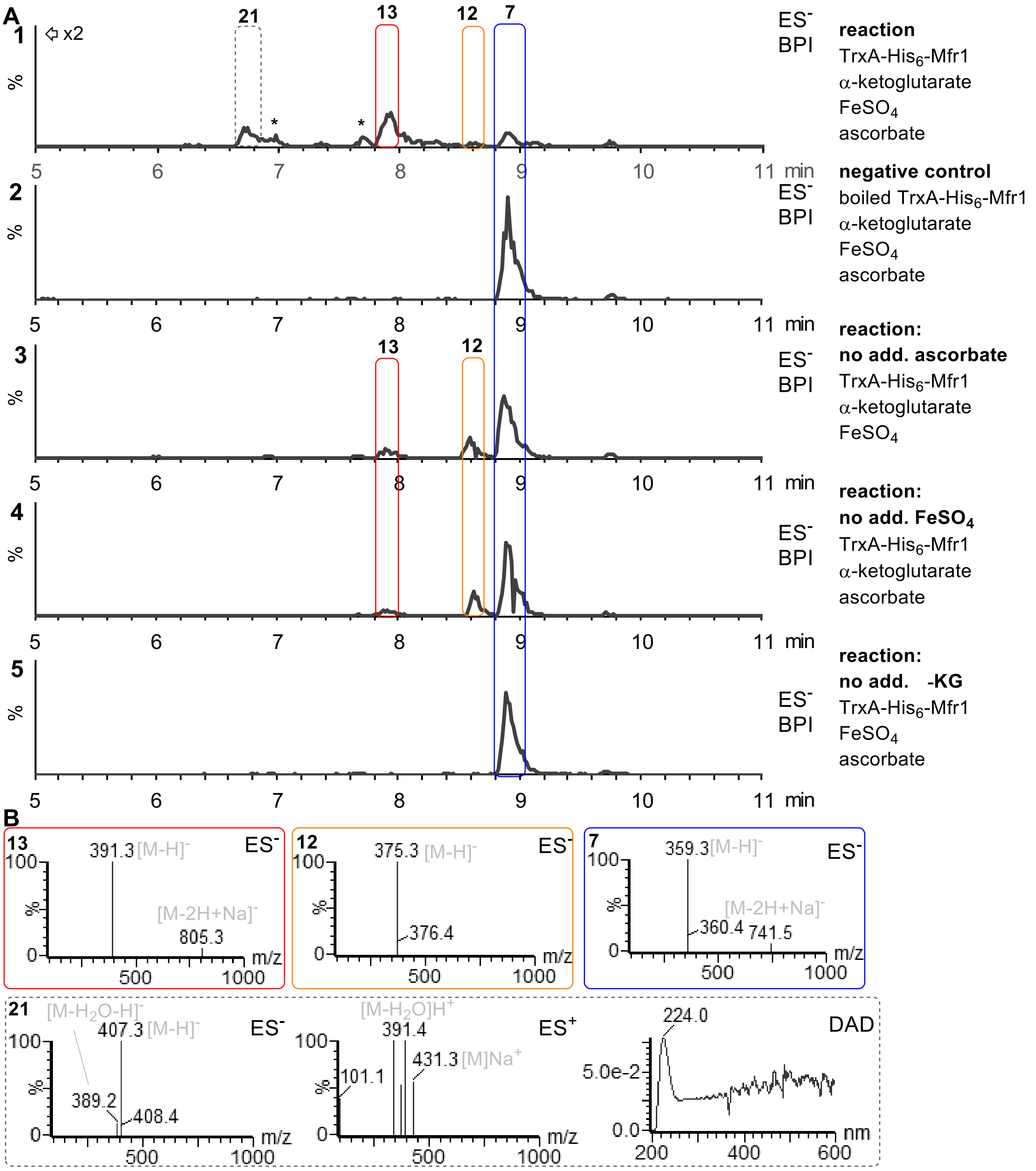

Figure S48 *In vitro* analysis of activity of TrxA-His$_6$-MfR1. **A**, LCMS analysis of reactions with TrxA-His$_6$-MfR1 and boiled control (arbitrary units, * = unrelated compounds, zoom is indicated by (←x) where applied); **B**, ES$^-$, ES$^+$, DAD of products. See Fig. S75 for predicted structures.

9. EXPERIMENT 5 (7 + MfR2) *in vitro*

In the presence of TrxA-His_6-Mfr2 with $FeSO_4$, α-ketoglutarate, and ascorbate at 28 °C for 2 h CJ-13,982 **7** was fully converted (Fig. S49, 1) and three new peaks appeared at t_R = 7.5 min with *m/z* ES^- = 387 Da (Fig. S50, corresponds to 5,6-epoxy-7-oxo-CJ-13,982 **25**); t_R = 6.9 min with *m/z* ES^- = 389 (Fig. S50, corresponds to 5-hydroxy-7-oxo-CJ-13,982 **24**); t_R = 6.5 min with *m/z* ES^- = 391 Da (Fig. S50, corresponds to 5,7-dihydroxy-CJ-13,982, **22**). In reactions that lacked additional ascorbate or $FeSO_4$ (Fig. S49, 3-4) the peak at t_R = 7.5 min with *m/z* ES^- = 387 Da was absent, but two other peaks were detected: t_R = 7.2 min with *m/z* ES^- = 375 Da (Fig. S50, identical to **14**) and t_R = 7.6 min with *m/z* ES^- = 373 Da (Fig. S50, corresponds to 7-oxo-CJ-13,982 **23**). See Fig. S50 for MS spectra of corresponding peaks. Attempts to purify the produced compounds for characterization were not successful, except in the case of **14**. In a large scale *in vitro* reaction (Fig. S51) using TrxA-His_6-MfR2 and approximately 5 mg CJ-13,982 **7**, compound **14** (0.6 mg) was isolated and identified as 7-hydroxy-CJ-13,982 by NMR and HRMS analysis (see section 9.1).

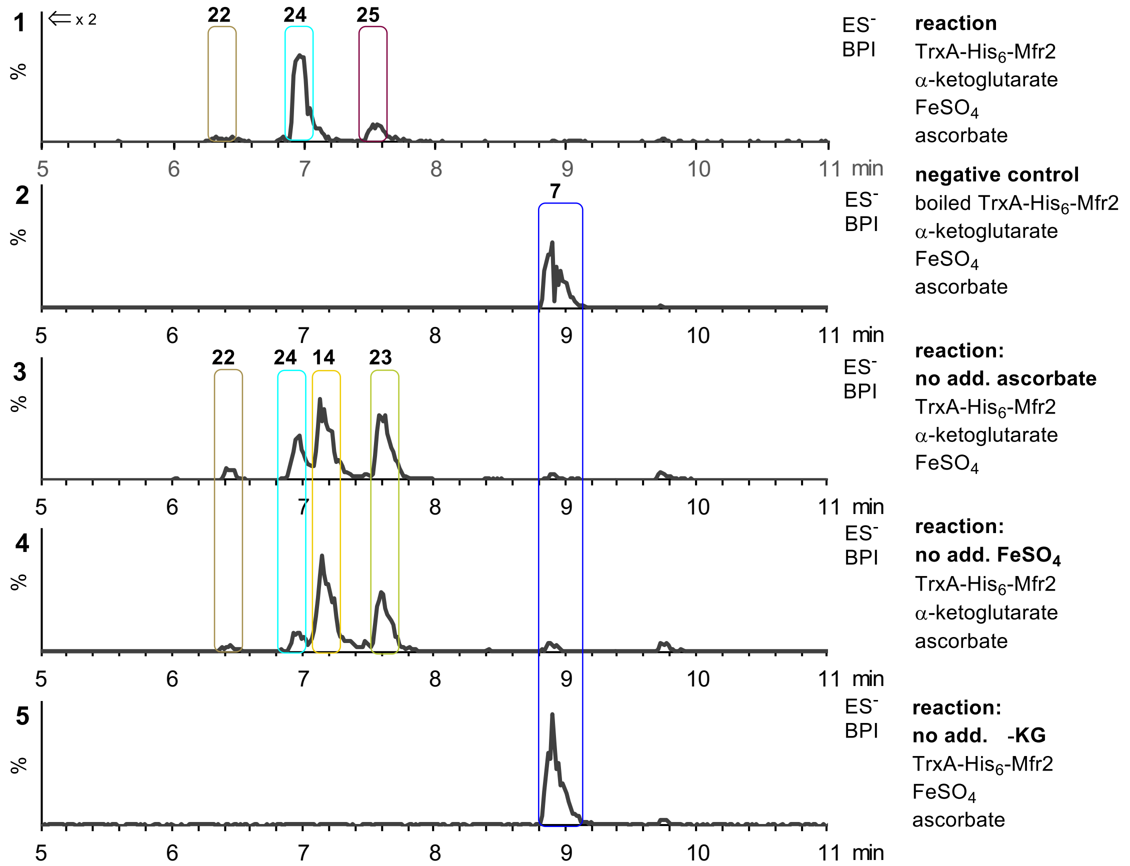

Figure S49 LCMS analysis of *in vitro* reactions with TrxA-His_6-Mfr2 and boiled control (arbitrary units). See Fig. S75 for predicted structures.

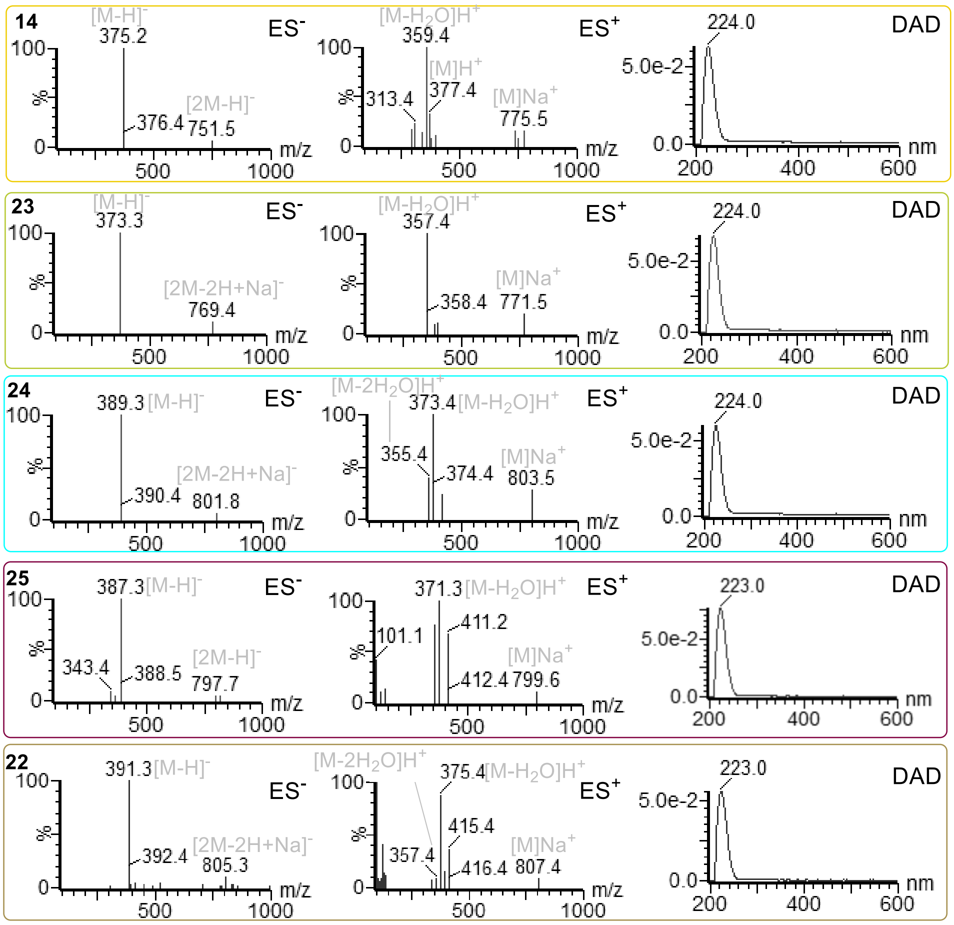

Figure S50 MS spectra (ES$^-$, ES$^+$, DAD) of products generated by Mfr2 *in vitro*. See Fig. S75 for predicted structures.

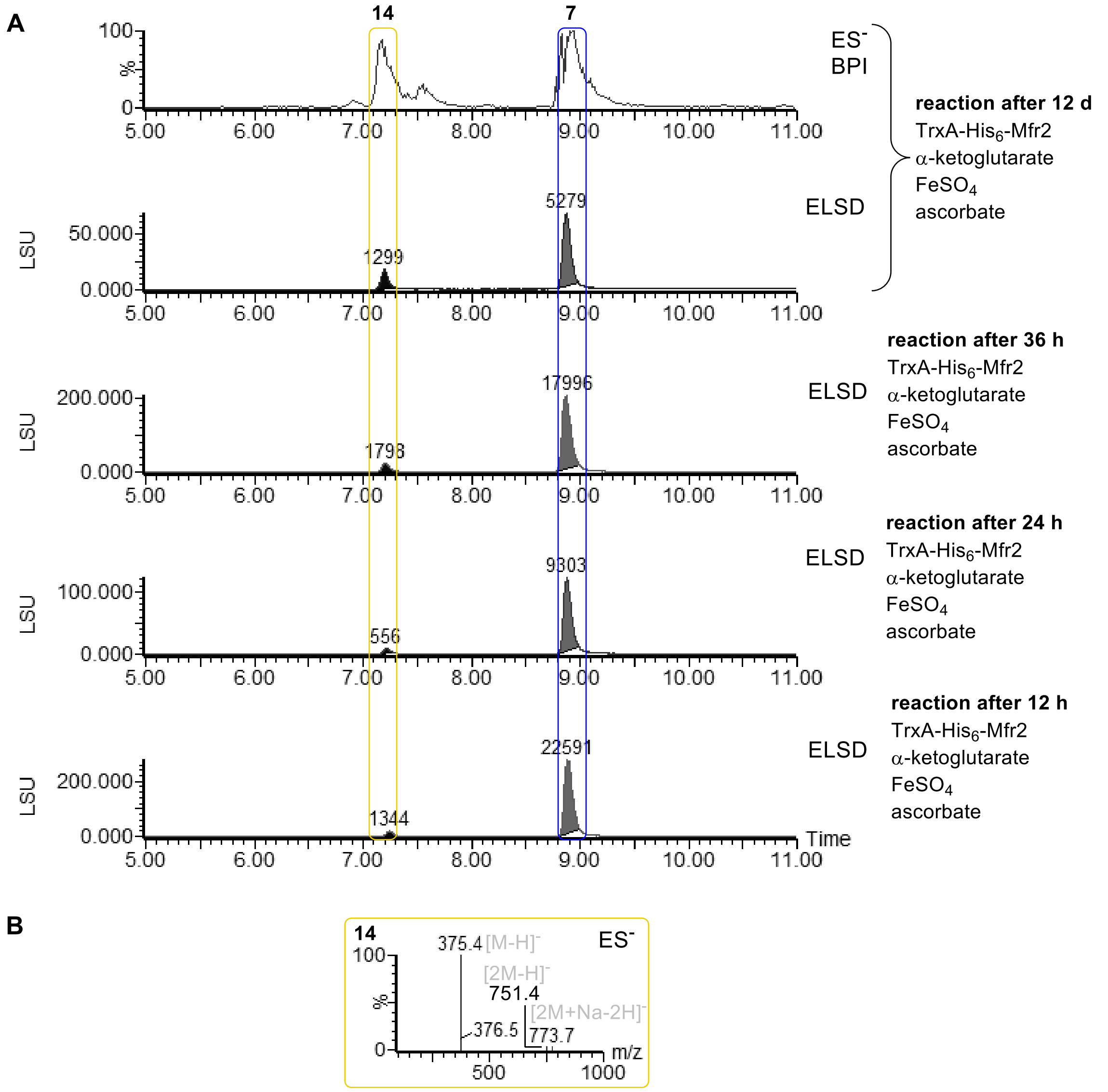

Figure S51 LCMS analysis of large-scale TrxA-His$_6$-Mfr2 *in vitro* assay. **A**, LCMS chromatograms; **B**, MS spectrum of product.

9.1 Characterization of 7-Hydroxy-CJ-13,982, 14

Table S24 Chemical shifts of **14** in CD_3OD (600 MHz).

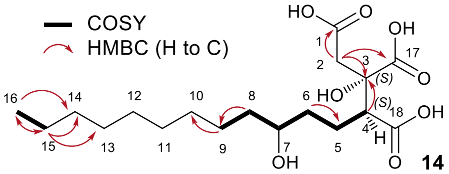

Chemical Formula: $C_{18}H_{32}O_8$
Mass: 376.21

Pos.	δ$_H$/ ppm (mult, *J* in Hz)	δ$_C$/ ppm	C-type	HMBC H to C	COSY
**1**	-	174.8	CO	-	-
**2a**	3.0 (d, 16.5)	43.7	CH_2	1	2b
**2b**	2.67 (d, 16.5)	43.7	CH_2	1, 3, 17	2a
**3**	-	77.1	C	-	-
**4**	2.70 (m)	54.4	CH	3	5a, 5b
**5a**	1.92 (m)	24.6	CH_2	-	4, 5b, 6
**5b**	1.50 (m)	24.6	CH_2	-	4, 5a, 6
**6**	1.42 (m)	36.5	CH_2	5	5a, 5b, 7
**7**	3.52 (bs)	72.0	CH	-	6, 8
**8**	1.41 (m)	38.3	CH_2	9	7
**9**	1.39 (m)	26.8	CH_2	10-13	10-14
**10†**	1.29 (m)	30.9	CH_2	9, 11-13	9, 11-13
**11†**	1.29 (m)	30.8	CH_2	10-13	10-13
**12†**	1.29 (m)	30.8	CH_2	10-13	10-13
**13†**	1.29 (m)	30.5	CH_2	10-13	10-13
**14**	1.29 (m)	33.1	CH_2	10-13	10-13
**15**	1.29 (m)	23.8	CH_2	13, 14, 16	16
**16**	0.90 (t, 6.9)	14.4	CH_3	15, 14	15
**17**	-	178.0	CO	-	-
**18**	-	177.3	CO	-	-

† These signals are indistinguishable from each other.

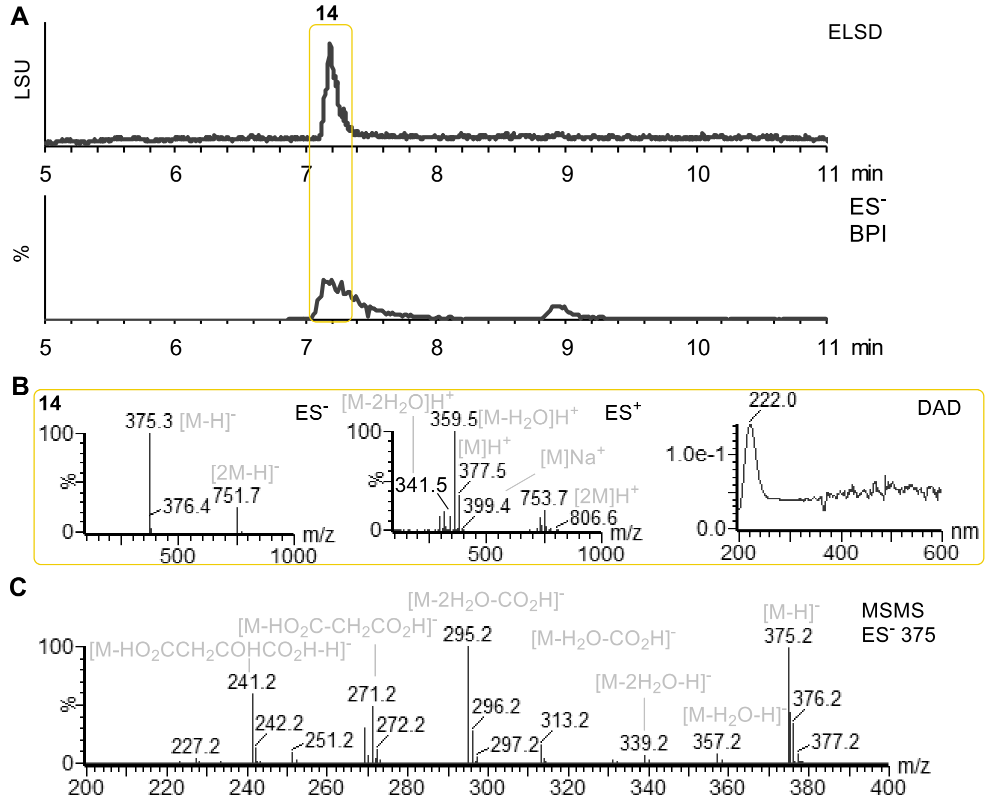

D

Elemental Composition Report

Single Mass Analysis
Tolerance = 20.0 PPM / DBE: min = -1.5, max = 50.0
Element prediction : Off
Number of isotopes peaks used for i-FIT = 3

Monoisotopic Mass, Even Electron Ions
76 formula(e) evaluated with 8 results within limits (all results (up to 1000) for each mass)
Elements Used:
C: 0-40 H: 0-50 O: 0-8 Na: 0-1

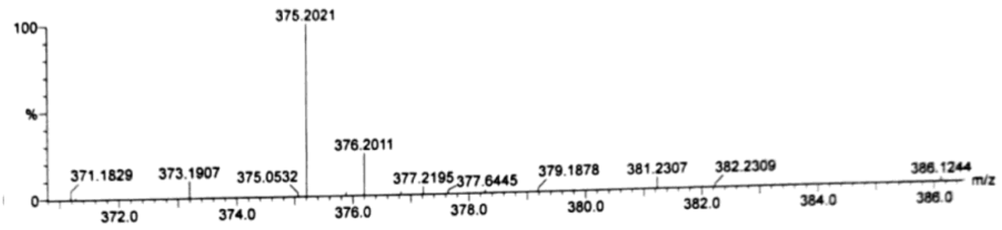

Minimum:				-1.5			
Maximum:		5.0	50.0	50.0			
Mass	Calc. Mass	mDa	PPM	DBE	i-FIT	i-FIT (Norm)	Formula
375.2021	375.2019	0.2	0.5	3.5	12.8	1.5	C18 H31 O8
	375.1995	2.6	6.9	0.5	13.2	1.8	C16 H32 O8 Na
	375.1960	6.1	16.3	12.5	13.0	1.7	C25 H27 O3
	375.2089	-6.8	-18.1	13.5	13.4	2.1	C27 H28 Na
	375.1936	8.5	22.7	9.5	13.2	1.9	C23 H28 O3 Na
	375.2113	-9.2	-24.5	16.5	14.1	2.8	C29 H27
	375.2147	-12.6	-33.6	4.5	14.1	2.8	C20 H32 O5 Na
	375.2171	-15.0	-40.0	7.5	14.5	3.2	C22 H31 O5

Figure S52 LCMS characterization and structure elucidation of **14**. **A**, ELSD and ES^- traces of purified material (arbitrary units); **B**, DAD, ES^- and ES^+ spectra of **14**; **C**, MSMS analysis of **14**; **D**, HRMS analysis.

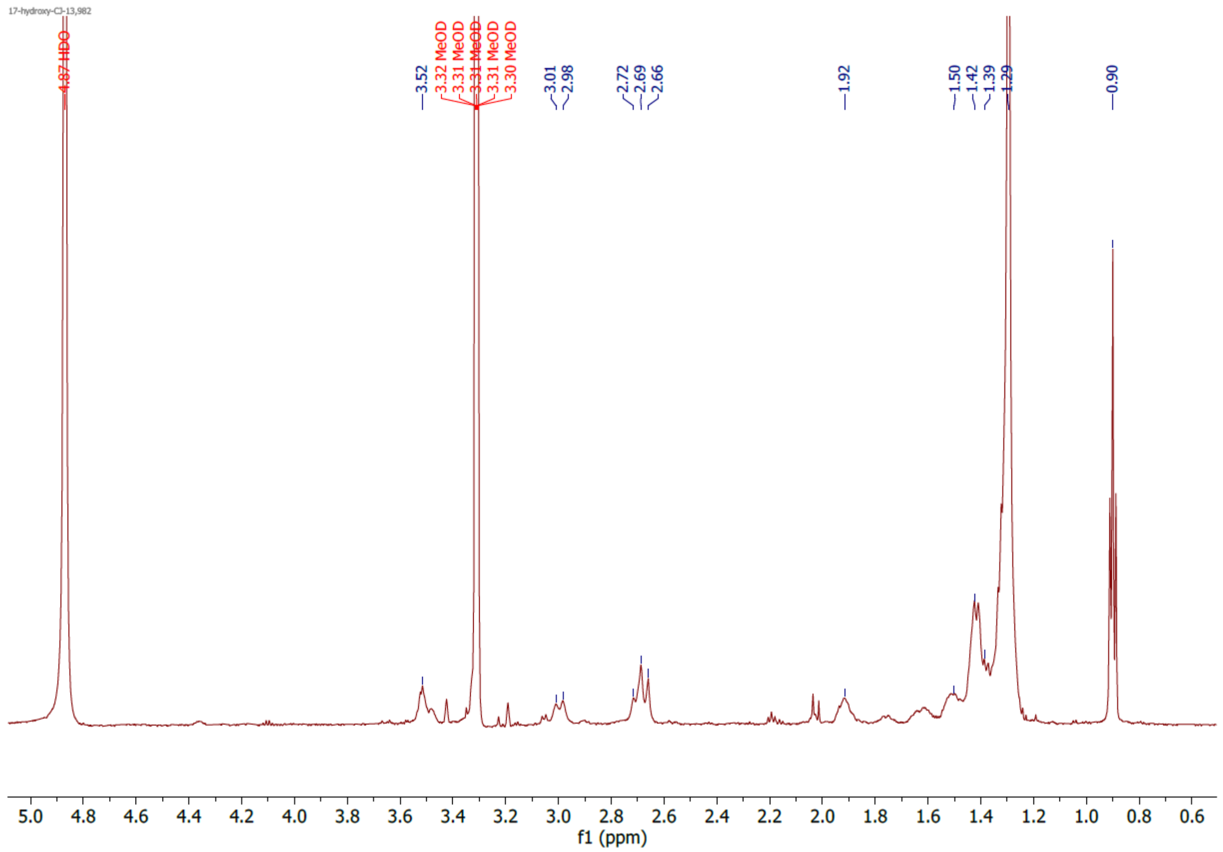

Figure S53 ^{1}H NMR of **14** in CD_3OD (600 MHz). See Table S24 for assignment.

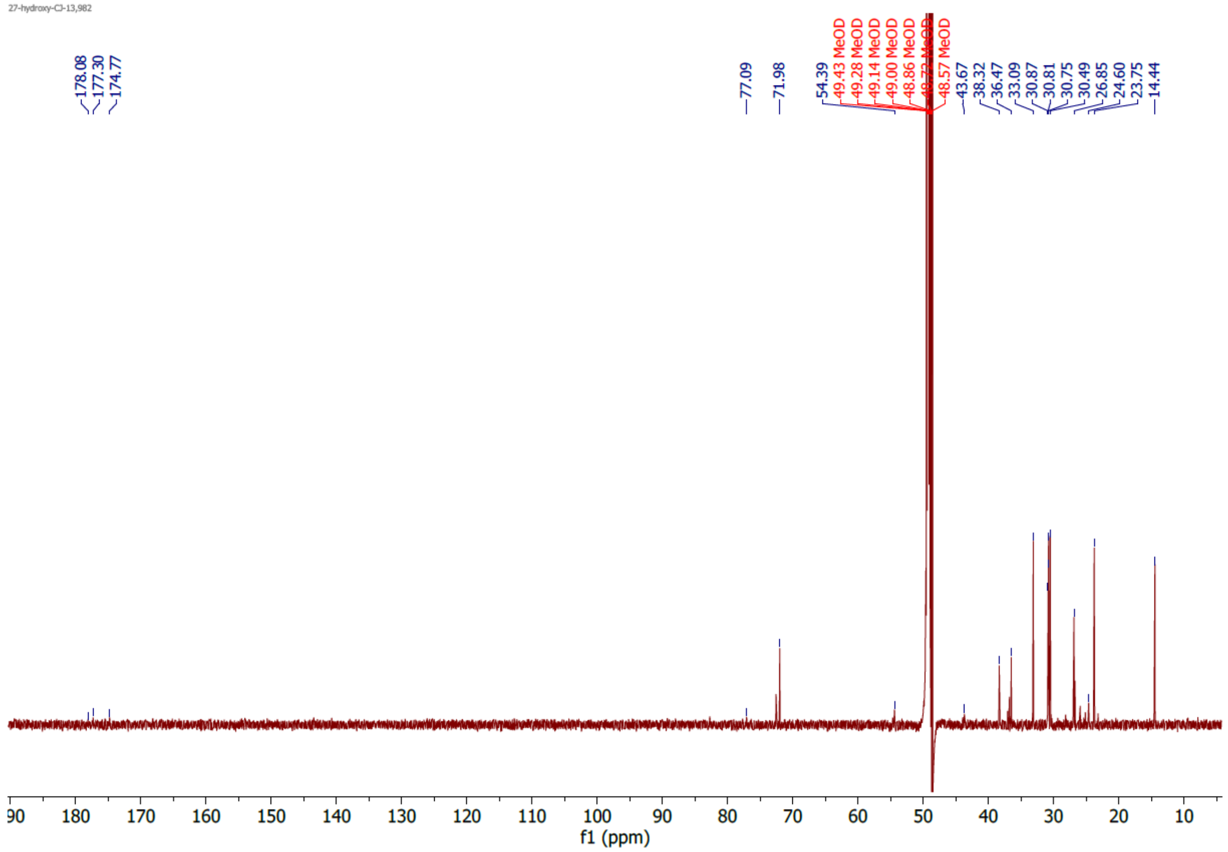

Figure S54 ^{13}C NMR of **14** in CD_3OD (150 MHz). Table S24 for assignment.

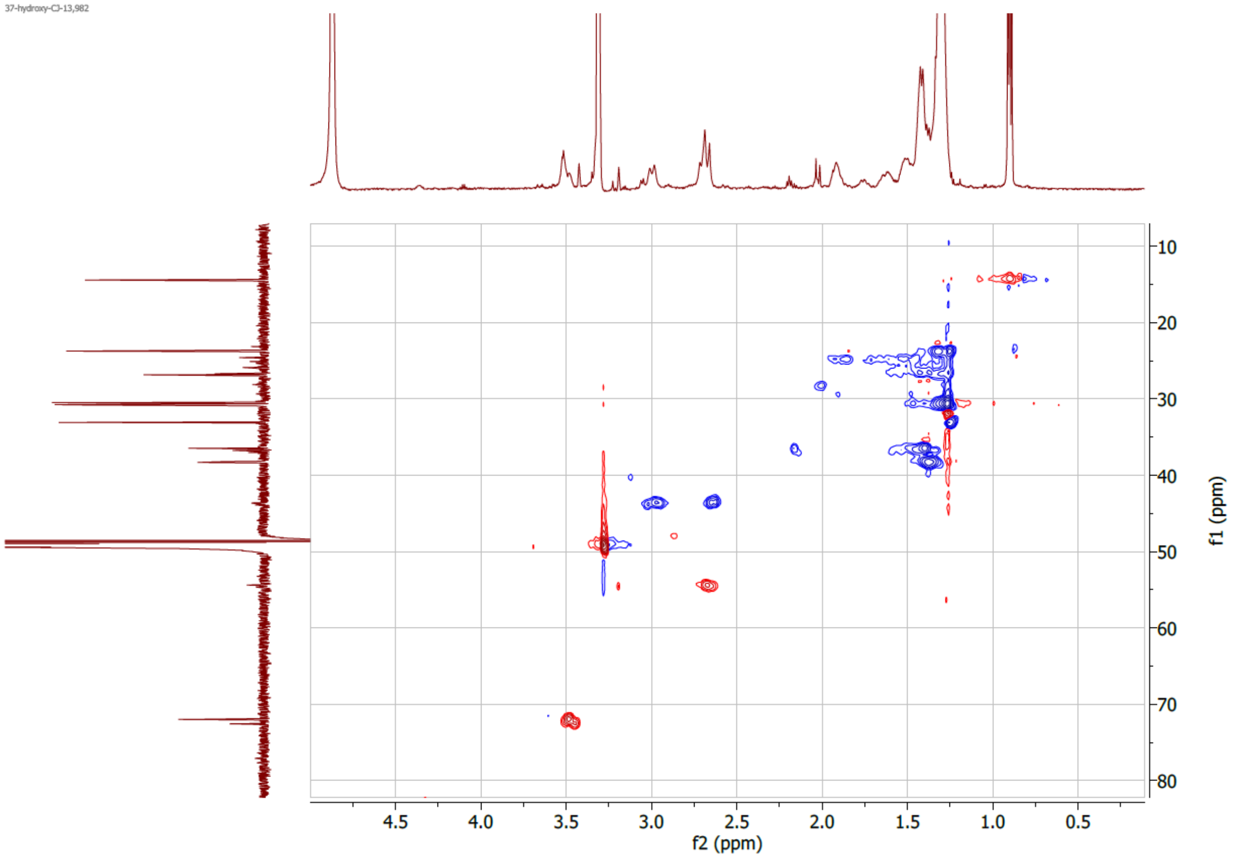

Figure S55 HSQC NMR of **14** in CD_3OD.

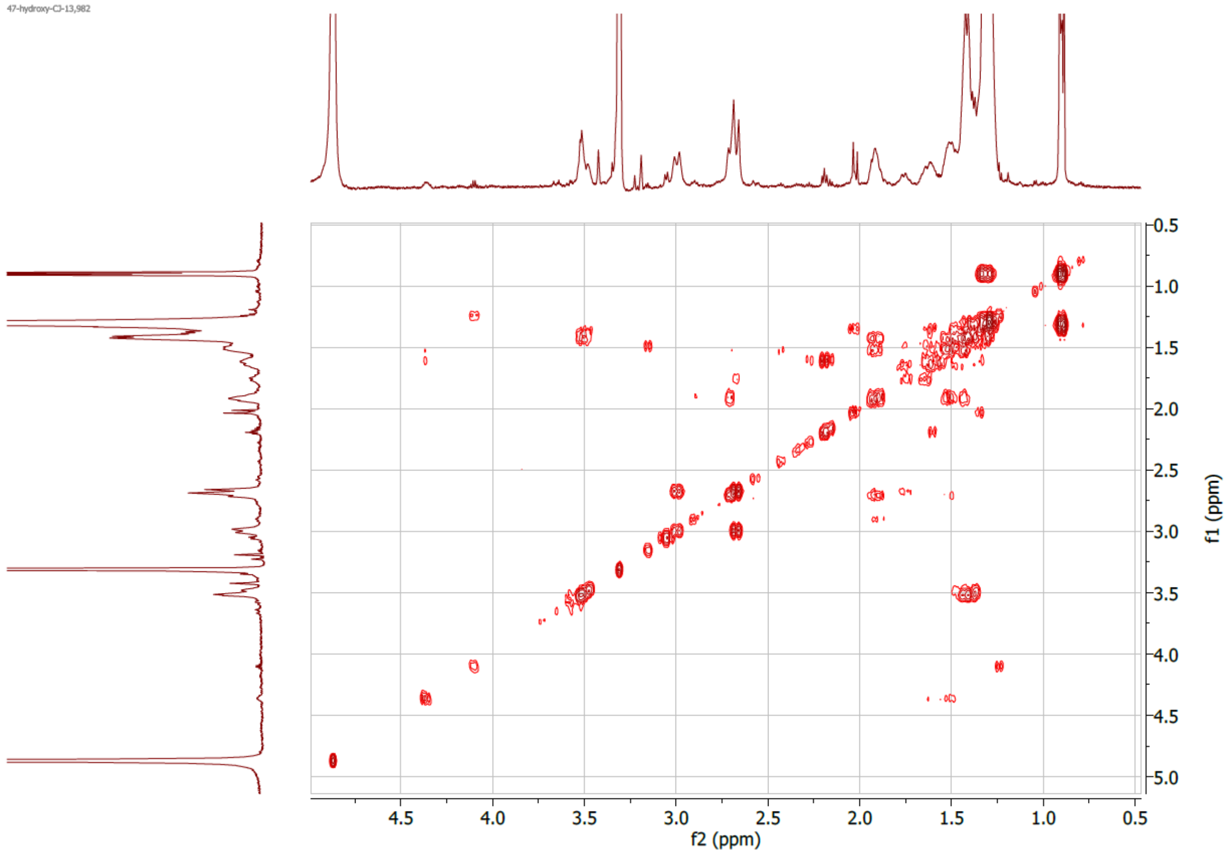

Figure S56 ^{1}H, ^{1}H COSY of **14** in CD_3OD.

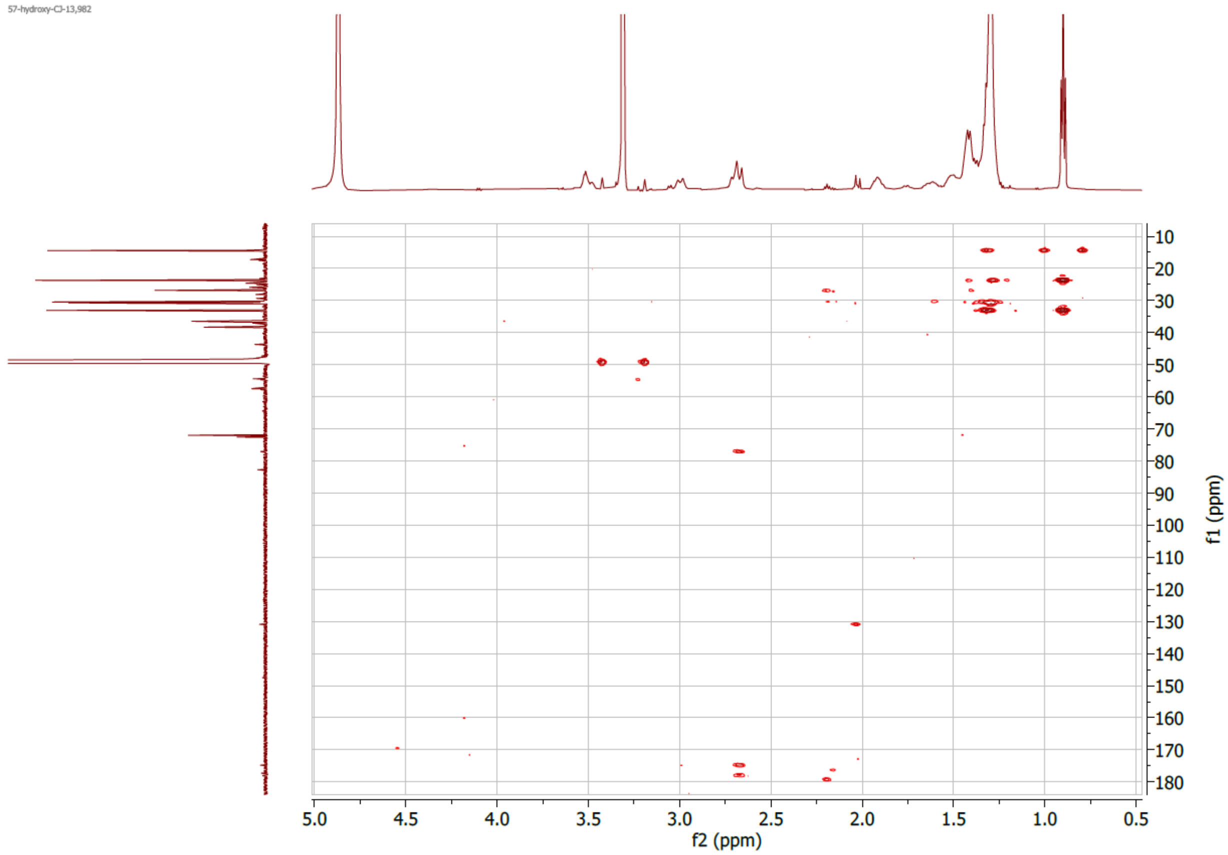

Figure S57 HMBC NMR of **14** in CD_3OD.

10. EXPERIMENT 6 (7 + MfR1 + Mfr2) *in vitro*

In reactions that contained both, TrxA-His$_6$-Mfr1 and TrxA-His$_6$-MfR2 (Fig. S58A2) the three compounds eluting at t_R = 7.5 min with *m/z* ES$^-$ = 387 Da (**25**, Fig. S58B); t_R = 6.9 min with *m/z* ES- = 389 Da (**24**, Fig. S58B); t_R = 6.5 min with *m/z* ES$^-$ = 391 Da (**22**, Fig. S58B) were detected. Additionally another compound eluted at t_R = 7.1 min with *m/z* ES$^-$ = 403 Da (corresponds to 5,6-epoxy-4-hydroxy-7-oxo-CJ-13,982 **26**, Fig. S58B)) that was not detected in reactions containing only one of the oxygenases. A barely detectable peak with *m/z* 419.2 Da (Fig. S58A1, Fig. S58B) at 6.8 min may correspond to either **27** or **28**. Attempts to purify the produced compounds for characterization were not successful.

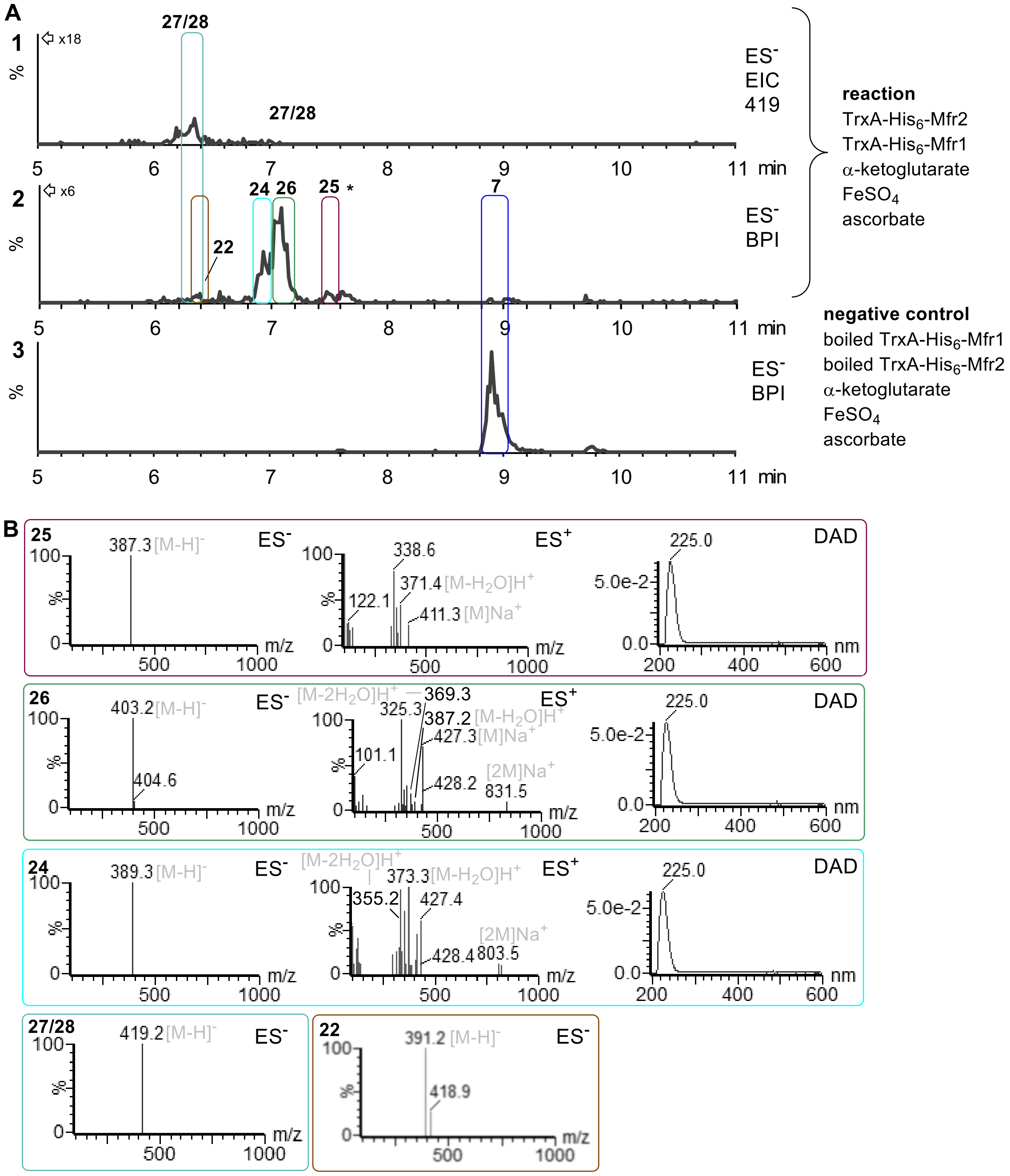

Figure S58 *In vitro* studies with recombinant TrxA-His$_6$-Mfr2 and TrxA-His$_6$-Mfr1. **A**, LCMS analysis of *in vitro* assays with recombinant TrxA-His$_6$-Mfr2 and TrxA-His$_6$-Mfr1 and boiled TrxA-His$_6$-Mfr2 and TrxA-His$_6$-Mfr1 as control (arbitrary units, zoom is indicated by (←x) where applied, ELSD trace smoothed in Excel); **B**, ES$^-$ (and ES$^+$, DAD) spectra of products. See Fig. S75 for predicted structures.

11. EXPERIMENT 7 (*ctrR4* + *ctrR3* + *ctrACS* + *mfr1*) *in vivo*

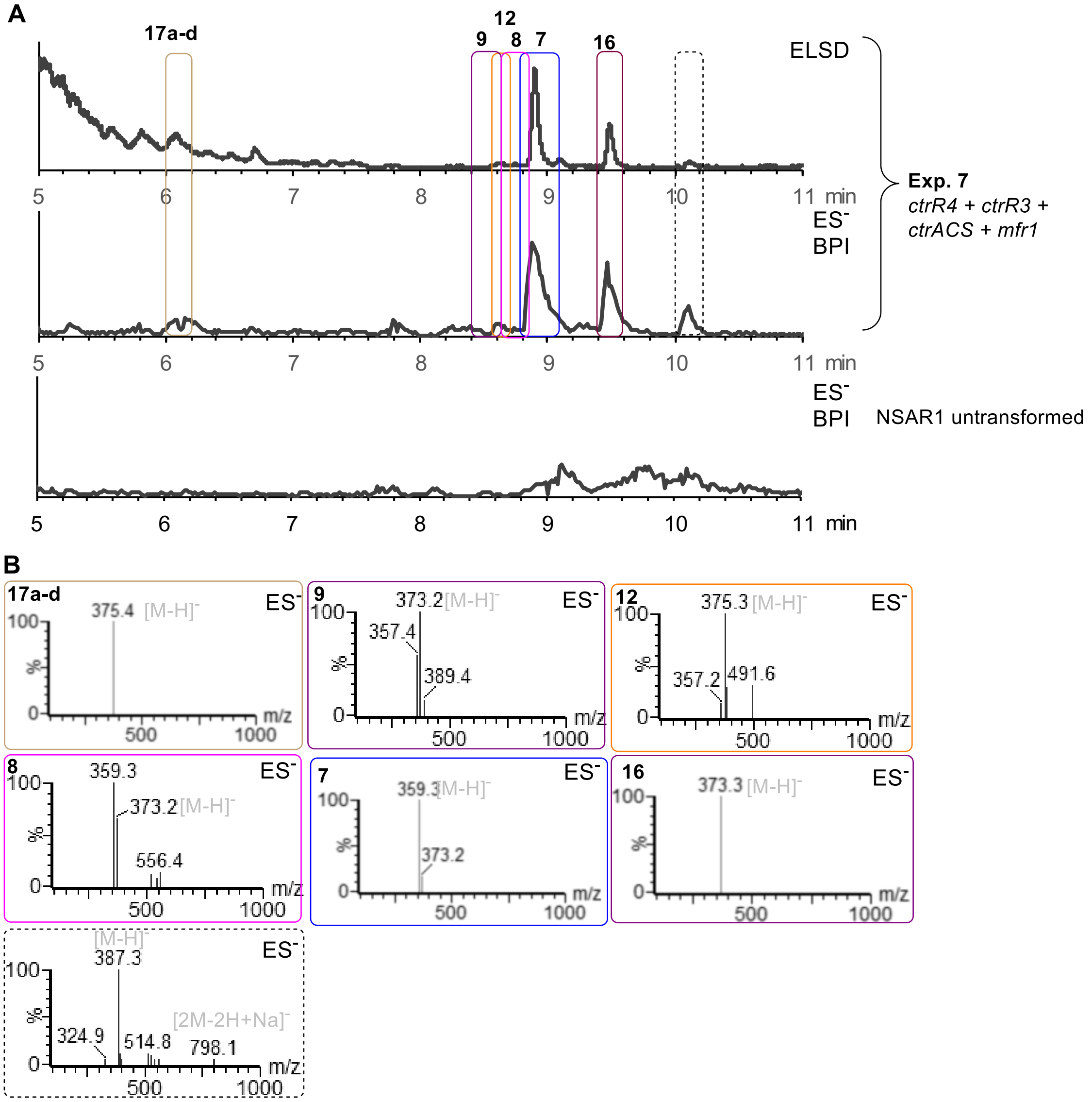

Figure S59 Heterologous expression of the structural genes of the *ctr* BGC *ctrR4, ctrR3* and *ctrACS,* and *SS1* BGC *mfr1*. **A**, ES⁻ (and ELSD) chromatograms of extracts of *A. oryzae* NSAR1 transformants carrying genes from the *SS1* BGC and *ctr* BGC and untransformed control (arbitrary units); **B**, ES⁻ spectra of compounds detected in an *A. oryzae* NSAR1 transformants.

12. EXPERIMENT 8 (*ctrR4* + *ctrR3* + *ctrACS* + *mfr2*) *in vivo*

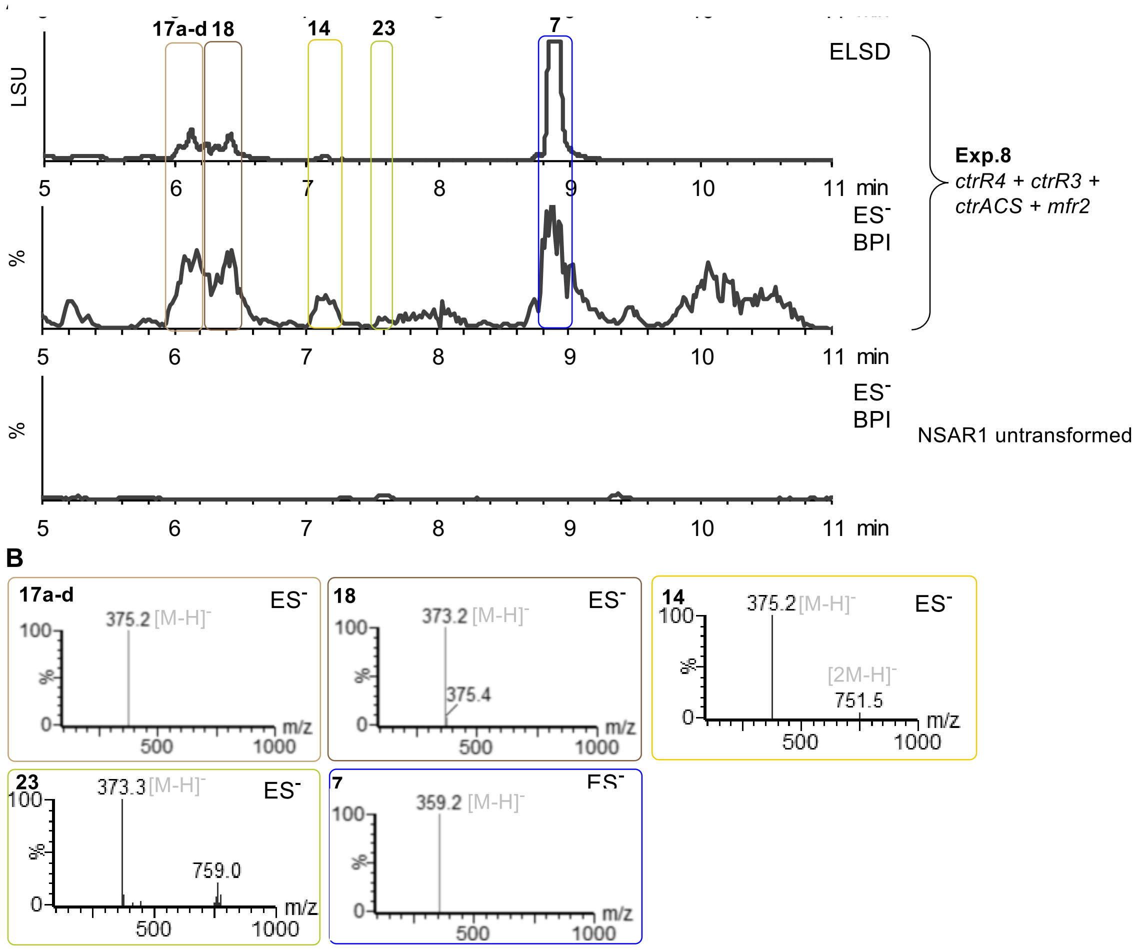

Figure S60 A, Heterologous expression of the structural genes of the *ctr* BGC *ctrR4, ctrR3* and *ctrACS,* and *SS1* BGC *mfr2*. **A**, ES$^-$ (and ELSD) chromatograms of extracts of *A. oryzae* NSAR1 transformants carrying genes from the *SS1* BGC and *ctr* BGC and untransformed control (arbitrary units); **B**, ES$^-$ spectra of compounds detected in an *A. oryzae* NSAR1 transformants. See Fig. S75 for predicted structures.

13. EXPERIMENT 9 (*ctrR4* + *ctrR3* + *ctrACS* + *mfr1* + *mfr2*) *in vivo*

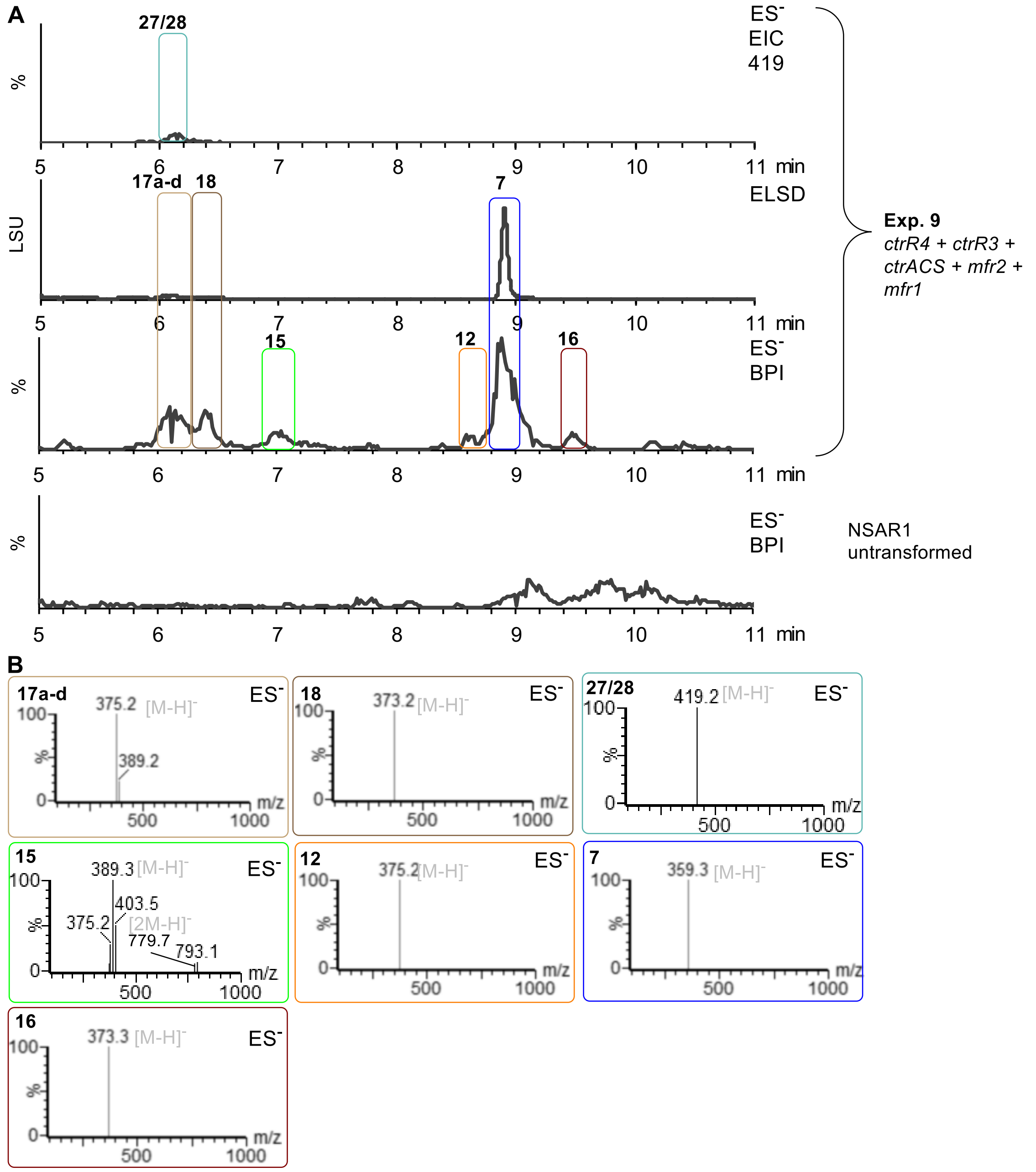

Figure S61 LCMS analysis of extracts of a representative transformant of the *ctr* BGC *ctrR4*, *ctrR3* and *ctrACS*, and *SS1* BGC *mfr2* and *mfr1*. **A**, ES⁻, ELSD and EIC chromatograms of extracts of *A. oryzae* NSAR1 transformants carrying indicated genes and untransformed control (arbitrary units); **B**, ES⁻ spectra of compounds detected in extracts of *A. oryzae* NSAR1 transformant from *A. oryzae* + *ctrR4* + *ctrR3* + *ctrACS* + *mfR2* + *mfR1*. See Fig. S75 for proposed structures.

14. EXPERIMENT 10 (*ctrR4* + *ctrR3* + *ctrACS* + *ctrR1* + *mfr2*) *in vivo*

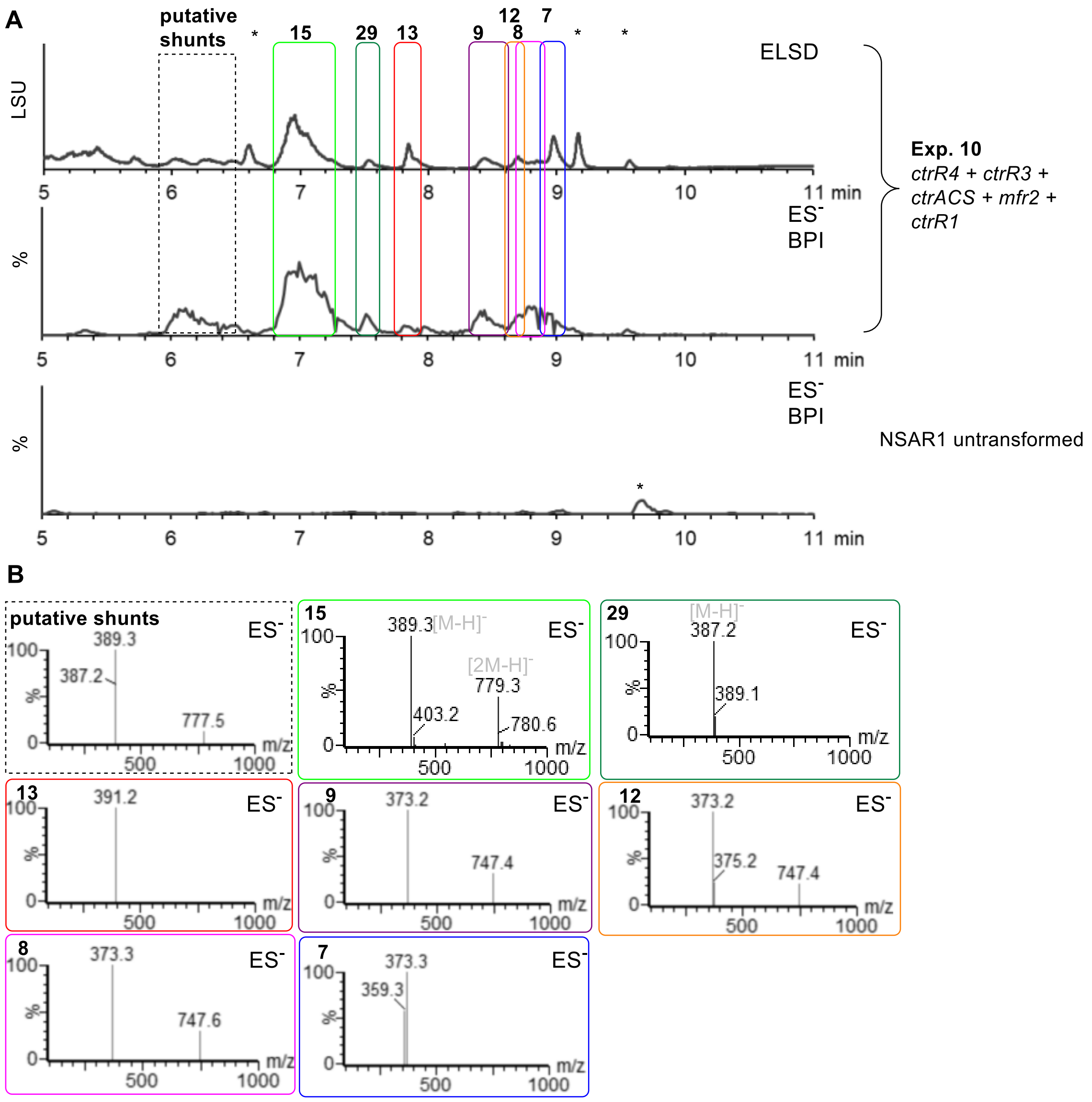

Figure S62 LCMS analysis of extracts of a representative transformant of indicated experiments. **A**, ES⁻ and ELSD chromatograms of extracts of *A. oryzae* NSAR1 transformants carrying indicated genes and untransformed control (arbitrary units, * = unrelated compounds); **B**, ES⁻, ES⁺ and DAD spectra of new compounds detected in extracts of *A. oryzae* NSAR1 transformant from indicated peaks. See Fig. S75 for predicted structures.

14.1 Characterization of 7-Hydroxy-Cinatrin C_3, 15

Table S25 Chemical shifts of **15** in CD_3OD (600 MHz).

Chemical Formula: $C_{18}H_{30}O_9$
Mass: 390.19

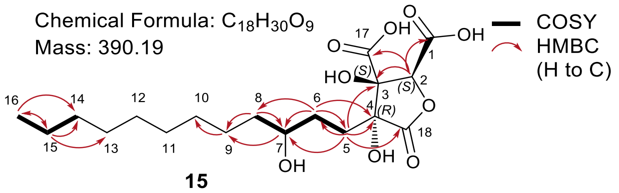

15

7-hydroxy-cinatrin C3

Pos.	δ_H/ ppm (mult, *J* in Hz)	δ_C/ ppm	C-type	HMBC H to C	COSY
**1**	-	169.8	CO	-	-
**2**	5.38 (s)	82.2	CH	1, 3, 17	-
**3**	-	81.7	C	-	-
**4**	-	80.1	C	-	-
**5a**	2.06 (m)	28.2	CH_2	3, 4, 6, 7, 18	5b, 6
**5b**	1.83 (ddd, 14, 9.8, 6.5)	28.2	CH_2	4, 6, 7, 18	5a, 6
**6**	1.64 (ddt, 14, 9.8, 7.5)	30.1	CH_2	4, 5, 7, 8	5a, 5b, 7
**7**	3.53 (m)	72.8	CH	9	6, 8
**8**	1.45 (m)	38.3	CH_2	7, 9	7
**9**	1.45 (m)	26.8	CH_2	10	10-15
**10**	1.32 (m)	30.7	CH_2	11-15	11-15
**11†**	1.32 (m)	30.8	CH_2	10-15	10-15
**12†**	1.32 (m)	30.9	CH_2	10-15	10-15
**13†**	1.32 (m)	30.5	CH_2	10-15	10-15
**14**	1.32 (m)	33.1	CH_2	10-15	10-15
**15**	1.32 (m)	23.7	CH_2	13, 14, 16	16
**16**	0.92 (t, 7)	14.4	CH_3	14, 15	15
**17**	-	173.6	CO	-	-
**18**	-	176.4	CO	-	-

† These signals are indistinguishable from each other

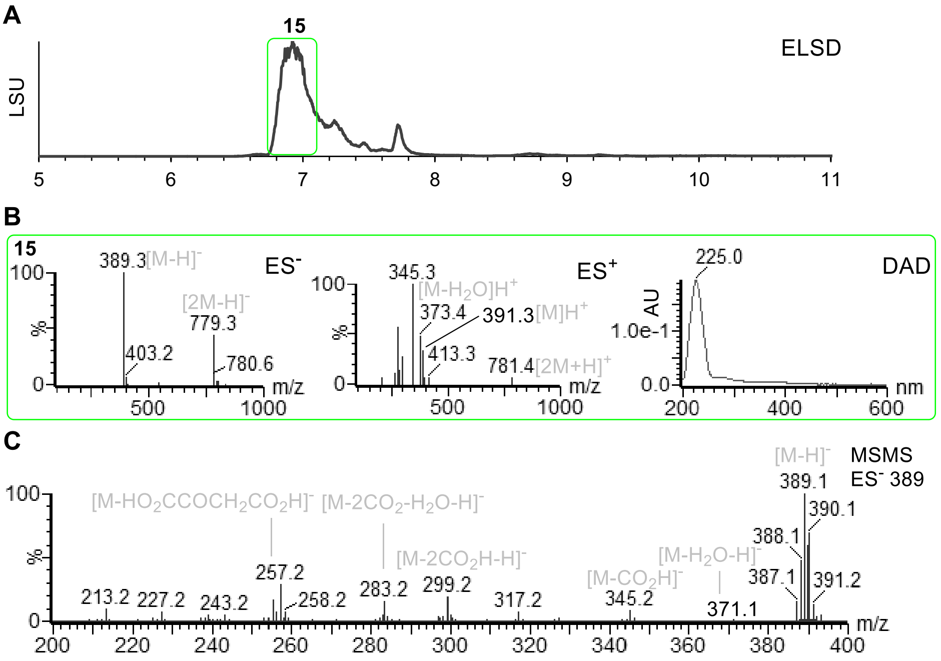

D

Elemental Composition Report

Single Mass Analysis
Tolerance = 20.0 PPM / DBE: min = -1.5, max = 50.0
Element prediction : Off
Number of isotopes peaks used for i-FIT = 3

Monoisotopic Mass, Even Electron Ions
1056 formula(e) evaluated with 34 results within limits (all results (up to 1000) for each mass)
Elements Used:
C: 0-90 H: 0-135 N: 0-20 O: 0-17

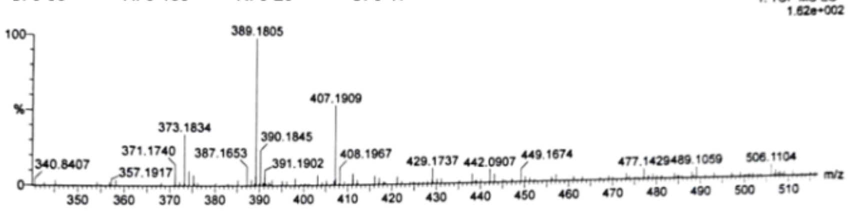

Mass	Calc. Mass	mDa	PPM	DBE	i-FIT	i-FIT (Norm)	Formula
Minimum:				-1.5			
Maximum:		5.0	20.0	50.0			
389.1805	389.1812	-0.7	-1.8	4.5	27.3	2.1	C18 H29 O9
	389.1812	-0.7	-1.8	10.0	27.7	2.5	C17 H23 N7 O4
	389.1798	0.7	1.8	5.0	27.8	2.5	C16 H27 N3 O8
	389.1798	0.7	1.8	10.5	28.2	3.0	C15 H21 N10 O3
	389.1817	-1.2	-3.1	3.0	31.1	5.9	C2 H19 N19 O5
	389.1785	2.0	5.1	5.5	28.5	3.3	C14 H25 N6 O7
	389.1785	2.0	5.1	11.0	29.0	3.8	C13 H19 N13 O2

Figure S63 LCMS characterization of **15**. **A**, ELSD chromatogram of **15**; **B**, ES^-, ES^+ and DAD spectra of **15**; **C**, MSMS data obtained for **15**; **D**, HRMS analysis and calculated molecular formula of **15**.

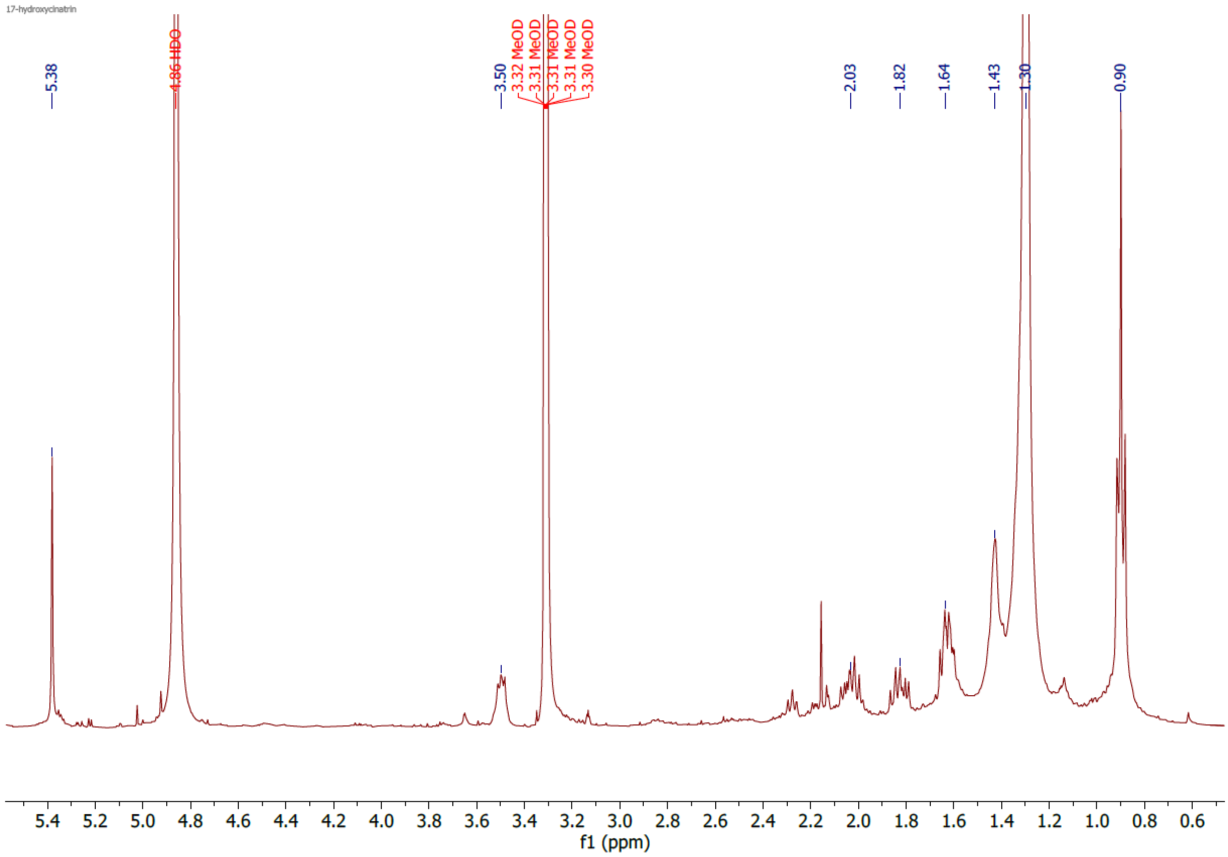

Figure S64 ^{1}H NMR of **15** in CD_3OD (600 MHz). See Table S25 for assignment.

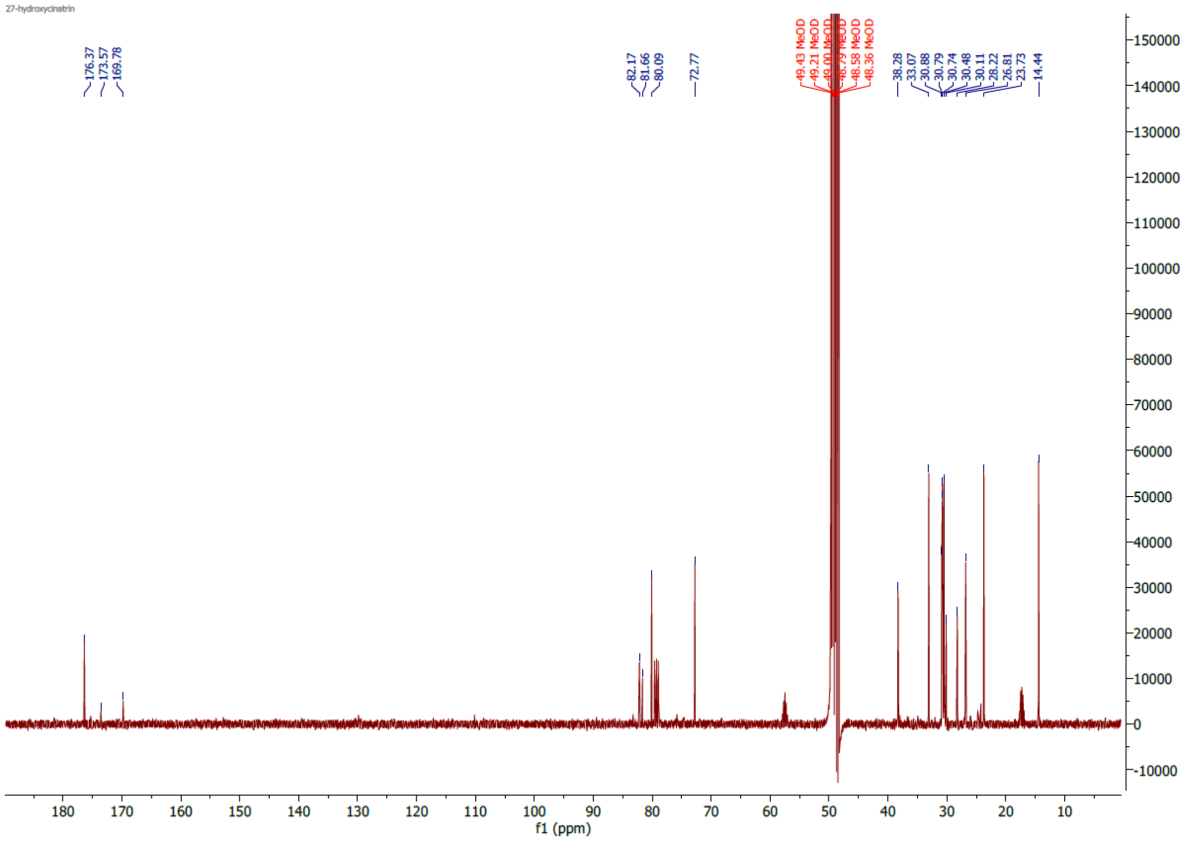

Figure S65 ^{13}C NMR of **15** in CD_3OD (150 MHz). See Table S25 for assignment.

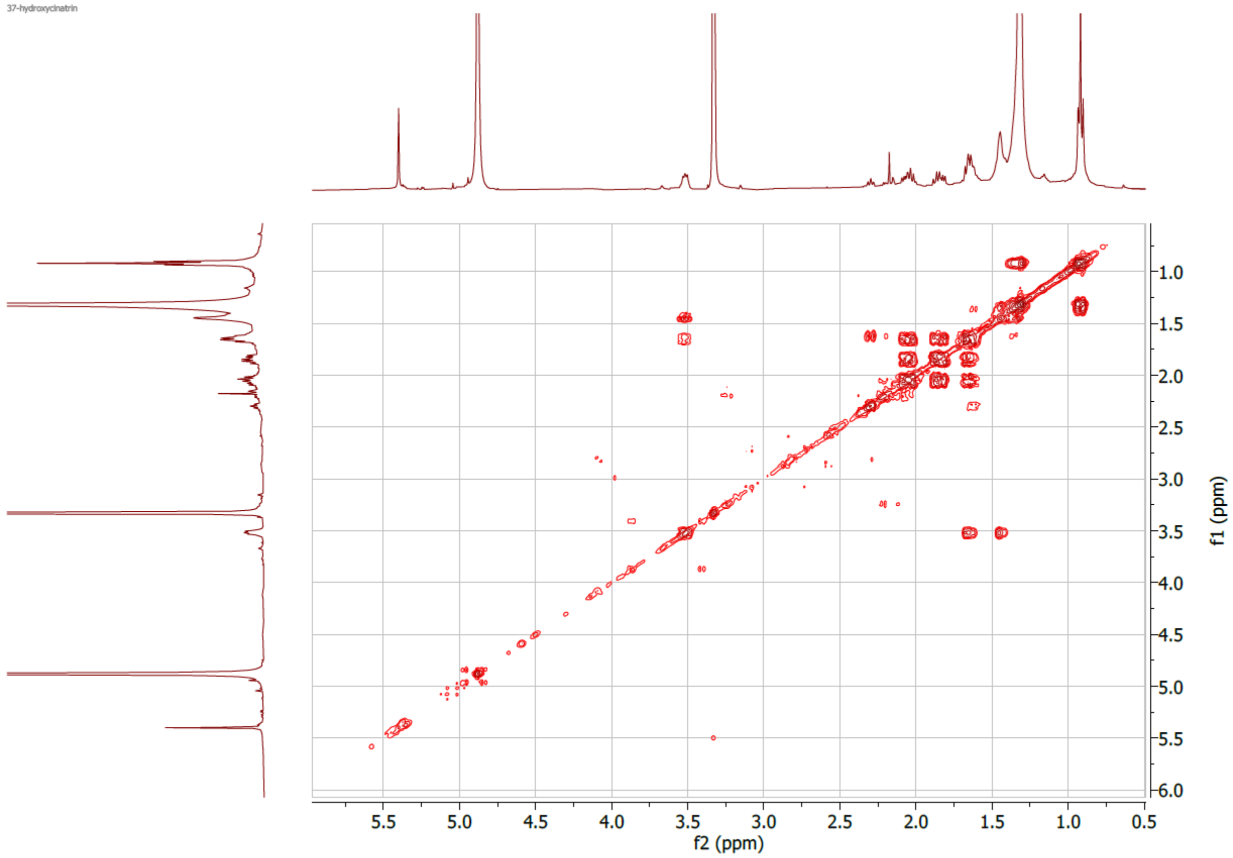

Figure S66 ^{1}H, ^{1}H COSY NMR spectrum of **15** in CD_3OD.

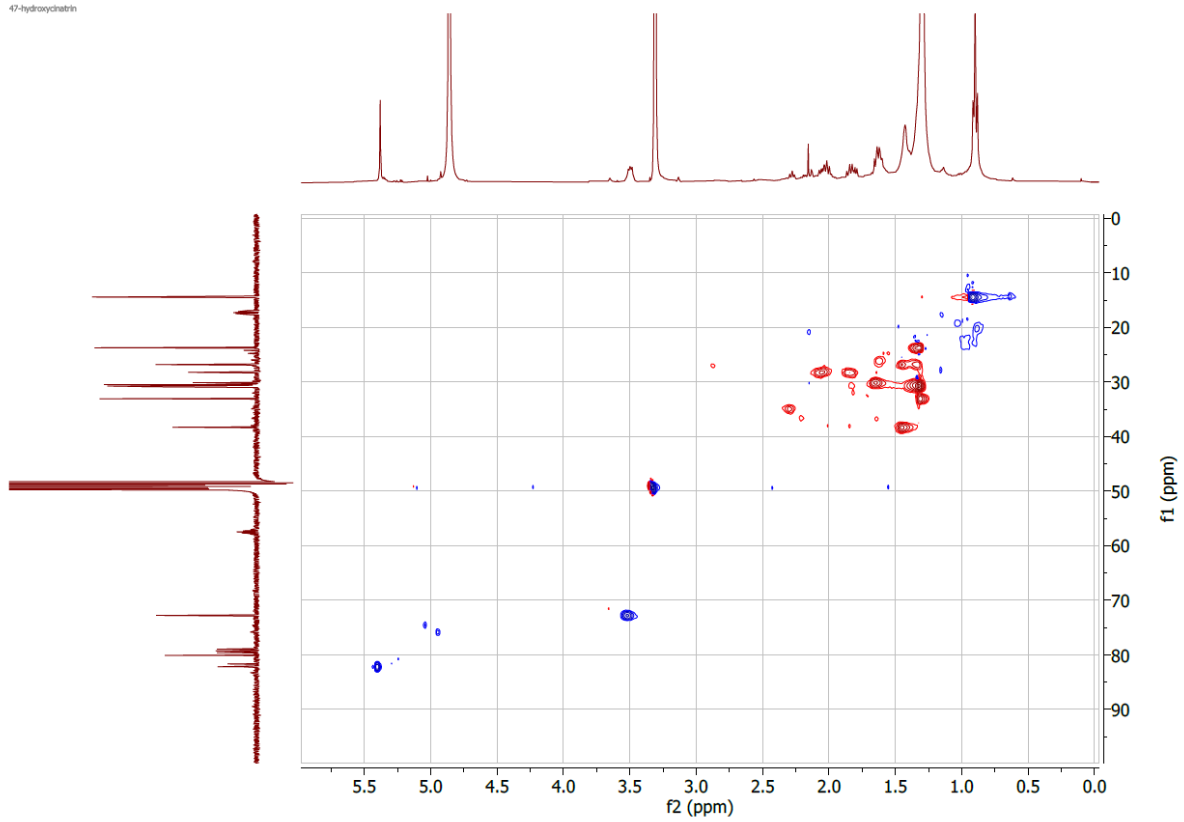

Figure S67 HSQC NMR spectrum of **15** in CD_3OD.

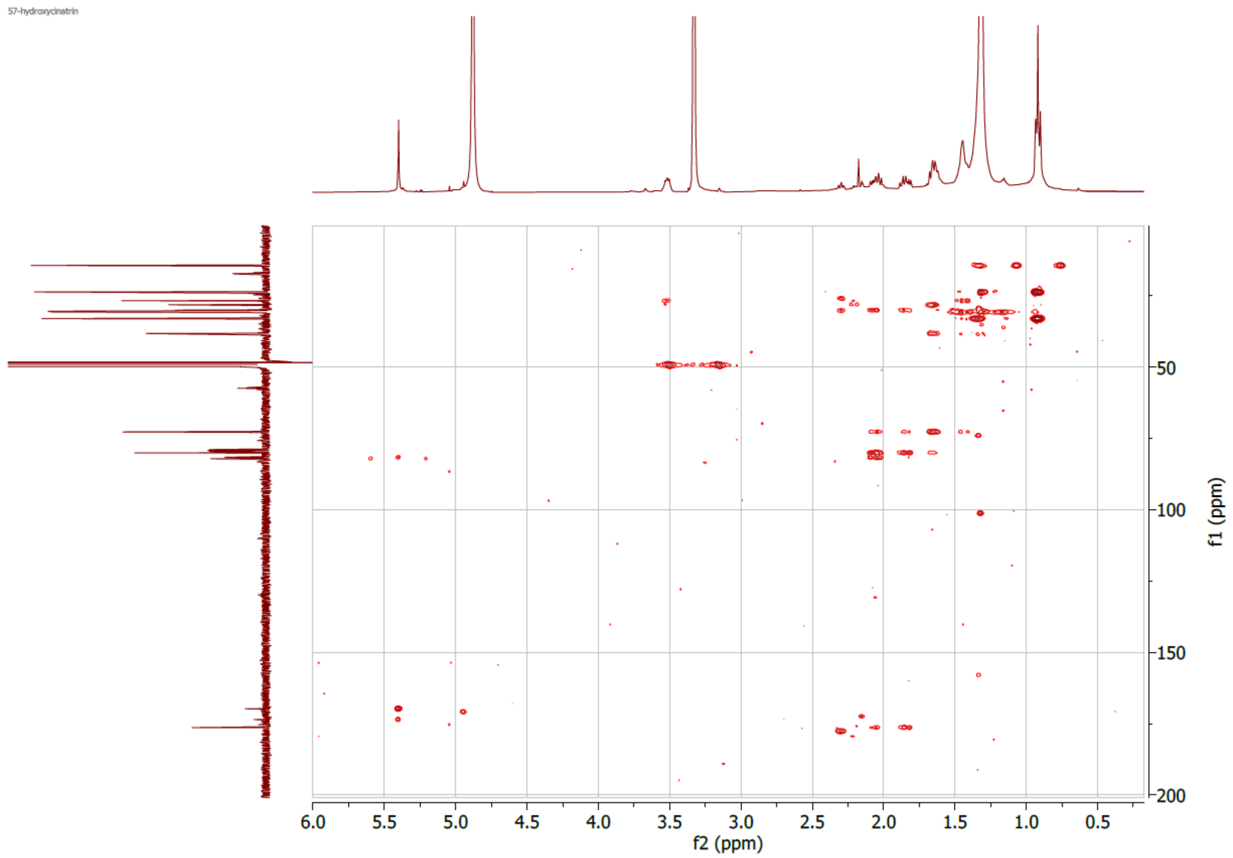

Figure S68 HMBC NMR of **15** in CD_3OD.

15. Chemical Synthesis

15.1 Tris(tetrabutylammonium)hydrogenphosphate 19

Tris(tetrabutylammonium)hydrogenphosphate **19** was synthesized according to Oberhauser et al.[22] Disodium-dihydrogenpyrophosphate (3.13 g, 14 mmol) was dissolved in 25 mL deionized H_2O containing 1 mL concentrated ammonia (25 % in H_2O) and eluted with water through a column containing ion exchange resin (Amberchrom® 50WX8, H^+-form. 100-200 mesh). The solution was then titrated with tetra-n-butylammoniumhydroxide to pH 7.3. Lyophilization afforded the compound as a flocculant white solid material (12.25 g, 13.6 mmol, 97 %).

^{1}H-NMR (D_2O, 400 MHz): δ = 3.16-3.22 (m, 24H), 1.50-1.62 (m, 24H), 1.27 (sex, *J* =7.4 Hz, 24H), 0.86 (t, *J* = 7.4 Hz, 36H) ppm. ^{31}P-NMR (D_2O, 162 MHz): δ = -7.35 (s, 2P) ppm.

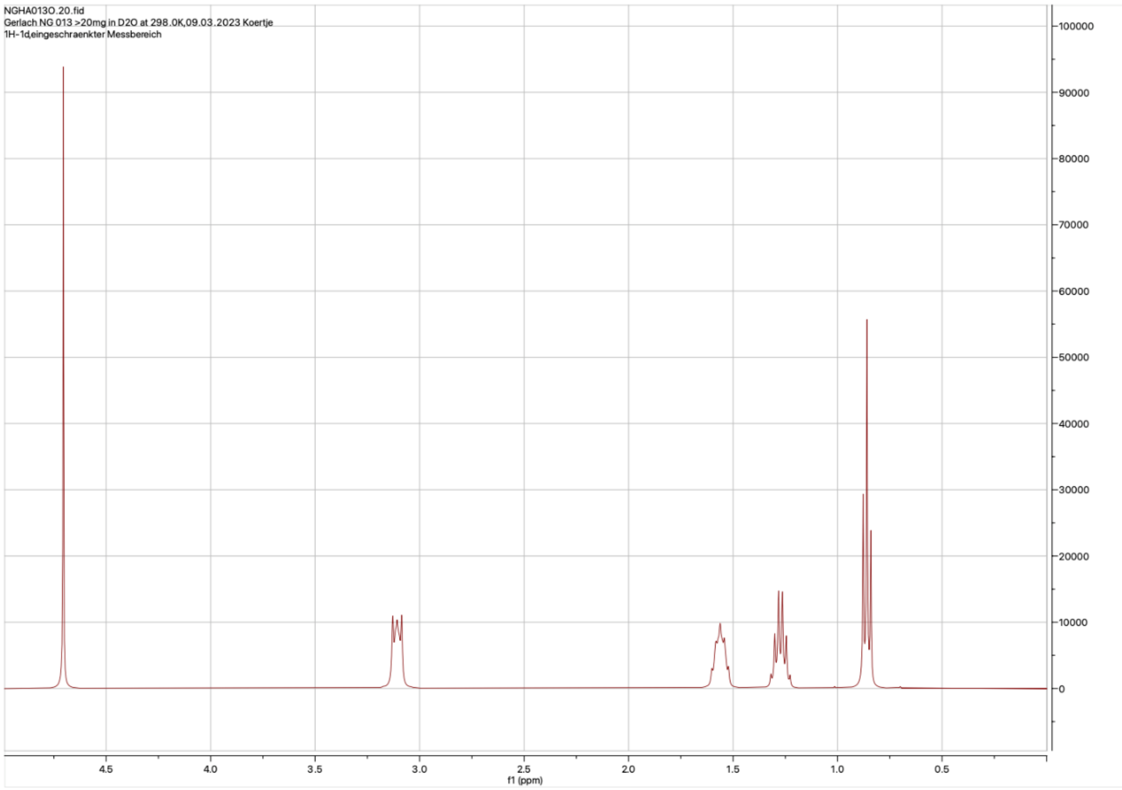

Figure S69 ^{1}H NMR of **19** in D_2O (400 MHz).

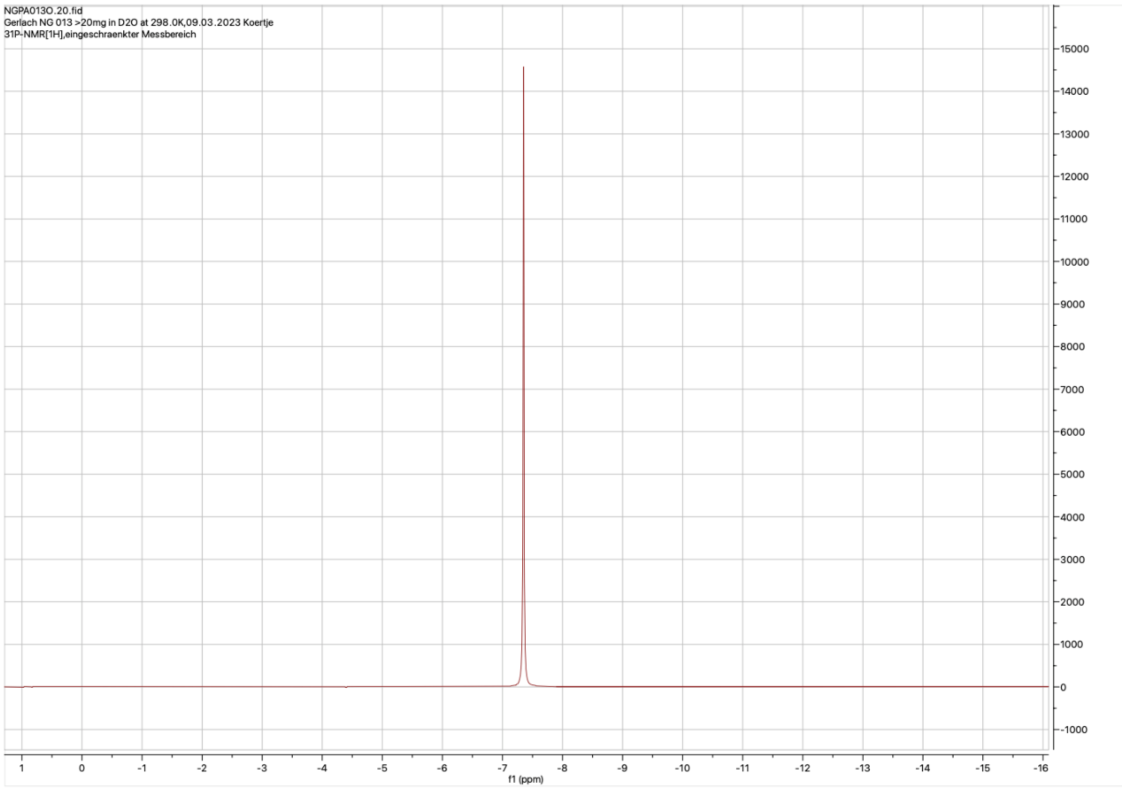

Figure S70 ^{31}P-NMR of **19** in D_2O (162 MHz).

15.2 Farnesylpyrophosphate 20

Farnesylpyrophosphate **20** was synthesized following Oberhauser et al.[22] The reaction was carried out under N_2 atmosphere. To tris(tetrabutylammonium)hydrogenphosphate **19** (1.0 g, 105 mmol, 2.5 eq.) in acetonitrile (5.0 mL) was added dropwise a solution of *trans, trans*-farnesylbromide (120 mg, 42 mmol. 1.0 eq.) in acetonitrile (3.5 mL). The reaction mixture was stirred at 24 °C for 19 h. After removing the solvent under reduced pressure, the residue was dissolved in 5 mL buffer 1 (2 g NH_4HCO_3 in 2 % (*v/v*) isopropylalcohol). This solution was eluted with buffer through a column containing ion exchange resin (Amberchrom ® 50WX8, NH_4^+-form. 100-200 mesh). After lyophilization, the crude residue was dissolved in 2 mL of buffer 2 (50 mM $(NH_4)_2CO_3$), mixed with 8 mL acetonitrile:isoproylalcohol (1:1) and vortexed. After centrifugation (5000 × g, 10 min) the supernatant was removed, and the extraction was repeated two more times. Removal of the solvent under reduced pressure and lyophilization afforded farnesylpyrophosphate **20** as a light yellowish material (83.7 mg, 19.3 mmol, 46 %).
For equilibrating the ion exchange resin from H^+ to NH_4^+ form, the resin was washed with 2.5 times column volume 3M aqueous hydrochloric acid to achieve a pH of 1. After washing it with 4 times column volume with deionized H_2O the flow through should have a nearly neutral pH. To set the pH to 11, the column got washed with 3.5 times column volume 25 % ammonia solution (diluted 1:4 with H_2O). After using 8 times column volume deionized H_2O to adjust the column to pH 7, it was equilibrated with 150 mL buffer 1.

^{1}H-NMR (D_2O, 400 MHz): δ =5.47 (t, *J* = 4.1 Hz. 1H), 5.14-5.26 (m, 2H), 4.49 (t, *J* =6.7 Hz, 2H), 2.00-2.20 (m, 8H), 1.73 (s, 3H), 1.70 (s, 3H), 1.63 (s, 6H) ppm. ^{31}P-NMR (D_2O, 162 MHz): δ = -10.30 (d, *J* = 20.9 Hz, 1P), -9.34 (d, *J* = 20.9 Hz, 1P) ppm.

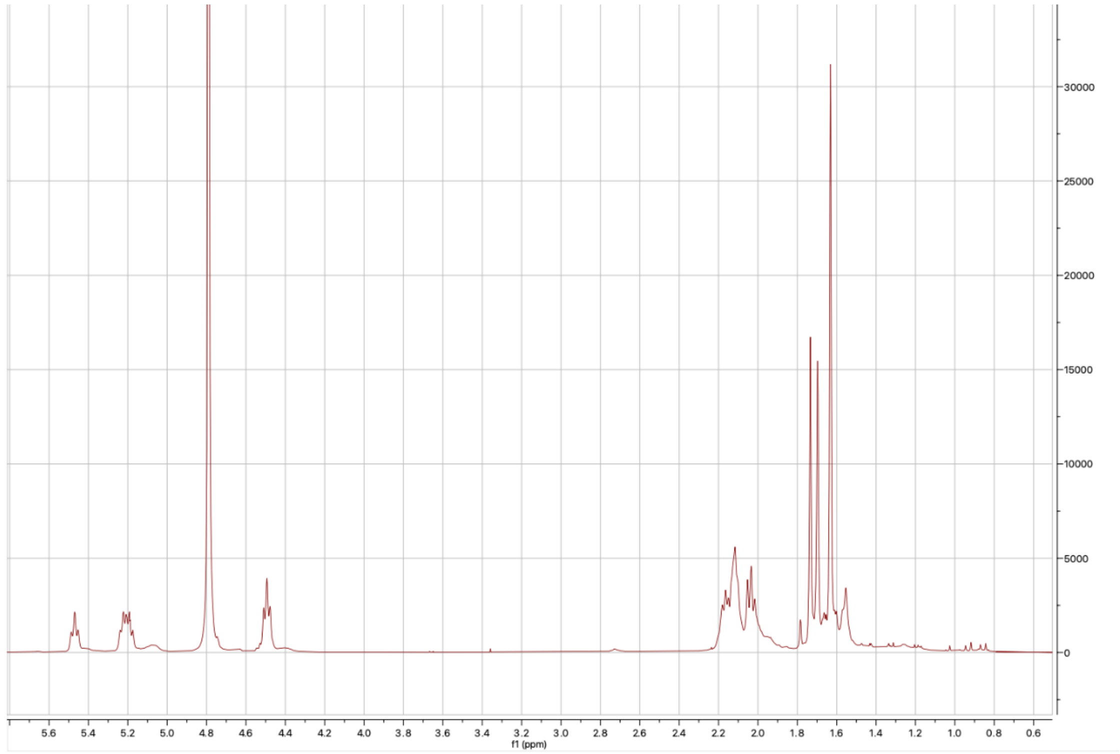

Figure S71 ^{1}H NMR of **20** in D_2O (400 MHz).

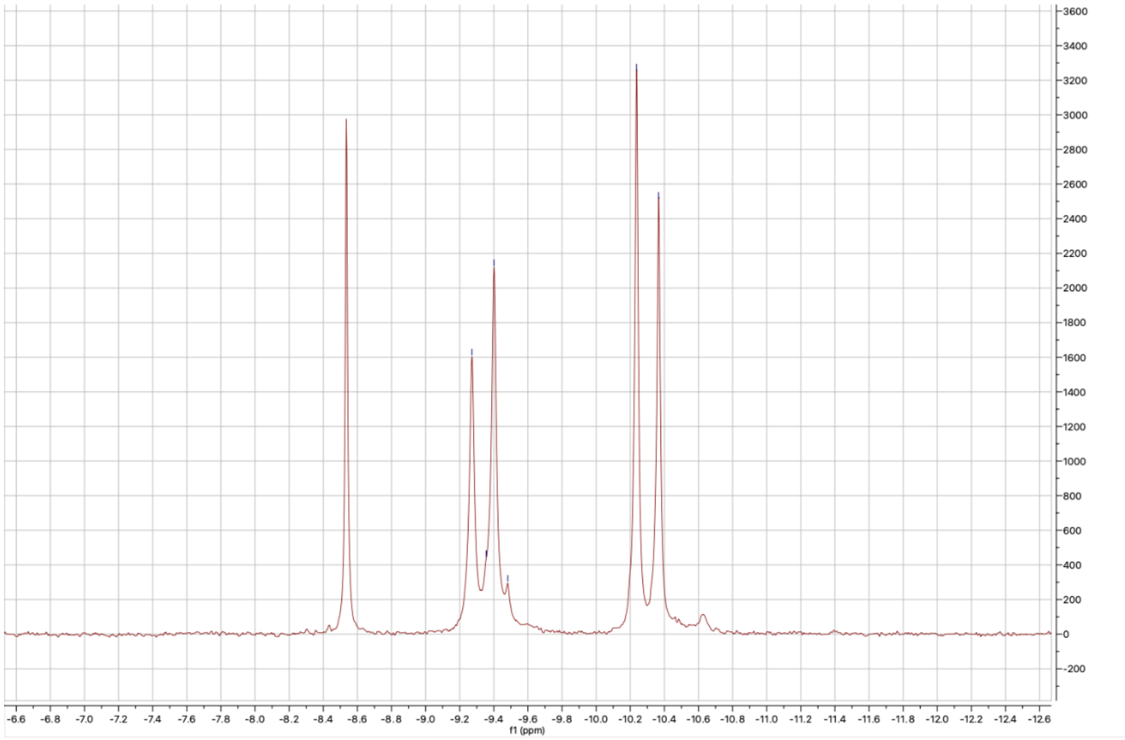

Figure S72 ^{31}P-NMR of **20** in D_2O (162 MHz).

16. Bioactivity Assays

The inhibitory activity of the alkyl citrates was determined using a squalene synthase (SQS) assay based on the consumption of NADPH.[23] and a phospholipase A2 (PLA_2) assay based on Holzer et al.[24] SQS fluorescence assays were performed on an Agilent BioTek Synergy HTX Multimode Reader. PLA_2 assays were performed on a SpectraMax Plus 384 Microplate Reader.

16.1 Squalene Synthase Assay

The synthesis of squalene by *Aspergillus oryzae* Squalene Synthase (SQSAo) was monitored in a fluorescence based assay by using an excitation wavelength of 360 nm.[23] The emission at 460 nm was measured. SQSAo was purified from *E. coli* after heterologous expression of the corresponding gene. The reactions were performed in a black walled 96-well plate in triplicates in 200 µL reaction volume containing 25 mM Tris-HCl (pH 7.4), 1 mM $MgCl_2$, 1 mM Triton X-100, 25 µM NADPH, 25 µM FPP and 5 µM SQSAo. Tested substrates were dissolved in DMSO; final concentration of DMSO in the assay was 5 % (*v/v*).

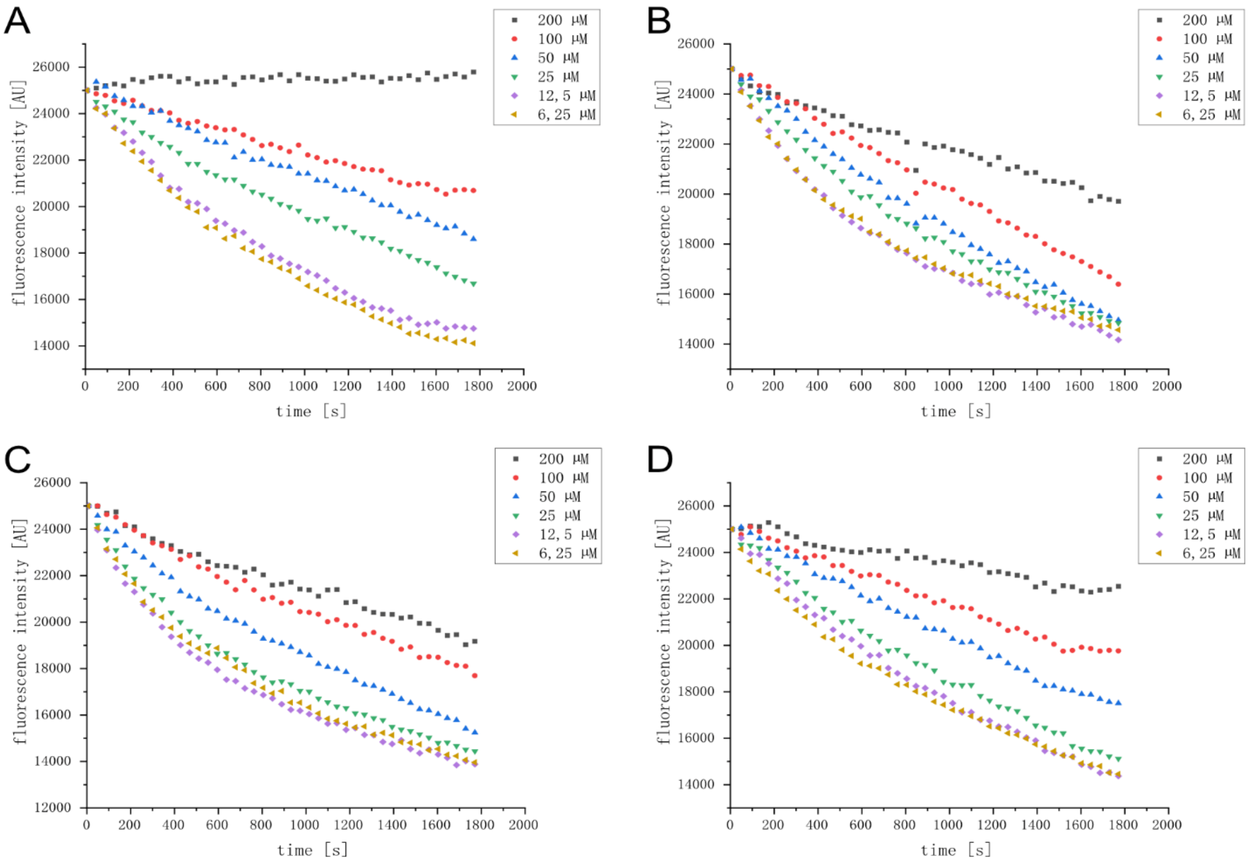

Figure S73 Raw data of squalene synthase inhibitory assays. **A**, concentration dependent inhibition of squalene synthase by the mixture of **9** and **8**. **B**, concentration dependent inhibition of squalene synthase by **7**. **C**, concentration dependent inhibition of squalene synthase by **14**. **D**, concentration dependent inhibition of squalene synthase by **15**.

16.2 Phospholipase A_2 Assay

The PLA_2 assay was performed by using the chromogenic substrate 4-nitro-3-(octanoyloxy)benzoic acid (NOBA), which undergoes hydrolysis to form 3-hydroxy-4-nitrobenzoic acid.[24] The absorption at 425 nm was monitored continuously to track the hydrolysis of NOBA. PLA_2 from porcine pancreas was purchased from Sigma-Aldrich. The reactions were performed in a 96-well plate in triplicates in 200 µL reaction volume containing 10 mM Tris-HCl (pH 8.0), 10 mM $CaCl_2$, 100 mM NaCl, 10 µg porcine pancreas PLA_2 and 50 µM NOBA. Tested substrates were dissolved in DMSO, final concentration of DMSO in the assay was 5 % (*v/v*).

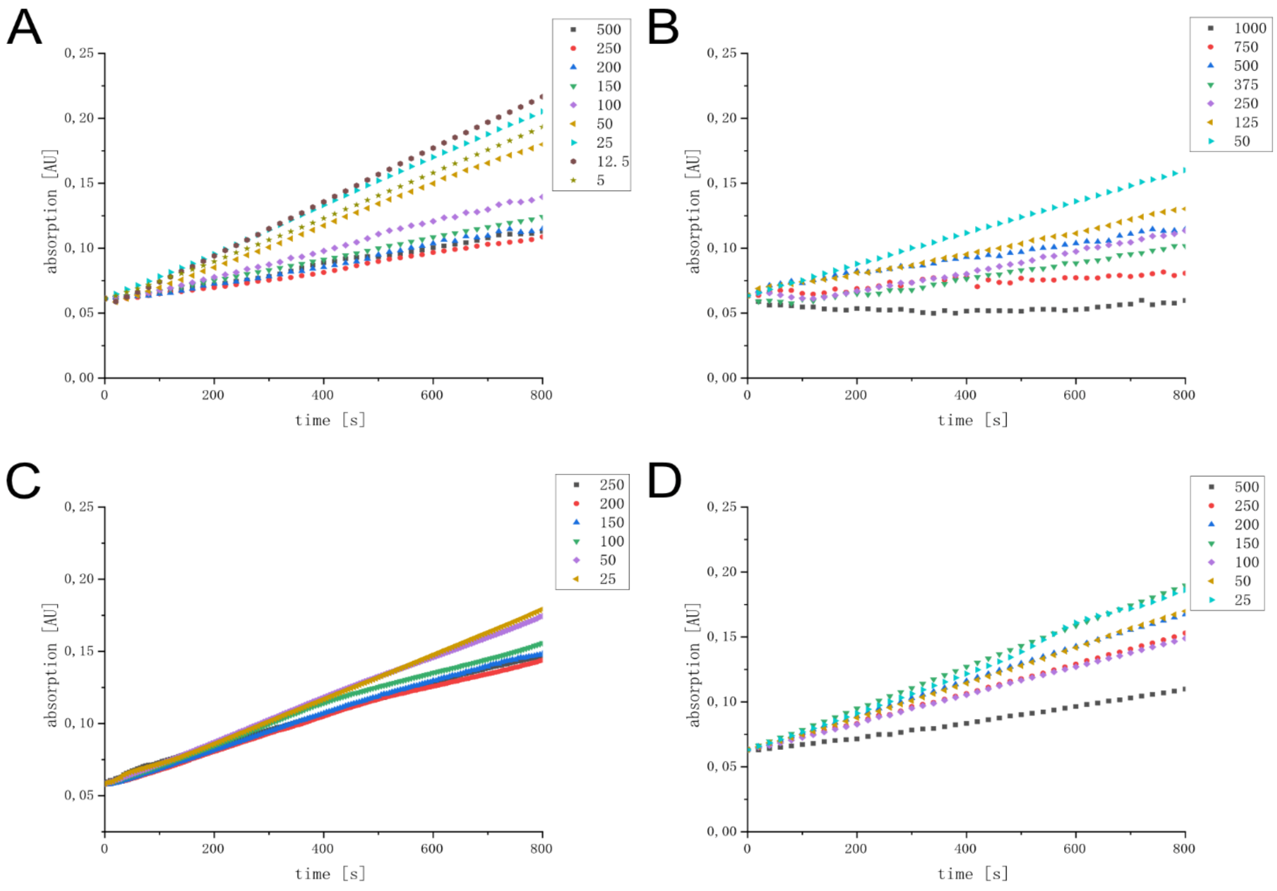

Figure S74 Raw data of phospholipase A_2 inhibitory assays. **A**, concentration dependent inhibition of phospholipase A_2 by by the mixture of **9** and **8**. **B**, concentration dependent inhibition of phospholipase A_2 by **7**. **C**, concentration dependent inhibition of phospholipase A_2 by **14**. **D**, concentration dependent inhibition of phospholipase A_2 by **15**.

17. Comparison and Interpretation of CtrR1 and MfR1/2 Activity *in vitro*

17.1 Proposed Oxidative Pathway for *in vitro* Reactions of CJ-13,982 with Trx-MfR1 and Trx-MfR2

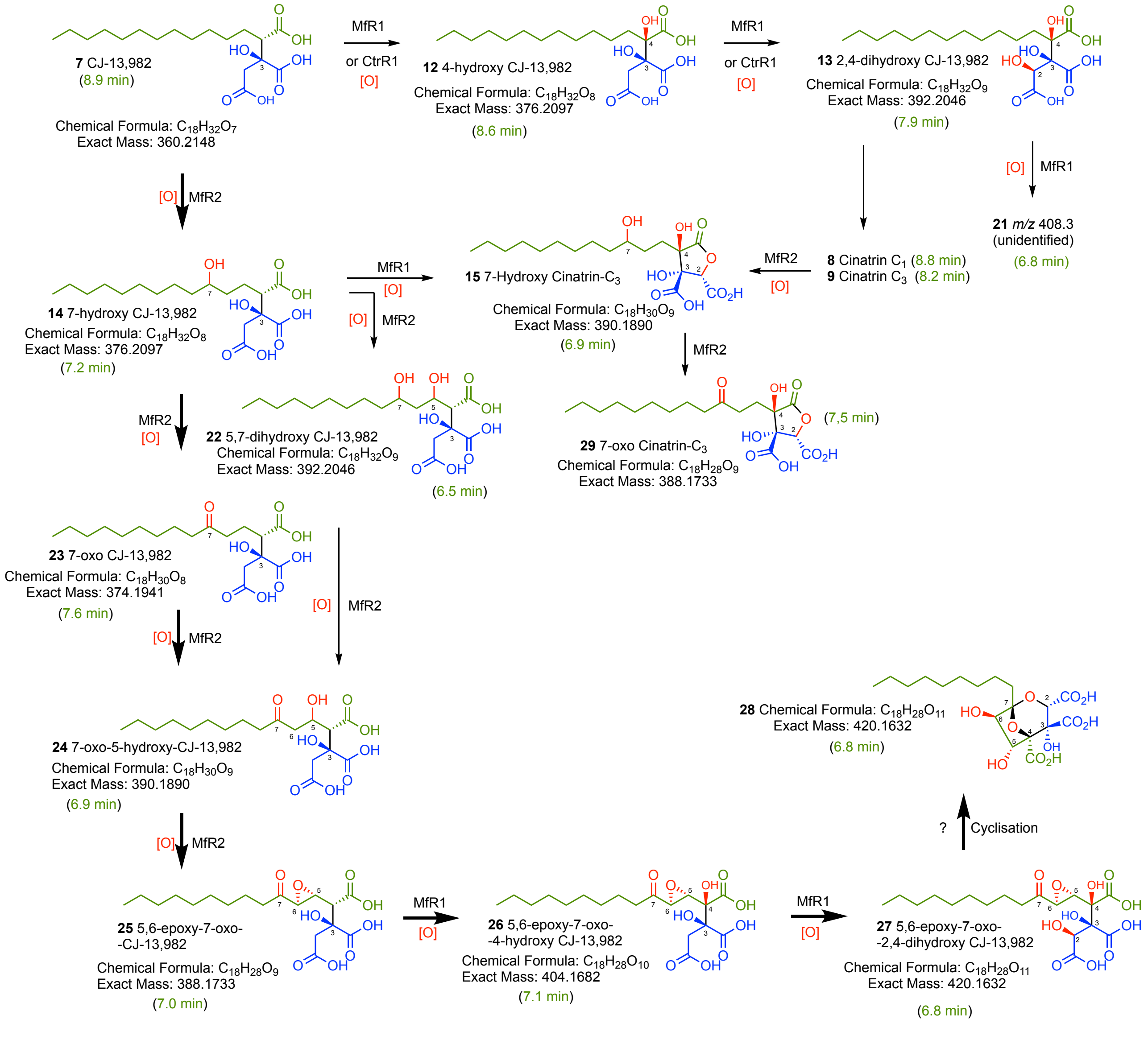

Figure S75 Oxidative pathway consistent with observed LCMS masses for *in vitro* reactions of CJ-13,982 with TrxA-His$_6$-Mfr1 and TrxA-His$_6$-Mfr2. See Figs S48 - S50, Fig S58 & Fig S62 for observed retention times and masses after LCMS analysis of *in vitro* reactions. Bold arrows indicate proposed main pathway.

17.2 Revision of Oxidative Steps during the Biosynthesis of Squalestatin S1

In previous work, studies of the activity of MfR1 and MfR2 from the squalestatin S1 pathway could not be performed *in vitro* because the native proteins could not be obtained in soluble or active form.[13] Evidence for their activity was inferred from LC-HR-MS studies of *in vivo* reconstruction of partial squalestatin biosynthetic pathways in *A. oryzae*. Observation of an $[M - 132 - H]^-$ fragment ion in the MSMS data had been suggested to be linked to loss of oxaloacetate **2** from the oxidatively-modified alkyl citrates *via* the mechanism shown in Fig. S76A. This, in turn, had been suggested to indicate that oxidative modifications must have occurred at the polyketide backbone and not in the oxalic-acid-derived moiety.

However, it must be noted that neither in this study for CJ-13,982 **7** (Fig. S76B) nor previously for squalestatin S1 intermediate L-731,120 **10** (Fig. S76C), was loss of 132 Da observed for **7** (359 -132 = 227 Da) or for **10**: (419 - 132 = 287 Da). This would be expected if the assumptions made in the Squalestatin S1 **11** study were correct. Hence, observation of specific ions in the MSMS data appears to be an inadequate method for localization of the oxidative modifications for the alkyl citrates. Consequently, incorrect assumptions on the oxidations catalyzed by MfR1 and MfR2 during the biosynthesis of squalestatin S1 **11** were made previously.

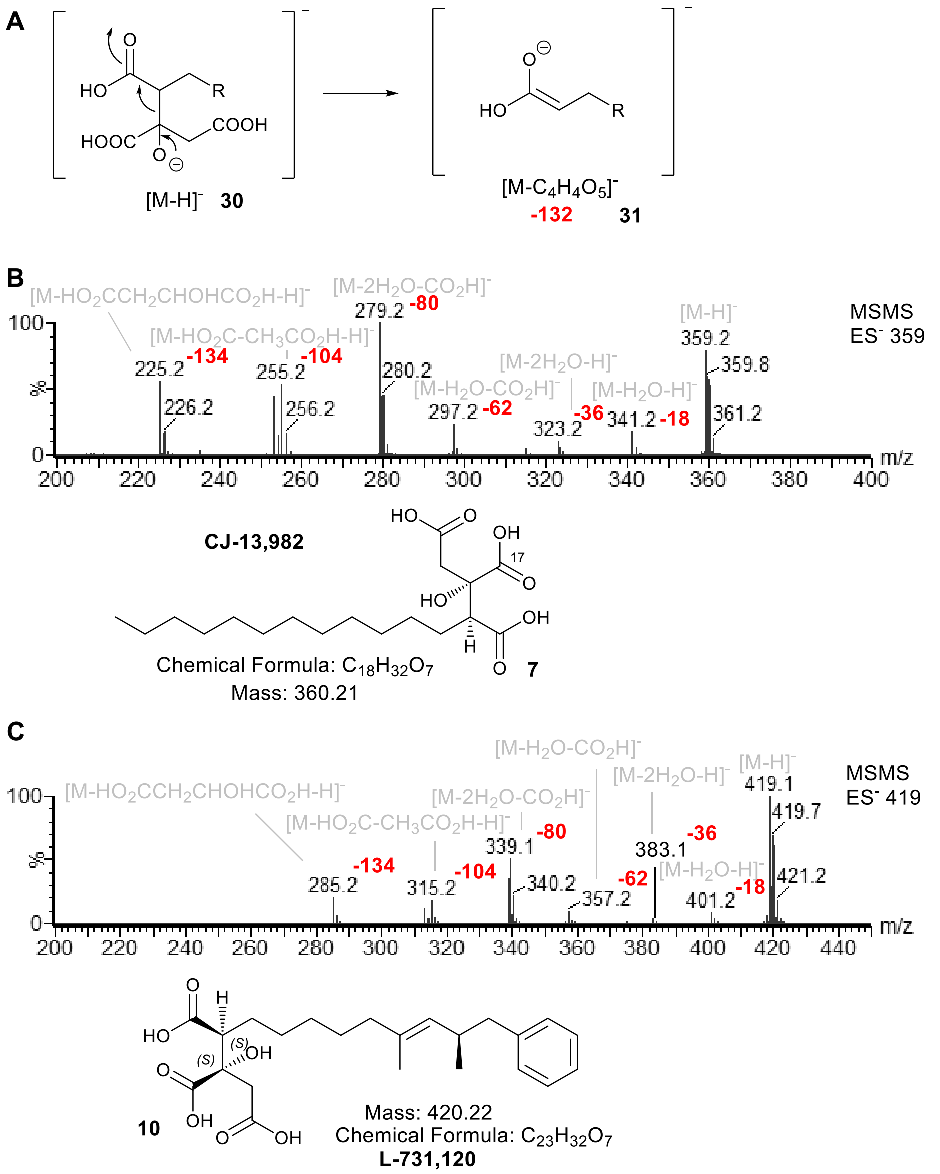

Figure S76 Comparison of MSMS data of unmodified alkyl citrates. **A**, previously proposed mechanism for loss of oxaloacetate; **B**, MSMS data of CJ-13,982 **7** obtained in this study; **C**, MSMS data of L-731,120 **10** obtained in previous studies.[13] Red numbers indicate loss in mass compared to $[M - H]^-$ ion.

Notably, for CJ-13,982 **7** and L-731,120 **10** the same fragmentation patterns were observed (Fig. S76, compare red numbers). Fig. S77 summarizes fragmentation reactions we now suggest for the formation of the observed fragments **32** [M - H_2O - H]$^-$, **33** [M - $2H_2O$ - H]$^-$, **34** [M - $2H_2O$ - CO_2H]$^-$, **36** or **38** [M - H_2O - CO_2H], **41** [M - HO_2C- CH_3CO_2H - H]$^-$ and **42** [M - $HO_2CCH_2CHOHCO_2H$ - H]$^-$ starting from the [M - H]$^-$ ion **7** or **10**.

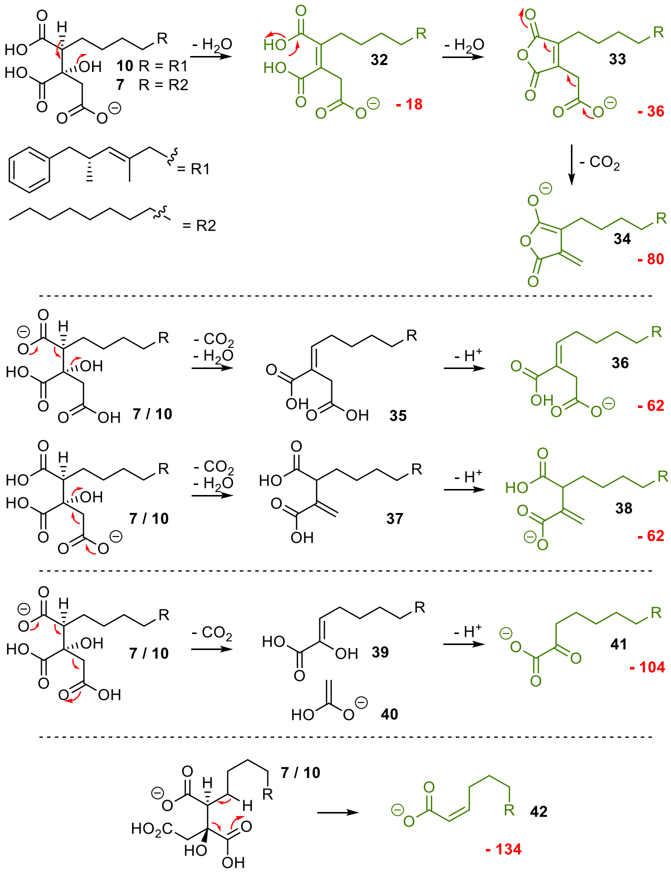

Figure S77 Observed fragmentations of **7** and **10** under ES$^-$ conditions. Proposed fragment ions are shown in green and mass loss in red. Mass losses match those observed in Fig. S76.

In the course of this study, we confirmed by NMR that MfR1 catalyzes modifications at C-2 and C-4 of CJ-13,982 **7** and that MfR2 modifies the alkyl chain. The investigations on the *ctr* BGC revealed lactonization of the 2,4-dihydroxylated alkyl citrate **13**. We used this knowledge to reinterpret the HRMS data obtained in the previous study on the oxidative steps during squalestatin S1 **10** biosynthesis.[13]

In a targeted knockout (KO) experiment in *Phoma* MF5453, *mfr2* has been deleted. In the knockout strain, a compound **43** with *m/z* ES$^-$ [M - H]$^-$ = 435.2019 was observed.[13] We now suggest that compound **43** is 4-hydroxy-L-731,210 as our *in vitro* study demonstrated that MfR1 hydroxylates C-4 (Fig. S78). Another compound **44b** with *m/z* ES$^-$ [M - H]$^-$ = 433.1862 Da was also observed,[13] which we suggest to be a lactonized derivative of 2,4-dihydroxy-L-731,210 **44a** (Fig. S78).

For heterologous expression studies, *A. oryzae* was transformed with genes for the production of L-731,120 **10** (*sqhks, mfr3, mfm8*) and *mfr1* and *mfr2*. In the respective transformant *m/z* [M - H]$^-$ = 479.1553 Da was observed. This ion was identified as highly oxygenated compound **50** by NMR in the previous study.[13] In addition, a compound **47b** with *m/z* [M - H]$^-$ = 463.1604 Da was observed.[13] Here, we propose that this compound could be a derivative of 2,4-dihydroxy-7-oxo-L-731,210 **46a** hydroxylated at C-5 if designated for epoxide formation as in **48a/b** (Fig. S78). The keto-function at C-7 could possibly be introduced by Mfr2, *via* hydroxylation at C-7 first (*e.g.* **45a/b** Fig. S78).

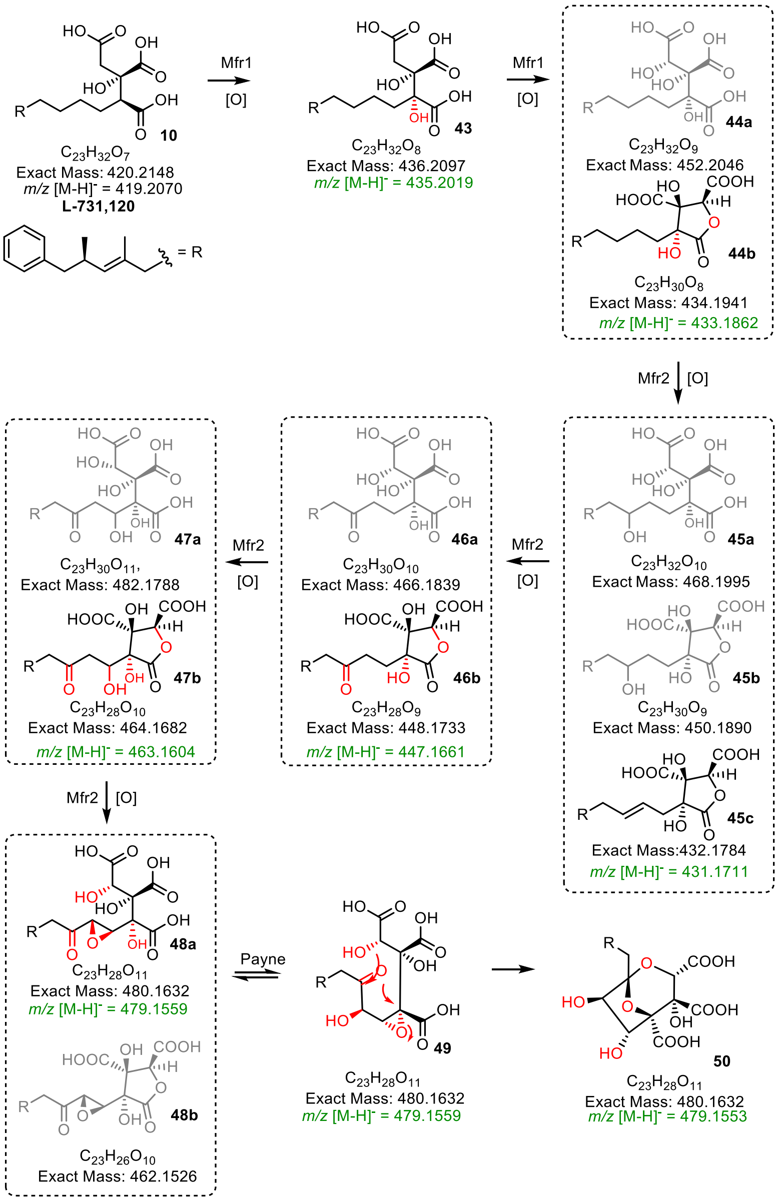

Figure S78 Reinterpretation of HRMS data obtained in studies on the oxidative steps during the biosynthesis of squalestatin S 1 **11**.[13] For 2,4-dihydroxylated compounds the lactonized (only one possible lactonization product is shown) and opened form are shown in dashed boxes. Compounds in grey were not directly observed. Stereochemistry of intermediates is based on known stereochemistry of squalestatin S1 **11**.

18. Literature

1. D. Wibberg, M. Stadler, C. Lambert, B. Bunk, C. Spröer, C. Rückert, J. Kalinowski, R. J. Cox and E. Kuhnert, *Fungal Divers*, 2021, **106**, 7–28.
2. Jonathan M. Palmer, & Jason Stajich. (2020). Funannotate v1.8.1: Eukaryotic genome annotation (v1.8). Zenodo. https://doi.org/10.5281/zenodo.1134477
3. E. Kuhnert, J. C. Navarro-Muñoz, K. Becker, M. Stadler, J. Collemare and R. J. Cox, *Studies in Mycology*, 2021, **99**, 1–43.
4. S. F. Altschul, T. L. Madden, A. A. Schaffer, J. Zhang, Z. Zhang, W. Miller and D. J. Lipman, *Nuc. Acids Res*., 1997, 25, 3389–3402.
5. D. S. Tian, E. Kuhnert, J. Ouazzani, D. Wibberg, J. Kalinowski and R. J. Cox, *Chem. Sci.*, 2020, **11**, 12477–12484.
6. C. L. M. Gilchrist and Y. H. Chooi, *Bioinformatics*, 2021, **37**, 2473–2475.
7. T. Mori, R. Zhai, R. Ushimaru, Y. Matsuda and I. Abe, *Nat Commun*, 2021, **12**, 4417.
8. Y. Matsuda, T. Bai, C. B. Phippen, C. S. Nødvig, I. Kjærbølling, T. C. Vesth, M. R. Andersen, U. H. Mortensen, C. H. Gotfredsen, I. Abe and T. O. Larsen, *Nature Communications*, 2018, **9**, 2587.

9 J. S. Papadopoulos and R. Agarwala, *Bioinformatics*, 2007, **23**, 1073–1079.

10. K. A. K. Pahirulzaman, K. Williams and C. M. Lazarus, *Meth. Enzymol.*, 2012, **517**, 241–260.
11. L. Wendt, E. B. Sir, E. Kuhnert, S. Heitkämper, C. Lambert, A. I. Hladki, A. I. Romero, J. J. Luangsa-ard, P. Srikitikulchai, D. Peršoh and M. Stadler, *Mycological Progress*, 2018, **17**, 115–154.
12. R. D. Gietz and R. A. Woods, *Methods in Enzymology*, 2002, **350**, 87–96.
13. K. E. Lebe and R. J. Cox, *Chem. Sci.*, 2019, **10**, 1227–1231.
14. F. J. Jin, J. I. Maruyama, P. R. Juvvadi, M. Arioka and K. Kitamoto, *FEMS Microbiology Letters*, 2004, **239**, 79–85
15. A. G. Ferrige, M. J. Seddon, B. N. Green, S. A. Jarvis, J. Skilling and J. Staunton, *Rapid Communications in Mass Spectrometry*, 1992, **6**, 707–711.
16. A. G. Ferrige, M. J. Seddon, S. Jarvis, J. Skilling and R. Aplin, *Rapid Communications in Mass Spectrometry*, 1991, **5**, 374–377.
17. D. Sturgess, Z. Chen, J. M. White and M. A. Rizzacasa, *Journal of Antibiotics*, 2018, **71**, 234–239.
18. L. Atkin, Z. Chen, A. Robertson, D. Sturgess, J. M. White and M. A. Rizzacasa, *Organic Letters*, 2018, **20**, 4255–4258.
19. S. Watanabe, H. Hirai, T. Kambara, Y. Kojima, N. Hiroyuki, S. Ayumu, Y. Yuji, Y. Nobuji, J. H. James, Harwood, H. Liang, Hsuing and K. Nakao, *The journal of antibiotics*, 2001, **54**, 1025–1030.
20. A. N. Cuzzupe, R. Di Florio, J. M. White and M. A. Rizzacasa, *Org. Biomol. Chem.*, 2003, **1**, 3572–3577.
21. H. Itazaki, K. Nagashima, Y. Kawamura, K. Matsumoto, H. Nakai and Y. Terui, *J. Antibiot.*, 1992, **45**, 38–49.
22. C. Oberhauser, V. Harms, K. Seidel, B. Schröder, K. Ekramzadeh, S. Beutel, S. Winkler, L. Lauterbach, J. S. Dickschat and A. Kirschning, *Angew. Chem. Int. Ed*., 2018, 57, 11802–11806.
23. A. D. Napper and S. Sivendran, *Curr. Protoc. Chem. Biol.*, 2011, **3**, 81–97.
24. M. Holzer and S. P. Mackessy, *Toxicon*, 1996, 34, 1149–1155.
